# Methanogen activity and microbial diversity in Gulf of Cádiz mud volcano sediments

**DOI:** 10.3389/fmicb.2023.1157337

**Published:** 2023-05-24

**Authors:** Gordon Webster, Barry A. Cragg, Joachim Rinna, Andrew J. Watkins, Henrik Sass, Andrew J. Weightman, R. John Parkes

**Affiliations:** ^1^Microbiomes, Microbes and Informatics Group, School of Biosciences, Cardiff University, Cardiff, Wales, United Kingdom; ^2^School of Earth and Environmental Sciences, Cardiff University, Cardiff, Wales, United Kingdom; ^3^Aker BP ASA, Lysaker, Norway; ^4^The Wales Research and Diagnostic Positron Emission Tomography Imaging Centre (PETIC), School of Medicine, Cardiff University, University Hospital of Wales, Cardiff, Wales, United Kingdom

**Keywords:** methanogens, marine sediments, biogeochemical cycles, mud volcanoes, *Archaea*, anaerobic methane oxidation, *Bacteria*

## Abstract

The Gulf of Cádiz is a tectonically active continental margin with over sixty mud volcanoes (MV) documented, some associated with active methane (CH_4_) seepage. However, the role of prokaryotes in influencing this CH_4_ release is largely unknown. In two expeditions (MSM1-3 and JC10) seven Gulf of Cádiz MVs (Porto, Bonjardim, Carlos Ribeiro, Captain Arutyunov, Darwin, Meknes, and Mercator) were analyzed for microbial diversity, geochemistry, and methanogenic activity, plus substrate amended slurries also measured potential methanogenesis and anaerobic oxidation of methane (AOM). Prokaryotic populations and activities were variable in these MV sediments reflecting the geochemical heterogeneity within and between them. There were also marked differences between many MV and their reference sites. Overall direct cell numbers below the SMTZ (0.2–0.5 mbsf) were much lower than the general global depth distribution and equivalent to cell numbers from below 100 mbsf. Methanogenesis from methyl compounds, especially methylamine, were much higher than the usually dominant substrates H_2_/CO_2_ or acetate. Also, CH_4_ production occurred in 50% of methylated substrate slurries and only methylotrophic CH_4_ production occurred at all seven MV sites. These slurries were dominated by *Methanococcoides* methanogens (resulting in pure cultures), and prokaryotes found in other MV sediments. AOM occurred in some slurries, particularly, those from Captain Arutyunov, Mercator and Carlos Ribeiro MVs. Archaeal diversity at MV sites showed the presence of both methanogens and ANME (*Methanosarcinales*, *Methanococcoides*, and ANME-1) related sequences, and bacterial diversity was higher than archaeal diversity, dominated by members of the *Atribacterota*, *Chloroflexota*, *Pseudomonadota*, *Planctomycetota*, *Bacillota*, and Ca. “*Aminicenantes*.” Further work is essential to determine the full contribution of Gulf of Cádiz mud volcanoes to the global methane and carbon cycles.

## Introduction

Submarine mud volcanoes (MV) are seafloor diapir structures that exhibit episodic extrusion of mud, fluids and gases (predominantly methane) from subsurface reservoirs into the overlying strata and water column (Milkov, 2000; Dimitrov, 2003; Joye, 2020). They are formed as a result of different geological processes acting at active, convergent plate boundaries and passive continental margins resulting in high pore fluid pressures, sediment instability and consequently mud and gas extrusion (Kopf, 2002). MVs are structurally diverse, ranging in shape from amorphous mud pies to conical structures, and in size from a few meters to kilometers in diameter, and can be hundreds of meters high (Dimitrov, 2002; Kopf, 2002). They are geochemically variable, differing in the range of hydrocarbons released (Joye, 2020), the source of methane (Nuzzo et al., 2009), salinity (Haffert et al., 2013), and have varying periods of quiescence that allow pelagic material to build up from sedimentation of the water column (Kopf, 2002; Palomino et al., 2016). Despite the mechanisms for MV formation being well known, the actual global distribution and number of submarine MVs is not, with documented occurrences >1,000 MVs (Kopf, 2003; Baloglanov et al., 2018) and suggestions that global numbers are far greater and could be between 10^3^ and 10^5^ MVs (Milkov, 2000). Such large numbers of submarine MVs represent a significant and largely under investigated source of carbon input into overlying sediments, deep ocean, water column and the atmosphere (Milkov, 2000; Kopf, 2002; Dimitrov, 2003; Sauter et al., 2006; Joye, 2020). Estimates of global methane emissions from MVs vary, but it is thought to be in the tens of teragrams of methane per year and may be as much as 10% of the total annual methane emission to the atmosphere (Judd, 2005; Wallmann et al., 2006; Etiope, 2009; Etiope et al., 2011; Mazzini and Etiope, 2017).

Most of the gas venting from submarine MVs is composed of thermogenic methane (Kopf, 2002), but a significant fraction is of mixed or of microbial origin (Mazzini and Etiope, 2017) produced *in situ* by methanogenic archaea. Methanogenesis is the terminal oxidation process in the anaerobic degradation of organic matter and methanogens can be divided into three metabolic groups based on substrates used: hydrogenotrophic methanogens using H_2_/CO_2_, acetoclastic methanogens utilizing acetate and methylotrophic methanogens using methylated compounds, such as methanol, methylamine and methyl sulphide (Liu and Whitman, 2008; Bueno de Mesquita et al., 2023). In marine sediments, it is generally thought that the dominant methanogenic process is the reduction of CO_2_ by H_2_ (e.g., Mah et al., 1977; Claypool and Kvenvolden, 1983; Whiticar et al., 1986; Parkes et al., 2007; Beulig et al., 2018), although some studies suggest that acetate is also an important substrate for methanogenesis in subsurface sediments (Crill and Martens, 1986; Fry et al., 2008). However, the dominant methanogenic process occurring within MV sediments remains relatively unknown with only a few studies having specifically investigated methanogenic populations (Lazar et al., 2011b, 2012; Maignien et al., 2013). The main focus of MV microbiological studies has been to investigate prokaryotic diversity and the role microbial communities play in the anaerobic oxidation of methane (AOM; e.g., Pancost et al., 2000; Martinez et al., 2006; Niemann et al., 2006a,b; Heijs et al., 2007; Pachiadaki et al., 2011; Ruff et al., 2019), and how bacteria provide energy to support micro- and macrofaunal ecosystems (e.g., Lösekann et al., 2008; Rodrigues et al., 2010; Coelho et al., 2016; Palomino et al., 2016). In marine sediments, the metabolic process of AOM has been proposed to be due to reversed methanogenesis coupled to the reduction of sulphate involving anaerobic methane-oxidizing archaea (ANME) and sulphate-reducing bacteria (SRB). ANME and SRB are thought to interact syntrophically forming microbial consortia that anaerobically oxidize methane with equimolar amounts of sulphate, yielding bicarbonate and sulphide (Knittel and Boetius, 2009). Sulphide can then be used to support chemosynthetic macrofauna that derive energy from sulphide oxidation by symbiotic bacteria (Stewart et al., 2005).

In this study we investigated a range of mud volcanoes from different sea water depths along a transect of the Gulf of Cádiz with the aim to understand *in situ* methanogen populations, their activity, substrate use and diversity in variable mud volcano habitats. Anaerobic sediment slurry enrichments were established from sediment samples taken at varying depths from seven MVs and incubated with a wide range of potential methanogenic substrates (acetate, benzoate, hexadecane, methanol, methylamine and H_2_/CO_2_) for more than 130 days, after which total methane production was analyzed. Methane producing enrichments were further analyzed for their dominant archaeal and bacterial community structure using 16S rRNA and methanogen-specific *mcrA* genes. In addition, geochemical, methanogenic activity, total cell numbers and microbial diversity data (16S rRNA genes) from MV sediments was also collected.

## Materials and methods

### Site description and sample collection

**Figure 1:**
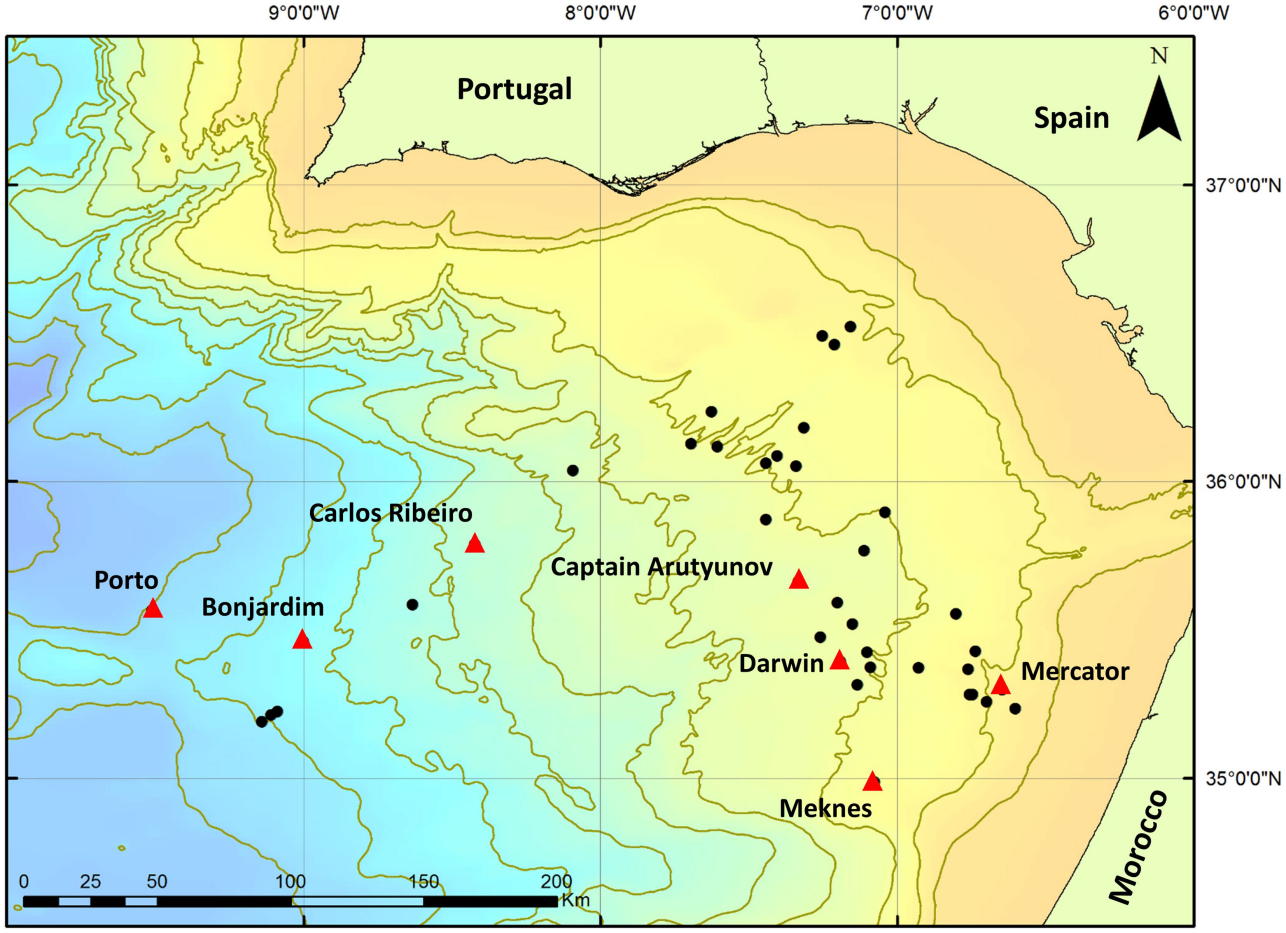
Location of Gulf of Cádiz mud volcanoes sampled during expeditions MSM1-3 and JC10. Red triangles indicate mud volcanoes used in this study. Black circles indicate other mud volcanoes located in the area.

The Gulf of Cádiz (Figure 1) is located in the NE Atlantic Ocean, west of the Gibraltar Arc between the Iberian Peninsula and the Moroccan margin. The area has experienced a complex geological history related to plate tectonic interactions which have resulted in several episodes of rifting, compression and strike-slip motion due to the closure of the Tethys Ocean, the opening of the N Atlantic, and the African-Eurasian convergence since the Cenozoic (Maldonado et al., 1999). Due to this ongoing compression, rapidly deposited sediments have been dewatered progressively and formed many MVs and fluid escape structures (Díaz-del-Rio et al., 2003). Since their discovery in 1999 (Gardner, 2001) more than 60 MVs have now been identified (León et al., 2012), ranging from 800 to 3,500 m in diameter and some as high as 300 m above the seafloor. Most MVs in the area have been built up from episodes of mud-breccia flows (Medialdea et al., 2009) and show evidence of gas saturation, methane hydrate, hydrogen sulphide and the presence of chemosynthetic fauna (e.g., Somoza et al., 2003; Niemann et al., 2006a; Rodrigues et al., 2010, 2011).

Sediment cores were collected from seven mud volcanoes (Porto, Bonjardim, Carlos Ribeiro, Captain Arutyunov, Darwin, Meknes and Mercator MV) from the Gulf of Cádiz (Figure 1) during the EU HERMES project expedition cruises MSM1-3 on the Research Vessel Maria S. Merian (12^th^ April – 19^th^ May 2006) and JC10 (Leg 1) on the Royal Research Ship James Cook (14^th^ May to 2^nd^ June 2007) using a range of sampling methods and devices (Table 1). Additional sediment cores were also taken during Expedition JC10 from reference seafloor sites >1 km away from each MV sampled.

**Table 1:** Collection data for the sediment samples used in this study.

Mud volcano	Expedition	Station	Site description	Sample method^a^	Latitude (N)	Longitude (W)	Water depth (m)	Sediment sample depth (mbsf) used		
								AODC, activity, geochemistry	Methanogen enrichment slurries	DNA analysis
Porto	MSM1-3	144	center	GC	35°33.703’	09°30.433’	3,860	0.88–1.23	0.77, 1.12	0.90
	MSM1-3	160	center	MC	35°33.751’	09°30.501’	3,863	0.01–0.34	–	–
Bonjardim	MSM1-3	131	center	GC	35°27.814’	09°00.134’	3,048	0.33–3.08	0.42, 1.67, 2.47, 2.97	0.35, 1.25, 1.60, 2.45, 3.1
Carlos Ribeiro	JC10	044	reference	MC	35°46.042’	08°26.554’	2,344	0.02–0.18	–	–
	JC10	045	reference	PC	35°46.044’	08°26.556’	2,345	0.17–4.48	–	–
	JC10	048	rim	GC	35°47.225’	08°25.292’	2,177	0.15–1.46	–	–
	JC10	050	rim	MC	35°47.221’	08°25.292’	2,176	0.02–0.15	–	–
	JC10	053	center	PC	35°47.259’	08°25.320’	2,174	0.13–5.24	0.30, 0.55, 1.05, 4.25, 5.15	0.1, 0.3, 1.35, 5.15
	JC10	054	center	MC	35°47.300’	08°25.219’	2,179	0.02–0.48	–	–
Captain Arutyunov	MSM1-3	191	center	GC	35°39.639’	07°20.049’	1,322	0.43–2.63	0.52, 1.77, 2.67	0.45, 1.7, 2.60
	MSM1-3	206	center (W)	GC	35°39.696’	07°20.080’	1,326	0.68–2.31	0.77, 1.12, 2.22, 2.82	0.7, 1.05, 2.75
	MSM1-3	227	center (E)	GC	35°39.699’	07°20.001’	1,319	0.25–1.68	0.77, 1.77	0.7, 1.15
	JC10	066	center	PC	35°39.637’	07°20.046’	1,311	0.05–4.02	3.93	0.25, 0.58, 3.98
Darwin	JC10	025	reference	PC	35°23.965’	07°11.120’	1,145	0.30–4.81	–	–
	JC10	026	reference	MC	35°23.965’	07°11.121’	1,145	0.02–0.36	–	–
	JC10	029	rim	GC	35°23.534’	07°11.456’	1,104	0.02–0.38	–	0.15
	JC10	030	rim	MC	35°23.537’	07°11.454’	1,104	0.01–0.23	–	–
	JC10	036	rim	ISIS	35°23.541’	07°11.509’	1,112	0.01–0.20	0.11	–
	JC10	038	rim	GC	35°23.473’	07°11.493’	1,105	0.04–0.70	0.18, 0.48	0.47
Meknes	MSM1-3	306	center	GC	34°59.137’	07°04.404’	694	1.18–2.03	0.77, 1.82	1.2, 2.05
Mercator	MSM1-3	238	center	GC	35°17.916’	06°38.700’	353	0.83–1.63	0.72, 1.72	0.85, 1.65, 2.05
	JC10	002	reference	MC	35°17.260’	06°40.240’	470	0.02–0.23	0.05, 0.20	–
	JC10	004	reference	PC	35°17.244’	06°40.251’	470	0.42–3.51	–	–
	JC10	009	center	GC	35°17.840’	06°38.820’	349	0.02–0.73	0.13	–
	JC10	011	rim	MC	35°17.920’	06°38.700’	351	0.02–0.38	0.04	–
	JC10	013	rim	MC	35°17.810’	06°38.770’	348	0.02–0.27	0.15	–
	JC10	015	rim	GC	35°17.806’	06°38.771’	346	0.12–0.78	0.30, 0.48, 0.75	–
	JC10	019	center	PC	35°17.866’	06°38.797’	346	0.12–2.23	0.18, 0.38, 1.90, 2.23	0.2, 0.4

a GC, Gravity corer; PC, Piston corer; MC, Mega corer; ISIS, ROV ISIS Push core.

On board ship, sediment cores from each MV site were sampled aseptically at different depths (ranging from 0.01 to 5.25 mbsf; meters below seafloor; Table 1) using sterile pre-cut (luer-end removed) 50 ml volume syringes. All syringe mini-cores were then capped and sealed inside gas-tight aluminum bags under anoxic conditions with a nitrogen atmosphere and an Anaerocult A (Merck; Cragg et al., 1992), and stored at 4°C or frozen directly at −80°C. On board ship, further sediment samples were taken for cell counts, sediment pore water chemistry, gas analysis and activity measurements (only cruise JC-10). All mini-cores, samples for AODC and chemical analyses were then transported back to the UK for processing. Note 4°C stored samples were used for methanogen enrichments and chemical analysis, and −80°C samples for molecular analysis.

### Acridine orange direct counts, chemical analysis, and prokaryotic activity measurements

Samples for AODC were fixed on board ship in filter-sterilized (0.2 μm) 4% (w/v) formaldehyde as described by Fry (1988). Preserved samples were then stained [0.1% (w/v) acridine orange] and counted on black polycarbonate membrane filters using a Zeiss Axioskop epifluorescence microscope.

Sediment for methane gas analysis was transferred on board ship into 20 ml volume serum vials with 10 ml of 10% (w/v) KCl, sealed and stored for equilibration. Headspace gas was analyzed using a Perkin Elmer/Arnel Clarus 500 natural gas analyzer with a flame ionization detector and a thermal conductivity detector. Methane and carbon dioxide concentrations were determined using a calibration with standard gases (Scott Specialty Gases). Pore waters were obtained from sediments on board ship using a pore-water squeezer as described (Wellsbury et al., 2002). Anions (including sulphate and acetate concentrations) were determined using an ICS-2000 Ion Chromatography System with two Ionpac AS15 columns in series and an Anion Self-Regenerating Suppressor (ASRS-ULTRA II 4 mm) in combination with a DS6 heated conductivity cell (Dionex UK Ltd.) as described (Webster et al., 2009). Cations (including methylamines) were analyzed using a DX-120 ion chromatograph (Dionex UK Ltd.) fitted with an IonPac CS16 column, a CSRS 300 4-mm suppressor, and a conductivity detector and methanesulfonic acid eluent (32 mM) at a flow rate of 0.75 ml min^−1^ (Watkins et al., 2012).

Radiotracer experiments using ^14^C-labelled substrates were carried out as previously described (Parkes et al., 2007, 2010). Briefly, sediment was subsampled using either Perspex mini-cores (diameter 2.0 cm, length 20 cm) with silicone-filled side injection holes (4 μl substrate per 1 cm depth interval) or 5-ml sterile pre-cut (luer-end removed) syringes closed with rubber stoppers (7.5 μl substrate per syringe). Samples were then preincubated in nitrogen-flushed gas-tight aluminum bags at 10°C to equilibrate for 24 h before radiotracer injection. Samples were injected with one of four ^14^C-substrates in batches of four (one control and three measurements) using either ^14^C-sodium bicarbonate (9.90 KBq μl^−1^), ^14^C-sodium acetate (6.60 KBq μl^−1^), ^14^C-methanol (2.05 KBq μl^−1^) or ^14^C-methylamine (7.36 KBq μl^−1^) and incubated at *in situ* bottom water temperature for 7 to 24 h in nitrogen-flushed gas-tight aluminum bags. Microbial reactions were stopped by transferring the incubated sediment into 30 ml glass serum vials containing 7 ml of 1 M NaOH and sealed. All samples were stored inverted at room temperature prior to processing. For ^14^C-CH_4_ analysis, samples were magnetically stirred whilst the headspace gas was flushed for 20 min with 5% O_2_: 95% N_2_ and passed over copper oxide at 900°C to convert ^14^C-CH_4_ to ^14^C-CO_2_. Flushed gases were bubbled through a series of three scintillation vials of 10 ml of Hi-Safe 3 scintillation cocktail (Canberra-Packard) containing 7% (v/v) ß-phenethylamine to absorb any ^14^C-CO_2_. Scintillation vials were counted in a scintillation counter and label turnover rates and potential activity rates calculated as described (Parkes et al., 2010). Methane production rates were calculated based on the proportion of labelled gas produced from the ^14^C-substrate, the dissolved pore water substrate or total CO_2_ concentration adjusted for sediment porosity, and the incubation time. For methylamine methanogenesis, rates were obtained by multiplying the substrate turnover value by an assumed methylamine concentration of 5 μM (as methylamine concentration in pore water was consistently below detection limit of approx. 5 μM), as previously used for measuring methylamine methanogenesis in Gulf of Cádiz MVs (Maignien et al., 2013). Therefore, methylamine methanogenesis rates should be considered as maximum potential rates assuming *in situ* concentration was the detection limit.

### Methanogen enrichment slurries

Cold-stored (4°C) sediment mini-cores from each MV were then further sub-sampled under aseptic conditions in an anaerobic chamber and used as inoculum for sediment slurry enrichments. Replicate slurries (50 ml) were made up using 25% (v/v) sediment (see Table 1 for sediment depths used) in artificial seawater (Köpke et al., 2005) contained in 100 ml serum bottles and supplemented with the substrates 12 mM sodium acetate, 1.5 mM benzoic acid, 4 mM hexadecane, 5 mM methanol, or 5 mM methylamine. Additionally, one set of sediment slurries were left without supplements as an unamended control and another set of slurries had their headspace gas replaced with H_2_/CO_2_ (80:20) as substrates. All slurries, except those with H_2_/CO_2_ had their headspace gas replaced with N_2_/CO_2_ and all methanogen enrichments were then incubated at 10°C in the dark for up to 170 days (cruise MSM1-3) or 300 days (cruise JC10).

At several time points during the incubation, samples of headspace gas (2 ml) were removed from all sediment slurries and analyzed by gas chromatography as above. For standardization, potential methanogenesis (methane production) rates were calculated from total methane produced after 130 days incubation (or in some samples after 300 days) with the methane production value from the relevant unamended control subtracted (32 out of 40 control slurries produced methane; Table 2).

**Table 2:** Methane production from Gulf of Cádiz mud volcano sediment slurries incubated for 130 days with a range of substrates.

Mud volcano	Station	Sediment sample depth (mbsf)		Methane production (nmol)^a^^,^^b^^,^^c^					
			Control^b^	Acetate	Benzoate	Hexadecane	Methanol	Methylamine	H_2_/CO_2_
Porto	144	0.77	++	−	++	+	+	++	−
		1.12	+	+	−	−	+	+	*
Bonjardim	131	0.42	+	−	−	−	+++	+++	−
		1.67	−	−	−	−	+++	−	−
		2.47	+	−	−	−	−	−	−
		2.97	+	+	*	+	−	*	*
Carlos Ribeiro	053	0.30	++	*	*	−	*	*	*
		0.55	++	*	*	*	* / +++	* /+++	*
		1.05	+	+	*	*	* / +++	+/ +++	*
		4.25	++	*	*	*	*	*	*
		5.15	+	−	+	−	+	−	+
Captain Arutyunov	191	0.52	++	*	−	−	+++	+++	++
		1.77	++	−	+	*	*	++	+
		2.67	−	−	−	−	−	−	−
	206	0.77	++	+	*	*	−	+++	*
		1.12	++	*	*	*	*	−	*
		2.22	++	−	*	+	+	++	*
		2.82	+	−	−	−	−	+	−
	227	0.77	+++	*	*	*	+++	+++	*
		1.77	++	*	*	*	+++	+++	*
	066	3.93	+++	*	*	*	*	*	*
Darwin	036	0.11	+	*	*	*	+++	+++	* / +++
	038	0.18	−	−	−	−	−	+ / +++	−
		0.48	−	−	−	−	−	−	−
Meknes	306	0.77	+++	++	++	++	+++	+++	−
		1.82	++	++	+	*	*	+++	*
Mercator	238	0.72	++	*	*	−	++	+++	−
		1.72	++	*	*	*	*	*	*
	002	0.05	−	−	−	−	−	−	−
		0.20	−	−	−	−	−	−	−
	009	0.13	++	++	++	++	*	*	*
	011	0.04	−	−	−	−	−	−	−
	013	0.15	+	++	++	++	++	+	−
	015	0.30	++	+	*	*	++	+	*
		0.48	−	−	−	−	−	−	−
		0.75	+	−	−	−	−	−	−
	019	0.18	++	++	*	*	*	*	*
		0.38	+	*	+	+	−	*	*
		1.90	++	++	++	++	+	*	*
		2.23	++	++	+	++	*	+	*

a Dark shaded boxes illustrate substrate amended slurries that have methane production >100 nmol CH_4_ after 130 d incubation. Light shaded boxes show slurries that developed high (>100 nmol) methane production after 300 d incubation. Pink colored boxes illustrate methane removal.

b Control slurries (no substrate added): No CH_4_ production (−) above 3x background (background = 3.3 nmol CH_4_); 10–100 nmol CH_4_ produced (+); 100–1,000 nmol CH_4_ produced (++); >1,000 nmol CH_4_ produced (+++).

c Substrate amended slurries: No CH_4_ production (−) above 3x background (background = 3.3 nmol CH_4_) after removal of control; 10–100 nmol CH_4_ produced (+) after removal of control; 100–1,000 nmol CH_4_ produced (++) after removal of control; >1,000 nmol CH_4_ produced (+++) after removal of control; >10 nmol CH_4_ consumed (*) after subtraction of control.

### DNA extraction of methanogen enrichments

Genomic DNA was extracted from methanogenic sediment slurry samples using the FastDNA® SPIN Kit for Soil (MP Biomedicals) as described (Webster et al., 2003). Essentially, 1 ml of sediment slurry was placed in a lysing matrix E tube (MP Biomedicals) and centrifuged at 15,000 × *g* for 1 min to pellet cells and sediment. Sediment pellets were then re-suspended in 800 μl of sodium phosphate buffer and 120 μl MT buffer (MP Biomedicals) before lysis in a FastPrep® 24 instrument (MP Biomedicals) for 2 X 30s at speed 5.5 ms^−1^. All remaining steps were as the manufacturer’s protocol, except that some spin and incubation times were extended (see Parkes et al., 2010). DNA was eluted in 100 μl molecular grade water (Severn Biotech) and stored at −80°C until required.

### 16S rRNA and mcrA gene PCR-DGGE analysis of methanogen enrichments

Bacterial and archaeal 16S rRNA genes were amplified by direct PCR from sediment slurry DNA extracts using DreamTaq DNA polymerase (Thermo Fisher Scientific) with primer pairs 357FGC-518R or SAfGC-PARCH519R, respectively and analyzed by DGGE (see Webster et al., 2006). Additionally, methyl-coenzyme M reductase genes (*mcrA*) were amplified by nested PCR using primers ME1f-ME2r and MLf-MLr without a GC-clamp and analyzed on 6–12% gradient (w/v) polyacrylamide DGGE gels with a 25–50% denaturant gradient (Webster et al., 2011). All DGGE gels were stained with SYBR Gold nucleic acid stain (Invitrogen), viewed under UV and images captured using a Gene Genius Bio Imaging System (Syngene). DGGE bands were excised, re-amplified by PCR, sequenced (O’Sullivan et al., 2008) and analyzed using the NCBI nucleotide BLAST program.^1^

### Methanogen isolation

A selected number of Gulf of Cádiz methanogen enrichments showing methane production were used as inoculum in attempts at isolating pure methanogens. Approximately 1 ml of enrichment culture was subcultured into 10 ml of artificial seawater (ASW) medium as described (Watkins et al., 2012) and supplemented with either 10 mM methylamine, 10 mM methanol or H_2_/CO_2_ (80,20, 0.1 MPa) as substrates. Growth was based on methane production monitored by gas chromatography. Methanogens were then isolated using a combination of deep-agar shake tubes and/or a dilution-to-extinction series with antibiotics to inhibit bacterial growth at 25°C (Parkes et al., 2010; Watkins et al., 2012). Methanogens were identified by PCR of the 16S rRNA and *mcrA* gene as described previously (Watkins et al., 2012).

### Archaeal and bacterial 16S rRNA gene sequencing of MV sediments

MV sediment samples stored for molecular analysis were used to conduct a microbial diversity survey using three different sequencing approaches. DNA was extracted from 5 g of each MV sample (Table 1) using the FastDNA® SPIN Kit for Soil (MP Biomedicals) as described (Webster et al., 2003). Bacterial (V1-V5 region) and archaeal (V2-V5 region) 16S rRNA genes were amplified by PCR from sediment samples using DreamTaq DNA polymerase (Thermo Fisher Scientific Inc.) with primers 27F-907R and 109F-958R, respectively, as described (Webster et al., 2006). Three replicate PCR products for each sample were cleaned, pooled and cloned in pGEM-T Easy vector (Promega) and transformed into *Escherichia coli* JM109 competent cells (Promega) according to manufacturer’s protocol. Recombinant colonies were picked, grown overnight at 37°C in 96-well plates containing LB liquid medium with 7.5% (v/v) glycerol and 100 μg ml^−1^ ampicillin, and the gene libraries stored at −80°C. Approximately 40–70 recombinant clones from each *Archaea* and *Bacteria* 16S rRNA gene library were amplified by PCR with M13 primers, checked and sequenced using an ABI 3130xl Genetic Analyzer (Applied Biosystems). Sequence chromatographs were analyzed using the Chromas Lite software package version 2.01,^2^ and gene sequences identified using nucleotide BLAST^3^ against the nucleotide collection and reference RNA sequence databases. New sequences reported here have been submitted to the NCBI database under accession numbers MT825700 - MT826197.

DNA from some MV samples was also amplified using methods of the International Census of Marine Microbes (ICoMM). The V6 region of the 16S rRNA gene from *Archaea* and *Bacteria* was amplified and subjected to 454 pyrosequencing on a GS20. All PCR methods, primers and analysis tools are detailed on the ICoMM website (https://vamps2.mbl.edu; Sogin et al., 2006). Clusters were generated using the single-linkage pre-clustering algorithm to smooth sequencing errors and reduce noise, followed by primary pairwise, average linkage clustering. OTUs were created using clustering thresholds of 3%, corresponding to 97% similarity (Huse et al., 2010). Tag sequences are publicly available from ICoMM as the datasets ICM_CFU_Av6 and ICM_CFU_Bv6.

Additionally, MV DNA samples were amplified using the universal 16S rRNA gene primers (V4 region) 515F-806R (Caporaso et al., 2011) and *Archaea*-specific primers 519F-958R (V4-V5). Amplicons for Illumina Miseq sequencing were made by dual indexed nested PCR (D’Amore et al., 2016) designed to amplify the 16S rRNA gene and incorporate the illumina adapters and sample identification barcodes as described in the Nextera DNA sample preparation guide (Illumina Inc.). All primers were synthesised by Integrated DNA Technologies, Inc. and PCR amplifications, purification, pooling and sequencing on an Illumina MiSeq platform were carried out by the Centre for Genomic Research (CGR), University of Liverpool. Raw Fastq files were trimmed for the presence of Illumina adapter sequences using Cutadapt version 1.2.1 (Martin, 2011) and for a minimum window quality score of 20 using Sickle version 1.200 (Joshi and Fass, 2011) by CGR who provided trimmed data suitable for downstream analysis. Overlapping regions within the paired-end Illumina sequencing reads were aligned to generate contigs and quality filtered using USEARCH (Edgar, 2010). Further analysis of sequencing data was performed in QIIME (Caporaso et al., 2010). Representative OTUs were picked with pick_de_novo_otus.py command in QIIME at 97% similarity and taxonomy assigned with the Greengenes database (DeSantis et al., 2006). Singletons, unidentified and non-specific sequences (e.g., *Bacteria* sequences in the *Archaea*-specific library) were removed.

## Results

### Sediment pore water geochemistry

**Figure 2:**
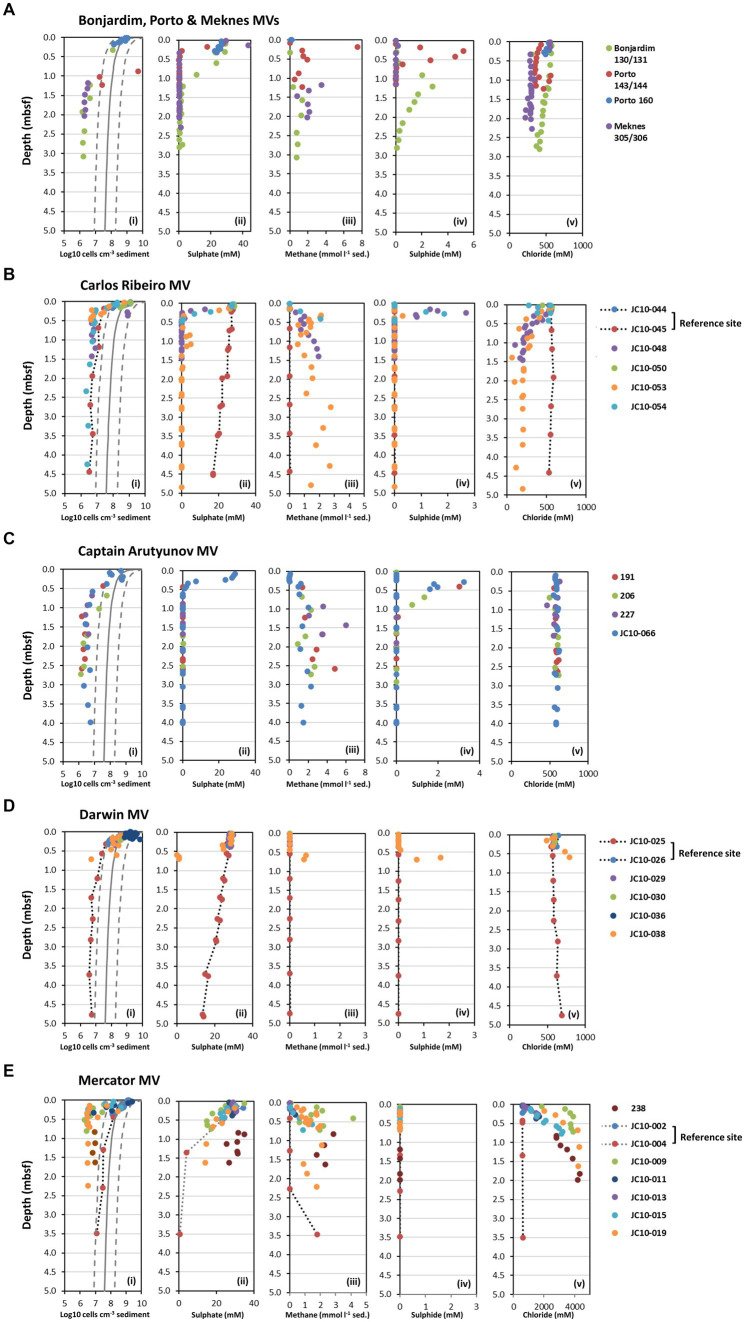
Depth profiles of prokaryotic cell numbers and geochemical data for seven mud volcanoes (MV) from the Gulf of Cádiz. **(A)** Depth profiles for Bonjardim, Porto and Meknes MVs, **(B)** Carlos Ribeiro MV, **(C)** Captain Arutyunov MV, **(D)** Darwin MV, and **(E)** Mercator MV. (i) Prokaryotic cell numbers determined by AODC. The solid line shows Parkes et al. (2000) general model for prokaryotic cell distribution in deep marine sediments, and dotted lines represent 95% prediction limits. Pore water concentrations of (ii) sulphate, (iii) *in situ* methane (iv), sulphide, and (v) chloride. Data shown for Bonjardim MV station 130, Porto MV station 143 and Meknes station 305 obtained from the PANGAEA Data Publisher for Earth and Environmental Science (https://www.pangaea.de/). mbsf, meters below seafloor.

Geochemical pore water profiles (methane, sulphate, sulphide, and chloride) from seven mud volcanoes in the Gulf of Cádiz sampled during two expeditions (MSM1-3 and JC10) are shown in Figure 2. All MV sites studied differed in their geochemical profiles. The majority of MV sediments had consistent or rapid sulphate removal with depth, demonstrating active prokaryotic sulphate reduction. Sulphate was completely removed in Bonjardim, Porto, Meknes, Carlos Ribeiro, Captain Arutyunov, and Darwin MVs between ~0.2 and 1.2 meters below seafloor (mbsf; Figure 2), whereas Mercator MV had sulphate concentrations of 14.0 mM (station 019) and 26.7 mM (station 238) at the bottom of the core for each station (1.63 mbsf; Figure 2E). For the majority of MV sediments with active sulphate removal, methane concentrations increased rapidly with depth reaching saturation at atmospheric pressure by 0.5 mbsf, indicating that active methanogenesis was occurring in the subsurface (with maximum concentrations of 7.5 and 6.0 mmol CH_4_ l^−1^ sediment at Porto MV station 143 and Captain Arutyunov MV station 227, respectively; Figure 2). Despite incomplete sulphate removal at Mercator MV, high methane concentrations (e.g., 4.2 mmol CH_4_ l^−1^ sediment at station 009) were detected throughout the sediment profile for all stations analyzed. Sulphide profiles at most MV sites peaked at the interface between the methane and sulphate gradients (Figure 2), indicative of a distinct sulphate methane transition zone (SMTZ) with active anaerobic oxidation of methane (AOM). The exception being Mercator MV which had no detectable sulphide (Figure 2E).

The methane and sulphate profiles at the Darwin and Carlos Ribeiro MV reference sites (stations 025 and 045, respectively) were very different to those of the MV sediments, with slower, linear sulphate removal (14.1 and 17.1 mM at ~4.5 mbsf), and very low CH_4_ concentrations in the subsurface (1–7 μmol CH_4_ l^−1^ sediment; Figure 2). Geochemical profiles at the Mercator MV reference sites 002 and 004 were similar to MV sediments with sulphate depletion and CH_4_ production in the subsurface, albeit at deeper depths (>2 mbsf). This may suggest that the Mercator MV reference sites are influenced by nearby MV activity, despite pore water salinity not being elevated. Discovery of a nearby buried mud volcano may also be influencing the observed biogeochemistry at this station (Perez-Garcia et al., 2011).

Mercator MV was clearly distinct from the other MV sites due to extremely high salinity at this site (Haffert et al., 2013) with chloride concentration in the surface sediments (e.g., stations 009 and 019) being 3.5-fold higher (2,011 mM) than typical sea water values (Figure 2E). Salinity further increased with depth to 10x seawater chloride values (5,585 mM) at 2.3 mbsf to almost halite saturation levels (~ 5,800 mM) as reported previously (Maignien et al., 2013). Carlos Ribeiro MV and Meknes MV had salinity values lower than that of seawater with chloride around 200 and 300 mM, respectively, at depth. All other MV (Bonjardim, Porto, Captain Arutyunov and Darwin) and reference sites had salinities close to seawater concentrations throughout their depth profiles (Figure 2).

### Prokaryotic cell numbers

At all sites, AODC cell numbers at the sediment surface (0.01 to 0.20 mbsf) were within the range observed previously (0.03–6.96 ×10^9^ cells cm^−3^ sediment) for subseafloor sediments (Parkes et al., 2000) after which they decreased rapidly to 1.16 to 8.10 ×10^6^ cells cm^−3^ sediment at 0.2–0.5 mbsf (Figure 2) corresponding with the approximate depth of the SMTZ (Figure 2). Cell numbers below the SMTZ (varying depth 0.2–0.5 mbsf) then fell below the expected lower limit of the global subseafloor sediment trend line (0.95–3.89 × 10^6^ cells cm^−3^ sediment for 0.5–4.0 mbsf), where they remained relatively constant to the bottom of the sediment core. Mercator MV reference station 004 and Porto MV station 144 were however, exceptions to this observation. Cell counts at Mercator MV station 004 followed the global trend in subseafloor sediments (Figure 2E), showing a steady decline with depth. In contrast, Porto MV station 144 had exceptionally high numbers which were outside of the upper expected limit for subseafloor sediments at 0.9 mbsf (5.11 × 10^9^ cm^−3^ sediment) and then cell numbers declined rapidly and were close to the lower expected limit (Figure 2A). Interestingly, the low cell counts for most MV sediments below the SMTZ (0.2–0.5 mbsf) were equivalent to cell numbers found below 100 mbsf in other subsurface sediments (Supplementary Figure S1) based on the global subseafloor sediment trend (Parkes et al., 2000). This may suggest that prokaryotic populations from these depths are derived from populations at much greater depths and have been transported upwards during episodic MV eruptions. Such low numbers of cells presumably impacted on the downstream analysis of MV samples, as the majority of sediment samples analyzed for DNA analysis were not readily amplifiable by direct PCR with either archaeal or bacterial 16S rRNA gene primers (Supplementary Table S1).

### Microbial diversity of gulf of Cádiz MV sediments

Microbial populations from the methanogenic zones of different Gulf of Cádiz MVs were investigated by a combination of molecular methods including 16S rRNA and *mcrA* gene clone libraries, 16S rRNA gene tag sequencing and illumina 16S rRNA gene amplicon sequencing. However, DNA was only successfully amplified by direct PCR from seven different sampling stations covering four different MV sites (Darwin, Bonjardim, Captain Arutyunov and Porto MVs; Supplementary Figures S2–S4). Bacterial and archaeal 16S rRNA gene libraries made by different methods were generally in agreement with the exception of the *Archaea* V4-V5 region 16S rRNA gene amplicon library (Supplementary Figure S2) which differed considerably in identification of major archaeal taxonomic groups when compared with the other methods used. This may be due to PCR bias introduced by this set of archaeal primers (Teske and Sørensen, 2008).

Archaeal diversity at all four MV sites analyzed (Supplementary Figure S2) showed that *Archaea* involved in methane cycling (methanogens and ANME) were present at each site by at least one PCR-sequencing method. However, their proportions varied between MV sites and between stations of the same MV. For example, ANME-1 phylotypes at Captain Arutyunov MV were prevalent but varied with station; 88.4% at station 066, 52% at station 206, 39.2% at station 227 and 14% at station 191 when comparing the V2-V5 region 16S rRNA gene clone libraries. Whereas methanogens from the *Euryarchaeota* orders *Methanosarcinales* and *Methanobacteriales* were dominant at station 191 (65% of V2-V5 region 16S rRNA gene library) but were a minor component or absent at the other three Captain Arutyunov MV stations (0–6% of V2-V5 region 16S rRNA gene libraries). A high proportion (67.3%) of ANME-1 sequences were also present at station 191 in the V4-V5 region 16S rRNA gene library. Other major taxa at Captain Arutyunov included members of the *Euryarchaeota* belonging to *Thermoprofundales* (also known as Marine Benthic Group-D or *Izemarchaea*; Baker et al., 2020), *Poseidonales* (formerly Marine Group II; Rinke et al., 2019), *Pontarchaea* (formerly Marine Group III; Adam et al., 2017), *Methanomicrobiales*, *Hadesarchaea* (formerly SAGMEG; Baker et al., 2016) and Terrestrial Miscellaneous Euryarchaeotal group (Takai et al., 2001), along with members of the *Bathyarchaeota* (formerly Miscellaneous Crenarchaeotal Group; Meng et al., 2014), *Lokiarchaeota* (formerly Marine Benthic Group-B or Deep-Sea Archaeal Group; Baker et al., 2020), *Parvarchaeota* and *Thaumarchaeota* (including Marine Group 1 and Marine Benthic Group-A; Baker et al., 2020; Supplementary Figure S2).

Similar groups of *Archaea* were also present at Darwin MV station 038 and Bonjardim MV station 131; with Bonjardim MV having a high proportion of 16S rRNA gene sequences belonging to *Methanosarcinales* and *Lokiarchaeota*, and Darwin MV having a high number of ANME-1 and *Bathyarchaeota* 16S rRNA gene sequences, as well as *Lokiarchaeota* identified by V4-V5 16S rRNA gene amplicon sequencing. In contrast, Porto MV at 0.9 mbsf was dominated almost entirely by members of the ANME-1 with 96.8–100% of archaeal 16S rRNA gene sequences identified by all PCR-sequencing methods. Methanogens and ANME were also confirmed in all four MV sediments successfully analyzed for *mcrA* genes. ANME-1 and ANME-2 *mcrA* gene sequences were dominant in all MVs with the exception of Bonjardim MV which had 100% of *mcrA* gene sequences belonging to the methanogen genus *Methanococcoides* (Supplementary Figure S3).

Overall, bacterial diversity (Supplementary Figure S4) was higher than archaeal diversity with a greater average number of higher taxonomic groupings being identified per MV sediment analyzed (14 bacterial groups compared with 9 archaeal groups). However, all MVs were dominated or had a high proportion of *Atribacterota* (class JS1; Webster et al., 2004; Nobu et al., 2016), *Chloroflexota*, *Pseudomonadota* (formerly *Proteobacteria*), *Deltaproteobacteria*, *Planctomycetota*, *Bacillota* (formerly *Firmicutes*) and *Ca.* “*Aminicenantes*” (formerly OP8; Farag et al., 2014) with other bacterial lineages being less abundant (Supplementary Figure S4). Interestingly, Porto MV station 144 (0.9 mbsf) and Captain Arutyunov MV station 206 (0.7 mbsf) had up to 94 and 73% *Atribacterota* (class JS1) 16S rRNA gene sequences, respectively.

### Methanogenic activity *ex situ*

Methanogenesis from four different ^14^C-substrates (acetate, bicarbonate, methylamine and methanol) was detected at all MV sites (Carlos Ribeiro, Captain Arutyunov, Darwin and Mercator) analyzed, albeit at low rates (0.001–50.5 pmol cm^−3^ d^−1^) and at discrete sediment depths (Supplementary Figure S5; Supplementary Table S5). However, despite these low rates, the average rate of methanogenesis for each MV was elevated when compared to the respective non-mud volcano reference site, with average MV methanogenic rates being up to two orders of magnitude higher (Supplementary Table S2). Maximum rates of methanogenesis tended to occur in the upper 0.5 m of MV sediments (Supplementary Figure S5) and at some sites this corresponded with decreases in sulphate concentrations (e.g., Carlos Ribeiro and Captain Arutyunov; Figure 2). Interestingly, the average proportion of combined methanogenic rates for the four MV sites due to hydrogenotrophic and acetoclastic methanogenesis (Supplementary Table S2) were similar at 22.8 and 23.7%, respectively, with methylotrophic rates using methylamine and methanol accounting for the remainder (48.9 and 4.6%, respectively). However, methylotrophic methanogenesis was less dominant in the reference station sediments, accounting for 35.9% with methylamine (and 0% from methanol) of the combined average methanogenic rates, with acetate and H_2_/CO_2_ being responsible for the remainder (33.3 and 30.8%, respectively; Supplementary Table S2).

### Methanogenesis from different substrates in MV sediment slurries

Methanogenesis from added substrates (Table 2) was detected in 68 (28.3%) of the MV sediment slurries (total 240 sediment slurries) incubated for 130 days, with 50% of the methane producing slurry enrichments having been supplemented with the methylotrophic substrates, methanol and methylamine (15 and 19 enrichments, respectively). Acetoclastic methanogenesis represented 17.7% of the methanogenic slurries, while only 4.4% of the slurries produced methane with H_2_/CO_2_. The remaining methane producing slurries were supplemented with either benzoate (14.7%) or hexadecane (13.2%). An additional subset of sediment slurries (40 control sediment slurries) without substrates also showed methane production ranging from no detection to >1,000 nmol CH_4_ (see Table 2), presumably due to methane degassing and/or methane production from *in situ* substrates, confirming the above methanogenesis activity measurements. This control methane value was then removed from the parallel amended slurries.

**Figure 3:**
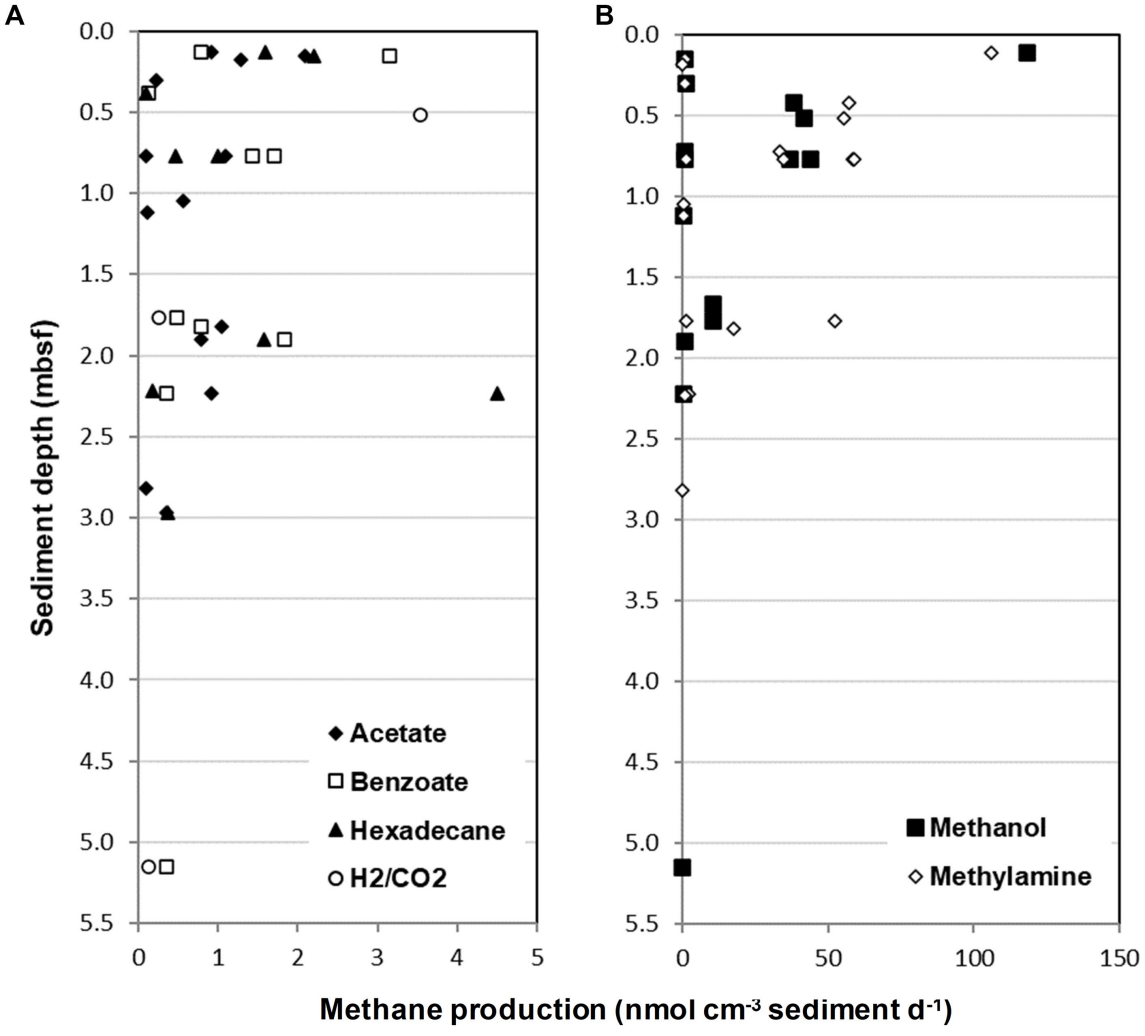
Methane production from Gulf of Cádiz mud volcano sediment slurries incubated for 130 days with a range of methanogenic substrates. **(A)** Methane produced from sediment slurries incubated with either acetate, benzoate, hexadecane or H_2_/CO_2_ plotted against sediment sample depth. **(B)** Methane produced from sediment slurries incubated with either methanol or methylamine. Only methane producing sediment slurries are shown (see Table 2 for further information). mbsf, meters below seafloor.

Closer inspection of the sediment slurries taking into account MV and sediment depth (Table 2) demonstrated that 55% of the different sediments tested (40 different sediments) had the potential to produce methane from methylamine or methanol. Whereas methane from other substrates was only found in 30 and 7.5% of sediments with acetate and H_2_/CO_2_, and 25 and 22.5% of sediments for benzoate and hexadecane, respectively. Interestingly, of all the different metabolic groups of methanogens tested for, only methylotrophic methanogenesis occurred at all seven MV sites across the Gulf of Cádiz. However, despite methylotrophic methanogenesis being prevalent in the Gulf of Cádiz MV sediments, methane production varied considerably depending on MV site and/or sediment depth. For example, methane produced ranged from 0.11–105.91 nmol CH_4_ cm^−3^ d^−1^ for methylamine and 0.08–118.24 nmol CH_4_ cm^−3^ d^−1^ for methanol, with highest methane production for both substrates being in sediments from Darwin MV at 0.11 mbsf (Figure 3; Table 2). Generally, rates of methylotrophic methanogenesis were highest in near surface sediments and decreased steadily with sediment depth at all MV sites (Figure 3B).

Similarly, potential methanogenesis also varied between MV sites and sediment depths for all other substrates tested, but methane production was often several orders of magnitude lower than with methylamine or methanol (Figure 3; Table 2). For example, methane produced by acetoclastic methanogenesis varied from 0.09–2.09 nmol CH_4_ cm^−3^ d^−1^, hydrogenotrophic methanogenesis ranged from 0.12–3.54 nmol CH_4_ cm^−3^ d^−1^ and hexadecane and benzoate were 0.1–4.51 and 0.12–3.16 nmol CH_4_ cm^−3^ d^−1^, respectively (Figure 3A; Table 2). Interestingly, the highest methane produced for these substrates occurred at different MVs to that with methylamine and methanol, with acetate, benzoate and hexadecane showing the highest rates of methanogenesis at Mercator MV, and H_2_/CO_2_ at Captain Arutyunov MV. Also, in contrast to methylotrophic methanogenesis, methane production for acetate, benzoate, hexadecane, and H_2_/CO_2_ did not show a clear trend with sediment depth (Figure 3A).

### Methanogenesis in substrate amended slurries from different MVs

At the deep water (3,860 m) Porto MV, 58.3% of sediment slurry enrichments produced methane with 16.6% of these showing methane production higher than 100 nmol CH_4_ after 130 days, whereas at the other two deep water (>2,000 m) sites (Bonjardim and Carlos Ribeiro MV), the numbers of positive methanogenic slurries were lower, with 20.8% at Bonjardim MV (12.5% with >100 nmol CH_4_ after 130 days) and 16.6% at Carlos Ribeiro MV (0% with >100 nmol CH_4_ after 130 days). The proportion of sediment slurries from the intermediate water depth (~1,000 m) MV sites, Captain Arutyunov MV and Darwin MV, producing methane were 26.7 and 16.6%, respectively, with a similar low percentage of these methanogenic slurries (16.7 and 11.1%, respectively) having methane production values >100 nmol CH_4_ after 130 days. In contrast, the shallow Meknes MV site (694 m) showed both a high percentage of positive methane producing slurries (66.6%) and a high number (58.3%) of methanogenic slurries with methane production >100 nmol CH_4_ after 130 days. However, Mercator MV, the shallowest MV site (346–470 m) which also had the largest number of sediments sampled (14 different sediments) for methanogenesis only had 28.6% positive methanogenic slurries, and 19% of these were >100 nmol CH_4_ after 130 days. Such contrasting results suggest that the potential for MVs to produce methane is not linked with their seawater depth.

However, the sediment slurries that produced the highest amounts of methane (>100 nmol CH_4_ after 130 days) from MVs with a water depth > 1,000 m (Porto, Bonjardim, Captain Arutyunov and Darwin MVs) were dominated by methylotrophic methanogenesis (15 out of 17 high-rate slurries; Table 2). Whereas MVs located below 1,000 m water depth (Meknes and Mercator MVs) were capable of producing high amounts of methane from a much broader range of metabolic substrates. For example, sediments from Meknes and Mercator MVs had only 7 out of 23 high-rate methanogenic slurries with added methylamine or methanol (Table 2).

Furthermore, increased incubation times (up to 300 days) led to several MV sediment slurries, which at 130 days showed no or low rates of methanogenesis (<10 nmol CH_4_ after 130 days), developing the ability to generate high amounts of methane (>100 nmol CH_4_ after 300 days). These were all from sediments of Carlos Ribeiro MV (0.55 and 1.05 mbsf) and Darwin MV (0.11 and 0.18 mbsf; Table 2), and the majority (83.3%) of these extended incubation time high-rate methanogenic slurries were incubated with the methanogenic substrates, methanol or methylamine.

### Methane consumption in MV sediment slurries

Methane removal was also observed in a number of sediment slurries incubated with methanogenic substrates (Table 2) presumably due to anaerobic oxidation of methane (AOM). Interestingly, the number of slurries with detectable AOM (defined as >10 nmol CH_4_ consumed after subtraction of unamended control slurry) were similar for each substrate tested (10–16 slurries per substrate) with the exception of H_2_/CO_2_ which accounted for 24.7% (21 slurries) of AOM slurries. The highest number of sediment slurries detected with AOM were obtained from sediments from Captain Arutyunov, Mercator and Carlos Ribeiro MVs with 27, 26 and 21 AOM slurries, respectively. Furthermore, Carlos Ribeiro MV sediments were particularly effective at removing methane as this MV had 70% (21 out of 30) of its sediment slurries showing AOM.

### Methanogen diversity in MV sediment methanogenic slurries

**Figure 4:**
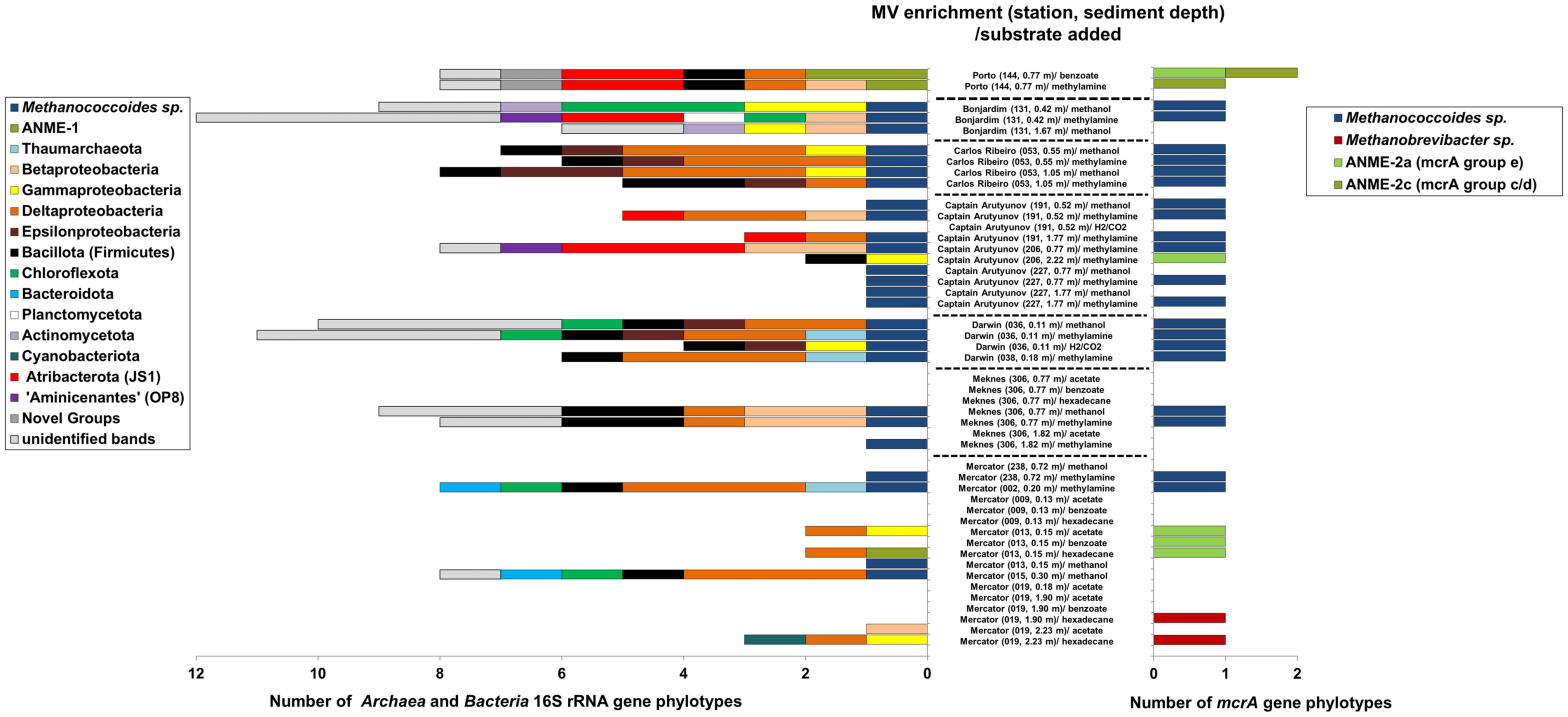
Archaeal and bacterial diversity in methane producing Gulf of Cádiz mud volcano sediment slurries incubated with a range of methanogenic substrates. Diversity was assessed by PCR-DGGE analysis of 16S rRNA and *mcrA* genes after 130 d incubation. Unidentified bands refer to faint bands that were not excised and sequenced.

All MV sediment slurries showing relatively high rates of methanogenesis (>100 nmol CH_4_) after 130 days incubation were screened for archaeal 16S rRNA and methanogen-specific *mcrA* genes using PCR-DGGE and sequencing (Figure 4). Despite a range of substrates shown to produce methane (Table 2), the majority of successful 16S rRNA gene PCR amplifications from extracted DNA were mainly obtained from sediments incubated with either methanol or methylamine. Presumably due to the potential rates of methanogenesis in these methylotrophic slurries often being 10-100-fold higher (Figure 3; Table 2) than those from other substrates, resulting in a stimulation of methanogen cell numbers and increased biomass and/or extractable DNA.

Sequencing excised archaeal 16S rRNA gene DGGE bands revealed that most of the methanogenic enrichments incubated with either methanol or methylamine were dominated by *Archaea* belonging to the methanogen order *Methanosarcinales* and were related (92–100% sequence similarity) to sequences previously found in a number of mud volcanoes and marine sediments (Supplementary Table S3), as well as pure cultures of *Methanococcoides alaskense* (97–99%) and *Methanococcoides methylutens* (99–100%). However, one methanogenic sediment slurry (Figure 4) from Porto MV (0.77 m) and incubated with methylamine did not contain detectable methanogens, but surprisingly was dominated by anaerobic methane-oxidizing *Archaea* ANME-1. These sequences were related (98% sequence similarity) to 16S rRNA gene sequences previously found in sulphate–methane transition zone (SMTZ) sediments from Aarhus Bay (Webster et al., 2011). Similar ANME-1 sequences were also found in Porto MV sediment enrichments incubated with benzoate (Figure 4), along with a second ANME-1 16S rRNA gene phylotype related to sequences from a mud volcano (Amsterdam MV) in the Eastern Mediterranean Sea (Pachiadaki et al., 2011). ANME-1 sequences were also the dominant archaeal group in the methanogenic slurry from Mercator MV near surface sediment (0.15 mbsf) incubated with hexadecane (Supplementary Table S3). However, it should be noted that the presence of ANME-1 within the enrichments could be due to their persistence in the sediment slurry rather than their use of the methanogen substrates directly.

Methanogen diversity in all methanogenic slurries were also investigated using the *mcrA* gene. The majority of methylotrophic sediment slurries analyzed by PCR-DGGE resulted in congruent *mcrA* gene sequences to those found by 16S rRNA gene analysis (Figure 4). Many slurries contained *mcrA* gene sequences that were 96–100% similar to those found in several *Methanococcoides* species and shared 97–99% sequence similarity to sequences previously found in marine sediments from Guaymas Basin, Marennes-Oléron Bay and Cascadia Margin (Dhillon et al., 2005; Roussel et al., 2009; Yoshioka et al., 2010). However, no *mcrA* gene sequences were found that corresponded with ANME-1 archaea, instead all ANME-related *mcrA* sequences identified in Gulf of Cádiz MV sediment slurries were related to sequences that grouped with ANME-2 (Supplementary Table S3). For example, Porto MV (0.77 mbsf) methanogenic sediment slurries incubated with benzoate or methylamine contained ANME-2a (*mcrA* group e) and/or ANME-2c (*mcrA* groupc/d) *mcrA* sequences similar (96–100% sequence similarity) to those found in sediments from Napoli MV (Lazar et al., 2011a) and Eel River Basin (Beal et al., 2009). Interestingly, methanogenic slurries from Mercator MV site 019 at depths 1.90 and 2.23 msf incubated with hexadecane contained *mcrA* gene sequences that were closely (100% sequence similarity) related to the hydrogenotrophic methanogen *Methanobrevibacter arboriphilus*.

### Bacterial 16S rRNA gene diversity in MV sediment methanogenic slurries

In contrast to Archaea, the bacterial diversity within many of the methanogenic slurries was relatively high (Figure 4) with slurries containing sequences representative of several phyla (sometimes up to 5 phyla per slurry) and up to 11 detectable bacterial phylotypes (e.g., Bonjardim MV). Overall bacterial phylotypes belonged to 13 major taxa, including members of the *Betaproteobacteria*, *Gammaproteobacteria*, *Deltaproteobacteria*, *Campylobacterota* class *Epsilonproteobacteria*, *Bacillota*, *Chloroflexota*, *Bacteroidota*, *Planctomycetota*, *Actinomycetota*, *Cyanobacteria*, *Atribacterota* (JS1) and *Ca.* “*Aminicenantes*” (OP8), and one novel phylum level group previously found in deep marine sediments of the Japan Sea (Yanagawa et al., 2011). However, despite high bacterial diversity within the sediment slurries no clear pattern was observed to suggest that bacterial diversity was related to the methanogenic substrates added. Interestingly, sequences from *Atribacterota*, *Ca.* “*Aminicenantes*,” *Actinomycetota*, *Planctomycetota* and the novel bacterial group were only found in sediment slurries from MVs located in water depths >1,000 m, whereas *Pseudomonadota* and *Bacillota* were found in most MV sediments. It should also be noted that several methanogenic slurries dominated by *Methanococcoides* species contained no detectable *Bacteria* (e.g., Captain Arutyunov MV), and that many of the bacterial phylotypes that were detected in slurries were related to sequences previously found in other marine sediments (Supplementary Table S4), including those from cold seeps and submarine mud volcanoes (e.g., Heijs et al., 2008; Orcutt et al., 2010; Pachiadaki et al., 2011; Yanagawa et al., 2011).

### Isolation of methanogens

MV sediment slurries (from Bonjardim, Carlos Ribeiro, Captain Arutyunov, Darwin, Meknes and Mercator) showing high methanogenic activity and detectable methanogens, by both 16S rRNA and *mcrA* genes, were used to isolate novel methanogens. After further enrichment, subculture and isolation (see Watkins et al., 2012, 2014) five out of six MV sediments yielded pure methanogen cultures. The majority of pure methanogens were able to utilise methylotrophic substrates and one was isolated on H_2_/CO_2_. All isolated methanogens belonged to the methylotrophic genus *Methanococcoides* (Table 3), with the exception of the one hydrogenotrophic *Methanogenium* species from the surface sediments of Darwin MV.

**Table 3:** Pure methanogen cultures isolated from Gulf of Cádiz mud volcano methanogen sediment slurries.

Mud volcano	Station	Sediment depth (mbsf)	Substrate	Methanogen
Bonjardim^a^	131	0.42	Methanol	–
	131	0.42	Methylamine	–
Carlos Ribeiro	053	0.55	Methylamine	*Methanococcoides* sp. CRMV-M
	053	0.55	Methanol	*Methanococcoides* sp. CRMV-MeOH
Captain Arutyunov	206	0.77	Methylamine	*Methanococcoides* sp. CAMV-M
Darwin	036	0.11	H_2_/CO_2_	*Methanogenium* sp. DM-H
	036	0.11	Methylamine	*Methanococcoides* sp. DM1^b^
	036	0.11	Methanol	*Methanococcoides* sp. DMV-MeOH
	038	0.18	Methylamine	*Methanococcoides* sp. DMVREF-M
Meknes	306	0.77	Methylamine	*Methanococcoides* sp. MKM1^b^
	306	0.77	Methanol	*Methanococcoides* sp. MKMV-Me0H
Mercator	002	0.20	Methylamine	*Methanococcoides* sp. MMVREF-M

mbsf, meters below seafloor.

a All subcultures from the Bonjardim MV methanogen sediment slurry did not produce methane after 168 d of incubation and as a result no methanogens were isolated from this slurry.

b *Methanococcoides* species isolated from these enrichments have been characterized further (see Watkins et al., 2012, 2014).

## Discussion

The Gulf of Cádiz is a tectonically active region of the European continental margin characterized by a high number of submarine mud volcanoes (Figure 1) that have been frequently studied since their discovery in 1999 (Kenyon et al., 2000; Pinheiro et al., 2003; Niemann et al., 2006a; Medialdea et al., 2009; Maignien et al., 2013; Nuzzo et al., 2019). Research findings at many of these MVs indicate past and present methane seepage, presence of methane hydrate-bearing sediments, authigenic carbonates and seep-related macrofauna (Gardner, 2001; Díaz-del-Rio et al., 2003; Pinheiro et al., 2003; Cunha et al., 2013). However, despite these studies and the extensive interest in mud volcanoes from the Gulf of Cádiz, there is relatively little information on methanogenesis, and the prokaryotic populations involved.

### Mud volcano microbial diversity

Our microbial diversity survey of mud volcanoes from the Gulf of Cádiz showed that sediments from below the SMTZ and within the methanogenic zone (below approx. 0.3 mbsf) have generally low numbers of prokaryotic cells, except Porto MV at 0.9 mbsf which had exceptionally high numbers (Supplementary Figures S6). Based on 16S rRNA gene sequences, microbial populations were dominated by methanogenic archaea (*Methanosarcinales*), anaerobic methane-oxidizing archaea (ANME), *Bathyarchaeota*, *Lokiarchaeaota* and bacteria such as, *Atribacterota*, *Chloroflexota*, *Bacillota* and *Deltaproteobacteria* (Supplementary Figures S2, S4, and S6) frequently found in other methane-rich sediments (Inagaki et al., 2006; Parkes et al., 2014; Vigneron et al., 2015; Katayama et al., 2016; Teske et al., 2021). Similar groups of archaea and bacteria have also been identified at submarine MVs from other locations including Håkon Mosby, Amsterdam, Napoli, Kazan, Chefren, Ryukyu Trench and Beaufort Sea MVs (Niemann et al., 2006b; Heijs et al., 2007; Omoregie et al., 2008; Lazar et al., 2011b, 2012; Pachiadaki and Kormas, 2013; Hoshino et al., 2017; Lee et al., 2019) and from the Gulf of Cádiz (Captain Arutyunov MV, Niemann et al., 2006a; Mercator MV, Maignien et al., 2013). For example, ANME-1 and ANME-2 were found to be the dominant archaea groups in sediments below the SMTZ (0.15 mbsf) at Amsterdam MV, ranging between 74 and 91% of the sequences found with only a few recognized methanogen groups (4% of sequences), whereas *Deltaproteobacteria* and *Atribacterota* accounted for 34 and 24%, respectively of the bacterial population (Pachiadaki et al., 2011). Similarly, ANME-1 and ANME-2 phylotypes dominated the sediments (0.4 mbsf) at Captain Arutyunov MV with members of the *Deltaproteobacteria* SEEP SRB-1 group (the AOM syntrophic partners of ANME-1 and ANME-2) accounting for 85% of bacterial sequences with a further 6% of sequences belonging to *Atribacterota* (Niemann et al., 2006a). Interestingly, in the study by Niemann et al. (2006a) a novel *Bacillota* clone CAMV300B902 was also identified, and similar 16S rRNA gene sequences were present in our study of Captain Arutyunov MV site 191 (0.45 mbsf) at high frequency (up to 55% in the V1-V5 16S rRNA gene library). Thus far, closely related sequences (>99% sequence similarity) to this phylotype have only been found at Captain Arutyunov and Kazan MVs (Niemann et al., 2006a; Pachiadaki et al., 2010), and their function is unknown. However, of note is that the phylotype’s nearest pure culture relative (90% 16S rRNA gene sequence similarity) is the SRB, *Desulforudis audaxviator* (Karnachuk et al., 2019), found in a deep South African gold mine, where it formed a near single-species ecosystem fueled by H_2_ from water radiolysis (Chivian et al., 2008). This may suggest that novel *Bacillota* involved in hydrogen metabolism could be important bacteria at specific mud volcanoes. However, further investigations are necessary to determine the role of these bacteria.

Methanogens and methanotrophic archaea were also evident from analysis of the gene encoding for methyl coenzyme M reductase (*mcrA*), an enzyme which catalyzes the final step of the methanogenic pathway present in all methanogens and anaerobic methanotrophs (Hallam et al., 2003). The *mcrA* gene libraries were dominated by sequences belonging to ANME, with three (Darwin, Captain Arutyunov and Porto MV) out of the four mud volcanoes analyzed having 89–100% of *mcrA* gene sequences belonging to ANME-1 or ANME-2 lineages. Interestingly, only the gene library from Bonjardim MV was clearly dominated by methanogen *mcrA* genes, with 100% of sequences being related (93–98% sequence similarity) to *mcrA* genes found previously in gas hydrate sediments of the Cascadia Margin (Expedition IODP 1327; Yoshioka et al., 2010) and known cultured *Methanococcoides* species (Webster et al., 2019; Supplementary Figure S3). Similar *Methanococcoides*-related *mcrA* sequences were also present at Captain Arutyunov MV stations 206 and 191 but at much lower abundance, comprising 3–7% of the respective gene libraries. In support of the *mcrA* gene findings, low numbers of 16S rRNA gene sequences of *Methanococcoides* species were also identified in all Gulf of Cádiz MV sites analyzed here and ranged from 25% at Bonjardim MV to 0.01% at Porto MV (Supplementary Figure S2), and 1.2% in a separate study at Mercator MV (Maignien et al., 2013). In addition, other novel members of the *Methanosarcinales* were also identified that were related to 16S rRNA gene sequences from sediments of the Kazan MV (Heijs et al., 2007; Pachiadaki et al., 2010) and the terrestrial Lei-Gong-Huo MV (Chang et al., 2012).

### Methanogenesis in mud volcano sediments

Whilst measurements of methanogenesis in global marine sediments are relatively widespread there is comparatively little information from on or around mud volcanoes. Krüger et al. (2005) measured methanogenesis and methane oxidation at a wide variety of ocean sites, including the Håkon Mosby MV, using both *in vitro* and ^14^C-radiotracer techniques. This study found that methanogenic rates at Håkon Mosby MV were in the range of those found at other marine sediment sites, with 0.02–0.08 μmol CH_4_ g^−1^ sediment d^−1^ (Krüger et al., 2005). However, these rates of methanogenesis were considerably higher than those found in our study of MV sediments of the Gulf of Cádiz (Supplementary Table S2; Supplementary Figure S5) and that by Maignien et al. (2013). Interestingly, an earlier study of the Håkon Mosby MV demonstrated that there was substantial variation (up to 4 orders of magnitude) in methanogenesis rates depending on where samples were located within the MV crater (Pimenov et al., 1999), with some rates being comparable to those measured at Gulf of Cádiz MVs. Clear differences in methanogenesis due to sample location were also observed in our study, demonstrating a high degree of variation between sample location at each MV site (Supplementary Figure S5). For example, Mercator MV had a peak rate of hydrogenotrophic methanogenesis of 50.54 pmol cm^−3^ d^−1^ at station 013 located within the crater rim but no activity was detected at any other station, including sediment cores from the crater center (stations 009 and 019). Furthermore, low rates of methanogenesis were also reported for the submarine mud volcano KMV#5 in the Nankai accretionary complex where hydrogenotrophic and acetoclastic methanogenesis were 0.6–128 and 0.004–0.10 pmol cm^−3^ d^−1^, respectively (Ijiri et al., 2018). But unlike KMV#5 where hydrogen was the dominant substrate for methanogenesis, it appears that methylotrophic methanogenesis is the dominant pathway with nearly 50% of methanogenesis across all MV sites at the Gulf of Cádiz using methylamine (Supplementary Table S2).

### Methylotrophic methanogenesis in MV sediment enrichments

Despite the low rates of methanogenesis measured in this study we were able to enrich methanogens and measure potential rates of methanogenesis *in vitro* from a number of mud volcanoes and sediment depths using a range of different substrates. These sediment enrichments further confirm that MV sediments from the Gulf of Cádiz are able to carry out methanogenesis and that the majority of MVs are dominated by methylotrophic methanogenesis rather than hydrogenotrophic or acetoclastic methanogenesis.

All methane producing sediment slurries incubated with methylamine or methanol were dominated by *Methanococcoides*-related methanogens. These methanogens are obligate methylotrophs that utilise C1 compounds as methanogenic substrates (Liu and Whitman, 2008). Unlike acetoclastic and CO_2_-reducing methanogens, methylotrophic methanogens are not outcompeted by SRB in sulphate-rich marine sediments for substrates (Purdy et al., 2003), enabling them to undertake methanogenesis in shallower anoxic sediments. In turn, this allows them to take advantage of methylated compounds (e.g., methanol and methylamines) that are typically released during the decay of organic osmolytes from marine organisms, including both prokaryotic and eukaryotic organisms present in marine sediments (Jones et al., 2019). In this study all positive methylotrophic sediment slurries were from the first 2 mbsf sediments, suggesting that *in situ* populations of *Methanococcoides* are abundant in shallow subsurface sediments of mud volcanoes in the Gulf of Cádiz, presumably where methylotrophic (non-competitive) substrates are available, and that methylotrophic methanogens are not outcompeted by SRB.

In addition, as sediment depths increased, potential *in vitro* rates of methylotrophic methanogenesis generally decreased, which may reflect decreasing numbers of viable *Methanococcoides* cells. For example, in coastal marine sediments it has been shown by quantitative real-time PCR that *Methanococcoides* 16S rRNA genes can represent up to 20% of the near surface (~10 cmbsf) archaeal population, and then decline to >1% in deeper sediments (Webster et al., 2015), similar to the *Methanococcoides* abundance values detected in 16S rRNA gene libraries in this study. Furthermore, Methanococcoides species were abundant in South China Sea sediments down to ~8.3 mbsf (Xu et al., 2021). Methanol and methylamines have been shown to be important substrates for methanogens in salt marsh sediments (Oremland et al., 1982), mangroves (Lyimo et al., 2009) and marine sediments (King et al., 1983; Zhuang et al., 2018; Xu et al., 2021), plus methylamines are the main methanogenic substrates in MV sediments from the Eastern Mediterranean Sea (Lazar et al., 2011b, 2012). Some *Methanococcoides* species isolated from the Gulf of Cádiz (Table 3) and the Napoli MV have also been shown to be capable of utilizing tri-, di and mono-methylamine and methanol, as well as other methylotrophic substrates like choline, dimethylethanolamine and glycine betaine directly (Watkins et al., 2012, 2014; L’Haridon et al., 2014). Studies of coastal salt marsh sediments have shown that closely related *Methanococcoides* species also undertake syntrophic partnerships with bacteria in a two-step process involving the formation of trimethylamine from glycine betaine (Jones et al., 2019) or choline (Jameson et al., 2019) to produce methane. It is conceivable that similar syntrophic relationships could also be occurring in MV sediments when more complex methylotrophic substrates are available, especially as glycine betaine-degrading bacteria are thought to be widespread in subsurface sediments (Jones et al., 2019). In the Gulf of Cádiz, sources of such compounds (methylamines, choline, and glycine betaine) could be made available to methanogens from the large populations of chemosynthetic macrofaunal communities that are often found around the crater of these MVs (Rodrigues et al., 2010; Cunha et al., 2013), as well as from the burial of microbial biofilms at the MV surface (Magalhães et al., 2012). Furthermore, detectable levels of methanol have also been found in sediments of several MVs including Captain Arutyunov and Carlos Ribeiro at depth (Supplementary Figure S7) as well as other subsurface sediments (Yanagawa et al., 2016; Zhuang et al., 2018; Xu et al., 2021), and methylamine has been shown to reversibly adsorb to clay minerals in sediments making it difficult to detect but remain biologically available to methylotrophic methanogens (Xiao et al., 2022). It maybe that such methanogens and methylotrophic methanogenesis is more widespread than previously thought (Valentine, 2011; Xu et al., 2021; Bueno de Mesquita et al., 2023) and that they are well suited to adapt to fluctuating geochemical environments, because of their ability to use a range of methylotrophic substrates. Furthermore, novel, and putative methylotrophic methanogens may also exist in the phyla *Bathyarchaeota* (Evans et al., 2015) and *Verstraetearchaeota* (Vanwonterghem et al., 2016), as well as the *Euryarchaeota* order *Methanomassiliicoccales* (Borrel et al., 2014), and may occur by ANME archaea (Bertram et al., 2013); all members of these archaeal groups can be identified in mud volcano sediments (Supplementary Figure S2). However, confirmation of methylotrophic methanogenesis within these groups can only be addressed by further investigations into the metagenomics or focused cultivation studies of MV sediments.

### Methanogenesis from acetate, hydrogen and other substrates in MV sediment enrichments

Hydrogenotrophic and acetoclastic methanogenesis activities occurred throughout sediments of the Gulf of Cádiz MVs, but potential rates were low (Supplementary Table S2). No methanogens were identified in any sediment slurries incubated with acetate or H_2_/CO_2_, and only six slurries (five with acetate) incubated with these two important methanogenic substrates produced methane that were > 100 nmol CH_4_ after 130 days incubation (Table 2). This is in contrast to the generally accepted idea that methanogenesis in marine sediments is dominated by CO_2_ reduction (Whiticar et al., 1986) and that acetate is an important substrate in deep subsurface sediments (Wellsbury et al., 2002). Hydrogenotrophic methanogenesis was reported to provide the majority of biogenic methane in the mud volcano KMV#5 from the Nankai accretionary complex, with active methanogenesis down to ~120 mbsf (Ijiri et al., 2018). Whereas, at the Amsterdam MV in the Eastern Mediterranean Sea, acetoclastic methanogenesis occurred, and was supported by high concentrations of acetate (up to 2 mM) in deeper sediment layers (Lazar et al., 2012). Similarly, methanogenic rates and *mcrA* gene sequences from an active brine seep mud volcano in the Gulf of Mexico revealed a predominance of acetoclastic over hydrogenotrophic methanogenesis (Joye et al., 2009). However, it maybe that longer incubations times (up to 2 years) are needed for batch enrichments of methanogens using these substrates (Katayama et al., 2022) or that more sophisticated methods similar to continuous-flow cultivation system used by Imachi et al. (2011) are necessary to culture methanogens from subseafloor sediments.

Methane production from hexadecane (Table 2), a long-chain alkane, in some methanogenic slurries (e.g., Mercator MV) demonstrated that methanogenesis from saturated hydrocarbons might be significant in MV sediments as previously shown in other anoxic environments (Zengler et al., 1999; Fowler et al., 2016). Methane from hexadecane often occurs by acetogenic bacteria decomposing alkanes to acetate and H_2_, which in turn are available substrates for acetoclastic and hydrogenotrophic methanogens (Dolfing et al., 2008). In our study no direct evidence for such bacteria was observed in any hexadecane enrichment, although sequences related to acetogens (*Acidaminobacter* species; Stams and Hansen, 1984) were identified in other substrate-amended enrichments from the same MV sediment. However, syntrophic associations involving hydrogenotrophic methanogens and other bacteria, including SRB that can degrade hexadecane to CO_2_ are also known (Brennan and Sanford, 2002). Intriguingly, *Deltaproteobacteria* 16S rRNA gene sequences related to SRB (Supplementary Table S4) and hydrogenotrophic *Methanobrevibacter mcrA* genes were found in hexadecane-amended Mercator MV enrichments (Supplementary Table S3), suggesting the presence of hexadecane-utilizing syntrophic associations. Turnover of hexadecane to methane was also shown to occur in hypersaline sediments of the Napoli MV (Lazar et al., 2011a,b), and terrestrial mud volcanoes from the Carpathian Mountains and Indonesia (Jiménez et al., 2016). It is possible that in high salinity MV sediments with elevated concentrations of alkanes, such as the Mercator MV (Perez-Garcia et al., 2011), methane production from alternative substrates is additionally important. Further experiments with increased incubation times (Katayama et al., 2022) may be necessary to determine the importance of alkanes as a substrate for methanogenesis in the Gulf of Cádiz.

Similarly, methane production from benzoate (Nottingham and Hungate, 1969) was also evident from sediments from several MVs, including Porto, Meknes and Mercator, but detection of the prokaryotes involved remained elusive. Only bacteria and archaea from the Porto MV sediment with benzoate were identified, with sequences mainly belonging to ANME and *Atribacterota* class JS1. Previous studies have shown that methanogenic benzoate degradation to carbon dioxide and methane is mediated by a consortium of bacteria (e.g., *Syntrophu*s species) and hydrogen-utilizing methanogens (Stams and Plugge, 2009). It is therefore fascinating to think that a consortium of *Atribacterota*, thought to be heterotrophic (Nobu et al., 2016) and ANME which can produce methane by CO_2_ reduction under certain conditions (Bertram et al., 2013) could be carrying out complete benzoate degradation through to methane. However, 16S rRNA gene sequences belonging to the genus *Syntrophus* (>0.01% of sequences) and hydrogenotrophic methanogens were found in sediments from all MVs analyzed from the Gulf of Cádiz, and previously 16S rRNA genes of *Syntrophus* species have been retrieved from the Kazan MV (Pachiadaki et al., 2010), suggesting that microbial communities in MVs have the potential to produce methane from aromatic compounds. The lack of a clear identification of methanogens from benzoate amended enrichments may also be due to the need for longer incubations or alternative methods of enrichment (Imachi et al., 2011; Katayama et al., 2022).

## Summary

In summary, mud volcano microbial populations and activities were quite variable in the Gulf of Cádiz, reflecting the heterogeneity within and between individual MVs. There were also marked differences between the microbial biogeochemistry of many MV sediments and their reference sites. Surprisingly, overall direct cell numbers enumerated below the SMTZ (0.2–0.5 mbsf) were much lower than the general global depth distribution for subsurface sediments and were equivalent to cell numbers from depths below 100 mbsf in other subsurface sediments. This may suggest that the environment below the SMTZ is relatively inhospitable for prokaryotes, or that populations may have been transported upwards in fluids or mud breccia from greater depths during episodic eruptions. However, one site at Porto MV (Station 144) was an exception having highly elevated cell numbers at 0.9 mbsf. The general low cell numbers at most MV samples reflected unsuccessful DNA extraction and PCR amplification, and very low detectable rates of methanogenesis from ^14^C-substrates. Interestingly, methanogenic activities from methyl compounds, especially methylamine, were much higher than with H_2_/CO_2_ or acetate which are usually the dominant methanogenic substrates. Consistent with this use of substrates, significant CH_4_ production occurred in 50% of slurry enrichments with added methylated compounds and only methylotrophic CH_4_ production occurred at all seven MV sites. These slurries were dominated by *Methanosarcinales* methanogens related to *Methanococcoides* pure cultures and gene sequences detected in a number of other MVs. AOM also occurred in a number of slurries, particularly, those from Captain Arutyunov, Mercator and Carlos Ribeiro, and was supported by the dominant archaea found. Archaeal diversity at four MV sites showed the presence of both methanogen and ANME (e.g., *Methanosarcinales*, *Methanococcoides*, and ANME-1, respectively) sequences, although their proportions varied within and between MVs. Overall bacterial diversity was higher than archaeal diversity with a greater number of higher taxonomic groups found and was often dominated by *Atribacterota* class JS1. These results demonstrate the biogeochemical complexity of Gulf of Cádiz MV environments and the potential of their prokaryotes to be involved in methane cycling. Further work is essential to determine the full contribution of Gulf of Cádiz MV sediments to CH_4_ emissions and the global methane and carbon cycles.

## Data availability statement

The datasets presented in this study can be found in online repositories. The names of the repository/repositories and accession number(s) can be found in the article/Supplementary material.

## Author contributions

We describe author contributions to the paper using the contributor roles taxonomy (CRediT). GW, AWe, and RP: conceptualization. GW and BC: data curation and formal analysis. AWe and RP: funding acquisition, project administration, resources, and supervision. GW, BC, JR, AWa, and HS: investigation. GW, BC, AWe, and RP: methodology and validation. GW and RP: visualization. GW: writing—original draft. GW, BC, JR, AWa, HS, AWe, and RP: writing—review and editing. All authors contributed to the article and approved the submitted version.

## Funding

This work was supported by the “HERMES” (Hot-Spot Ecosystems along European Margins) project grant funded under FP6 of the European Commission and the Natural Environment Research Council (NERC) grant numbers NE/F018983/1 and NE/J011177/1.

## In Memoriam

This paper is dedicated to the late Dr. Barry A. Cragg, a pioneer in the field of deep biosphere microbiology, his influence on this research was as great as many of his other studies on the deep subseafloor.

## Conflict of interest

The authors declare that the research was conducted in the absence of any commercial or financial relationships that could be construed as a potential conflict of interest.

## Publisher’s note

All claims expressed in this article are solely those of the authors and do not necessarily represent those of their affiliated organizations, or those of the publisher, the editors and the reviewers. Any product that may be evaluated in this article, or claim that may be made by its manufacturer, is not guaranteed or endorsed by the publisher.

## Footnotes

^1^http://www.ncbi.nlm.nih.gov/

^2^http://www.technelysium.com.au/

^3^https://blast.ncbi.nlm.nih.gov/Blast.cgi

## References

[ref1] Adam P. S. Borrel G. Brochier-Armanet C. Gribaldo S. (2017). The growing tree of Archaea: new perspectives on their diversity, evolution and ecology. ISME J. 11, 2407–2425. doi: 10.1038/ismej.2017.122, PMID: 28777382 PMC5649171

[ref2] Baker B. J. De Anda V. Seitz K. W. Dombrowski N. Santoro A. E. Lloyd K. G. (2020). Diversity, ecology and evolution of Archaea. Nat. Microbiol. 5, 887–900. doi: 10.1038/s41564-020-0715-z 32367054

[ref3] Baker B. J. Saw J. H. Lind A. E. Lazar C. S. Hinrichs K. U. Teske A. P.. (2016). Genomic inference of the metabolism of cosmopolitan subsurface Archaea, Hadesarchaea. Nat. Microbiol. 1:16002. doi: 10.1038/nmicrobiol.2016.2 27572167

[ref4] Baloglanov E. E. Abbasov O. R. Akhundov R. V. (2018). Mud volcanoes of the world: classifications, activities and environmental hazard (informational-analytical review). Eur. J. Nat. Hist. 5, 12–26.

[ref5] Beal E. J. House C. H. Orphan V. J. (2009). Manganese- and Iron-dependent marine methane oxidation. Science 325, 184–187. doi: 10.1126/science.1169984 19589998

[ref6] Bertram S. Blumenberg M. Michaelis W. Siegert M. Krüger M. Seifert R. (2013). Methanogenic capabilities of ANME-archaea deduced from ^13^C-labelling approaches. Environ. Microbiol. 15, 2384–2393. doi: 10.1111/1462-2920.12112, PMID: 23530864

[ref7] Beulig F. Røy H. Glombitza C. Jørgensen B. B. (2018). Control on rate and pathway of anaerobic organic carbon degradation in the seabed. Proc. Natl. Acad. Sci. U.S.A. 115, 367–372. doi: 10.1073/pnas.1715789115, PMID: 29279408 PMC5777060

[ref8] Borrel G. Parisot N. Harris H. M. B. Peyretaillade E. Gaci N. Tottey W.. (2014). Comparative genomics highlights the unique biology of Methanomassiliicoccales, a Thermoplasmatales-related seventh order of methanogenic archaea that encodes pyrrolysine. BMC Genomics 15:679. doi: 10.1186/1471-2164-15-679, PMID: 25124552 PMC4153887

[ref9] Brennan R. A. Sanford R. A. (2002). Continuous steady-state method using Tenax for delivering tetrachloroethene to chloro-respiring bacteria. Appl. Environ. Microbiol. 68, 1464–1467. doi: 10.1128/AEM.68.3.1464-1467.2002, PMID: 11872503 PMC123744

[ref10] Bueno de Mesquita C. P. Wu D. Tringe S. G. (2023). Methyl-based Methanogenesis: an ecological and genomic review. Microbiol. Mol. Biol. Rev. 87:e0002422. doi: 10.1128/mmbr.00024-22, PMID: 36692297 PMC10029344

[ref11] Caporaso J. G. Kuczynski J. Stombaugh J. Bittinger K. Bushman F. D. Costello E. K.. (2010). QIIME allows analysis of high-throughput community sequencing data. Nat. Methods 7, 335–336. doi: 10.1038/nmeth.f.303, PMID: 20383131 PMC3156573

[ref12] Caporaso J. G. Lauber C. L. Walters W. A. Berg-Lyons D. Lozupone C. A. Turnbaugh P. J.. (2011). Global patterns of 16S rRNA diversity at a depth of millions of sequences per sample. Proc. Natl. Acad. Sci. U.S.A. 108, 4516–4522. doi: 10.1073/pnas.1000080107, PMID: 20534432 PMC3063599

[ref13] Chang Y. H. Cheng T. W. Lai W. J. Tsai W. Y. Sun C. H. Lin L. H.. (2012). Microbial methane cycling in a terrestrial mud volcano in eastern Taiwan. Environ. Microbiol. 14, 895–908. doi: 10.1111/j.1462-2920.2011.02658.x, PMID: 22141749

[ref14] Chivian D. Brodie E. L. Alm E. J. Culley D. E. Dehal P. S. DeSantis T. Z.. (2008). Environmental genomics reveals a single-species ecosystem deep within earth. Science 322, 275–278. doi: 10.1126/science.1155495, PMID: 18845759

[ref15] Claypool G. E. Kvenvolden K. A. (1983). Methane and other hydrocarbon gases in marine sediment. Annu. Rev. Earth Planet. Sci. 11, 299–327. doi: 10.1146/annurev.ea.11.050183.001503

[ref16] Coelho F. J. R. C. Louvado A. Domingues P. M. Cleary D. F. R. Ferreira M. Almeida A.. (2016). Integrated analysis of bacterial and microeukaryotic communities from differentially active mud volcanoes in the Gulf of Cádiz. Sci. Rep. 6:35272. doi: 10.1038/srep35272, PMID: 27762306 PMC5071872

[ref17] Cragg B. A. Bale S. J. Parkes R. J. (1992). A novel method for the transport and long-term storage of cultures and samples in an anaerobic atmosphere. Lett. Appl. Microbiol. 15, 125–128. doi: 10.1111/j.1472-765X.1992.tb00743.x, PMID: 29389029

[ref18] Crill P. M. Martens C. S. (1986). Methane production from bicarbonate and acetate in an anoxic marine sediment. Geochim. Cosmochim. Acta 50, 2089–2097. doi: 10.1016/0016-7037(86)90262-0

[ref19] Cunha M. R. Rodrigues C. F. Génio L. Hilário A. Ravara A. Pfannkuche O. (2013). Macrofaunal assemblages from mud volcanoes in the Gulf of Cádiz: abundance, biodiversity and diversity partitioning across spatial scales. Biogeosciences 10, 2553–2568. doi: 10.5194/bg-10-2553-2013

[ref20] D’Amore R. Ijaz U. Z. Schirmer M. Kenny J. Gregory R. Darby A. C.. (2016). A comprehensive benchmarking study of protocols and sequencing platforms for 16S rRNA community profiling. BMC Genomics 17:55. doi: 10.1186/s12864-015-2194-9, PMID: 26763898 PMC4712552

[ref21] DeSantis T. Z. Hugenholtz P. Larsen N. Rojas M. Brodie E. L. Keller K.. (2006). Greengenes, a chimera-checked 16S rRNA gene database and workbench compatible with ARB. Appl. Environ. Microbiol. 72, 5069–5072. doi: 10.1128/AEM.03006-05, PMID: 16820507 PMC1489311

[ref22] Dhillon A. Lever M. Lloyd K. G. Albert D. B. Sogin M. L. Teske A. (2005). Methanogen diversity evidenced by molecular characterization of methyl coenzyme M reductase A (*mcrA*) genes in hydrothermal sediments of the Guaymas Basin. Appl. Environ. Microbiol. 71, 4592–4601. doi: 10.1128/AEM.71.8.4592-4601.2005, PMID: 16085853 PMC1183284

[ref23] Díaz-del-Rio V. Somoza L. Martinez-Frias J. Mata M. P. Delgado A. Hernandez-Molina F. J.. (2003). Vast fields of hydrocarbon-derived carbonate chimneys related to the accretionary wedge/olistostrome of the Gulf of Cádiz. Mar. Geol. 195, 177–200. doi: 10.1016/S0025-3227(02)00687-4

[ref24] Dimitrov L. I. (2002). Mud volcanoes - the most important pathway for degassing deeply buried sediments. Earth Sci. Rev. 59, 49–76. doi: 10.1016/S0012-8252(02)00069-7

[ref25] Dimitrov L. I. (2003). Mud volcanoes - a significant source of atmospheric methane. Geo-Mar. Lett. 23, 155–161. doi: 10.1007/s00367-003-0140-3

[ref26] Dolfing J. Larter S. R. Head I. M. (2008). Thermodynamic constraints on methanogenic crude oil biodegradation. ISME J. 2, 442–452. doi: 10.1038/ismej.2007.111 18079730

[ref27] Edgar R. C. (2010). Search and clustering orders of magnitude faster than BLAST. Bioinformatics 26, 2460–2461. doi: 10.1093/bioinformatics/btq461, PMID: 20709691

[ref28] Etiope G. (2009). Natural emissions of methane from geological seepage in Europe. Atmos. Environ. 43, 1430–1443. doi: 10.1016/j.atmosenv.2008.03.014

[ref29] Etiope G. Nakada R. Tanaka K. Yoshida N. (2011). Gas seepage from Tokamachi mud volcanoes, onshore Niigata Basin (Japan): origin, post-genetic alterations and CH_4_-CO_2_ fluxes. Appl. Geochem. 26, 348–359. doi: 10.1016/j.apgeochem.2010.12.008

[ref30] Evans P. N. Parks D. H. Chadwick G. L. Robbins S. J. Orphan V. J. Golding S. D.. (2015). Methane metabolism in the archaeal phylum Bathyarchaeota revealed by genome-centric metagenomics. Science 350, 434–438. doi: 10.1126/science.aac7745, PMID: 26494757

[ref31] Farag I. F. Davis J. P. Youssef N. H. Elshahed M. S. (2014). Global patterns of abundance, diversity and community structure of the Aminicenantes (candidate phylum OP8). PLoS One 9:e92139. doi: 10.1371/journal.pone.0092139, PMID: 24637619 PMC3956909

[ref32] Fowler S. J. Toth C. R. A. Gieg L. M. (2016). Community structure in methanogenic enrichments provides insight into syntrophic interactions in hydrocarbon-impacted environments. Front. Microbiol. 7:562. doi: 10.3389/fmicb.2016.00562 27148240 PMC4840303

[ref33] Fry J. C. (1988). “Determination of biomass” in Methods in aquatic bacteriology. ed. Austin B. (Chichester: John Wiley), 27–77.

[ref34] Fry J. C. Parkes R. J. Cragg B. A. Weightman A. J. Webster G. (2008). Prokaryotic biodiversity and activity in the deep subseafloor biosphere. FEMS Microbiol. Ecol. 66, 181–196. doi: 10.1111/j.1574-6941.2008.00566.x, PMID: 18752622

[ref35] Gardner J. (2001). Mud volcanoes revealed and sampled on the Western Moroccan continental Margin. Geophys. Res. Lett. 28, 339–342. doi: 10.1029/2000GL012141

[ref36] Haffert L. Haeckel M. Liebetrau V. Berndt C. Hensen C. Nuzzo M.. (2013). Fluid evolution and authigenic mineral paragenesis related to salt diapirism - the Mercator mud volcano in the Gulf of Cádiz. Geochim. Cosmochim. Acta 106, 261–286. doi: 10.1016/j.gca.2012.12.016

[ref37] Hallam S. J. Girguis P. R. Preston C. M. Richardson P. M. DeLong E. F. (2003). Identification of methyl coenzyme M reductase A (*mcrA*) genes associated with methane-oxidizing Archaea. Appl. Environ. Microbiol. 69, 5483–5491. doi: 10.1128/AEM.69.9.5483-5491.2003, PMID: 12957937 PMC194966

[ref38] Heijs S. K. Haese R. R. van der Wielen P. W. J. J. Forney L. J. van Elsas J. D. (2007). Use of 16S rRNA gene based clone libraries to assess microbial communities potentially involved in anaerobic methane oxidation in a Mediterranean cold seep. Microb. Ecol. 53, 384–398. doi: 10.1007/s00248-006-9172-3, PMID: 17431711 PMC1915594

[ref39] Heijs S. K. Laverman A. M. Forney L. J. Hardoim P. R. van Elsas J. D. (2008). Comparison of deep-sea sediment microbial communities in the eastern Mediterranean. FEMS Microbiol. Ecol. 64, 362–377. doi: 10.1111/j.1574-6941.2008.00463.x, PMID: 18422633

[ref40] Hoshino T. Toki T. Ijiri A. Morono Y. Machiyama H. Ashi J.. (2017). Atribacteria from the subseafloor sedimentary biosphere disperse to the hydrosphere through submarine mud volcanoes. Front. Microbiol. 8:1135. doi: 10.3389/fmicb.2017.01135, PMID: 28676800 PMC5476839

[ref41] Huse S. M. Welch D. M. Morrison H. G. Sogin M. L. (2010). Ironing out the wrinkles in the rare biosphere through improved OTU clustering. Environ. Microbiol. 12, 1889–1898. doi: 10.1111/j.1462-2920.2010.02193.x, PMID: 20236171 PMC2909393

[ref42] Ijiri A. Inagaki F. Kubo Y. Adhikari R. R. Hattori S. Hoshino T.. (2018). Deep-biosphere methane production stimulated by geofluids in the Nankai accretionary complex. Sci. Adv. 4:eaao4631. doi: 10.1126/sciadv.aao4631 29928689 PMC6007163

[ref43] Imachi H. Aoi K. Tasumi E. Saito Y. Yamanaka Y. Saito Y.. (2011). Cultivation of methanogenic community from subseafloor sediments using a continuous-flow bioreactor. ISME J. 5, 1913–1925. doi: 10.1038/ismej.2011.64, PMID: 21654849 PMC3223304

[ref44] Inagaki F. Nunoura T. Nakagawa S. Teske A. Lever M. Lauer A.. (2006). Biogeographical distribution and diversity of microbes in methane hydrate-bearing deep marine sediments on the Pacific Ocean Margin. Proc. Natl. Acad. Sci. U.S.A. 103, 2815–2820. doi: 10.1073/pnas.0511033103, PMID: 16477011 PMC1413818

[ref45] Jameson E. Stephenson J. Jones H. Millard A. Kaster A. K. Purdy K. J.. (2019). *Deltaproteobacteria* (*Pelobacter*) and *Methanococcoides* are responsible for choline-dependent methanogenesis in a coastal saltmarsh sediment. ISME J. 13, 277–289. doi: 10.1038/s41396-018-0269-8, PMID: 30206424 PMC6331629

[ref46] Jiménez N. Richnow H. H. Vogt C. Treude T. Krüger M. (2016). Methanogenic hydrocarbon degradation: evidence from field and laboratory studies. J. Mol. Microbiol. Biotechnol. 26, 227–242. doi: 10.1159/000441679, PMID: 26959375

[ref47] Jones H. J. Kröber E. Stephenson J. Mausz M. A. Jameson E. Millard A.. (2019). A new family of uncultivated bacteria involved in methanogenesis from the ubiquitous osmolyte glycine betaine in coastal saltmarsh sediments. Microbiome 7:120. doi: 10.1186/s40168-019-0732-4, PMID: 31464644 PMC6716910

[ref48] Joshi NA Fass JN (2011). Sickle: A sliding-window, adaptive, quality-based trimming tool for FastQ files [software].

[ref49] Joye S. B. (2020). “The geology and biogeochemistry of hydrocarbon seeps” in Annual review of earth and planetary sciences, vol. 48 (Annual Reviews Inc), 205–231. doi: 10.1146/annurev-arth-063016-020052

[ref50] Joye S. B. Samarkin V. A. Orcutt B. N. MacDonald I. R. Hinrichs K. U. Elvert M.. (2009). Metabolic variability in seafloor brines revealed by carbon and Sulphur dynamics. Nat. Geosci. 2, 349–354. c

[ref51] Judd A. (2005). “Gas emissions from mud volcanoes” in Mud volcanoes, geodynamics and seismicity. eds. Martinelli G. Panahi B., NATO Science Series (Series IV: Earth and Environmental Series), vol. 51 (Dordrecht: Springer), 147–157.

[ref52] Karnachuk O. V. Frank Y. A. Lukina A. P. Kadnikov V. V. Beletsky A. V. Mardanov A. V.. (2019). Domestication of previously uncultivated *Candidatus* Desulforudis audaxviator from a deep aquifer in Siberia sheds light on its physiology and evolution. ISME J. 13, 1947–1959. doi: 10.1038/s41396-019-0402-3, PMID: 30899075 PMC6776058

[ref53] Katayama T. Yoshioka H. Kaneko M. Amo M. Fujii T. Takahashi H. A.. (2022). Cultivation and biogeochemical analyses reveal insights into methanogenesis in deep subseafloor sediment at a biogenic gas hydrate site. ISME J. 16, 1464–1472. doi: 10.1038/s41396-021-01175-7, PMID: 35105960 PMC9038717

[ref54] Katayama T. Yoshioka H. Takahashi H. A. Amo M. Fujii T. Sakata S. (2016). Changes in microbial communities associated with gas hydrates in subseafloor sediments from the Nankai Trough. FEMS Microbiol. Ecol. 92:fiw093. doi: 10.1093/femsec/fiw093 27170363

[ref55] Kenyon NH Ivanov MK Akhmetzhanov AM Akhmanov GG (2000) Multidisciplinary study of geological processes on the North East Atlantic and Western Mediterranean Margins. IOC Technical Series No. 56.

[ref56] King G. M. Klug M. J. Lovely D. R. (1983). Metabolism of acetate, methanol, and methylated amines in intertidal sediments of Lowes Cove, Maine. Appl. Environ. Microbiol. 45, 1848–1853. doi: 10.1128/aem.45.6.1848-1853.1983, PMID: 16346317 PMC242548

[ref001] Knittel K. Boetius A. (2009). Anaerobic oxidation of methane: progress with an unknown process. Annu Rev Microbiol. 63, 311–34. doi: 10.1146/annurev.micro.61.080706.093130 19575572

[ref57] Kopf A. J. (2002). Significance of mud volcanism. Rev. Geophys. 40, 2-1–2-52. doi: 10.1029/2000RG000093

[ref58] Kopf A. J. (2003). Global methane emission through mud volcanoes and its past and present impact on the Earth's climate. Int. J. Earth Sci. 92, 806–816. doi: 10.1007/s00531-003-0341-z

[ref59] Köpke B. Wilms R. Engelen B. Cypionka H. Sass H. (2005). Microbial diversity in coastal subsurface sediments – a cultivation approach using various electron acceptors and substrate gradients. Appl. Environ. Microbiol. 71, 7819–7830. doi: 10.1128/AEM.71.12.7819-7830.2005, PMID: 16332756 PMC1317335

[ref60] Krüger M. Treude T. Wolters H. Nauhaus K. Boetius A. (2005). Microbial methane turnover in different marine habitats. Palaeogeogr. Palaeoclimatol. Palaeoecol. 227, 6–17. doi: 10.1016/j.palaeo.2005.04.031

[ref61] L’Haridon S. Chalopin M. Colombo D. Toffin L. (2014). *Methanococcoides vulcani* sp. nov., a marine methylotrophic methanogen that uses betaine, choline and *N,N*-dimethylethanolamine for methanogenesis, isolated from a mud volcano, and emended description of the genus *Methanococcoides*. Int. J. Syst. Evol. Microbiol. 64, 1978–1983. doi: 10.1099/ijs.0.058289-0, PMID: 24614846

[ref62] Lazar C. S. L’Haridon S. Pignet P. Toffin L. (2011a). Archaeal populations in hypersaline sediments underlying orange microbial mats in the Napoli mud volcano. Appl. Environ. Microbiol. 77, 3120–3131. doi: 10.1128/AEM.01296-10, PMID: 21335391 PMC3126394

[ref63] Lazar C. S. Parkes R. J. Cragg B. A. L’Haridon S. Toffin L. (2011b). Methanogenic diversity and activity in hypersaline sediments of the Centre of the Napoli mud volcano, eastern Mediterranean Sea. Environ. Microbiol. 13, 2078–2091. doi: 10.1111/j.1462-2920.2011.02425.x, PMID: 21382146

[ref64] Lazar C. S. Parkes R. J. Cragg B. A. L’Haridon S. Toffin L. (2012). Methanogenic activity and diversity in the Centre of the Amsterdam mud volcano, Eastern Mediterranean Sea. FEMS Microbiol. Ecol. 81, 243–254. doi: 10.1111/j.1574-6941.2012.01375.x, PMID: 22458514

[ref65] Lee D. H. Lee Y. M. Kim J. H. Jin Y. K. Paull C. Niemann H.. (2019). Discriminative biogeochemical signatures of methanotrophs in different chemosynthetic habitats at an active mud volcano in the Canadian Beaufort Sea. Sci. Rep. 9:17592. doi: 10.1038/s41598-019-53950-4 31772218 PMC6879587

[ref66] León R. Somoza L. Medialdea T. Vázquez J. T. González F. J. López-González N.. (2012). New discoveries of mud volcanoes on the Moroccan Atlantic continental margin (Gulf of Cádiz): morpho-structural characterization. Geo-Mar. Lett. 32, 473–488. doi: 10.1007/s00367-012-0275-1

[ref67] Liu Y. Whitman W. B. (2008). Metabolic, phylogenetic, and ecological diversity of the methanogenic *Archaea*. Ann. N. Y. Acad. Sci. 1125, 171–189. doi: 10.1196/annals.1419.019 18378594

[ref68] Lösekann T. Robador A. Niemann H. Knittel K. Boetius A. Dubilier N. (2008). Endosymbioses between bacteria and deep-sea siboglinid tubeworms from an Arctic cold seep (Haakon Mosby mud volcano, Barents Sea). Environ. Microbiol. 10, 3237–3254. doi: 10.1111/j.1462-2920.2008.01712.x, PMID: 18707616

[ref69] Lyimo T. J. Pol A. Jetten M. S. M. Op den Camp H. J. M. (2009). Diversity of methanogenic archaea in a mangrove sediment and isolation of a new *Methanococcoides* strain. FEMS Microbiol. Lett. 291, 247–253. doi: 10.1111/j.1574-6968.2008.01464.x, PMID: 19146579

[ref70] Magalhães V. H. Pinheiroa L. M. Ivanov M. K. Kozlova E. Blinova V. Kolganova J.. (2012). Formation processes of methane-derived authigenic carbonates from the Gulf of Cádiz. Sediment. Geol. 243-244, 155–168. doi: 10.1016/j.sedgeo.2011.10.013

[ref71] Mah R. A. Ward D. M. Baresi L. Glass T. L. (1977). Biogenesis of methane. Annu. Rev. Microbiol. 31, 309–341. doi: 10.1146/annurev.mi.31.100177.001521 20832

[ref72] Maignien L. Parkes R. J. Cragg B. Niemann H. Knittel K. Coulon S.. (2013). Anaerobic oxidation of methane in hypersaline cold seep sediments. FEMS Microbiol. Ecol. 83, 214–231. doi: 10.1111/j.1574-6941.2012.01466.x, PMID: 22882187

[ref73] Maldonado A. Somoza L. Pallares L. (1999). The Betic orogen and the Iberian–African boundary in the Gulf of Cadiz: geological evolution (Central North Atlantic). Mar. Geol. 155, 9–43. doi: 10.1016/S0025-3227(98)00139-X

[ref74] Martin M. (2011). Cutadapt removes adapter sequences from high-throughput sequencing reads. EMBnet j. 17, 10–12. doi: 10.14806/ej.17.1.200

[ref75] Martinez R. J. Mills H. J. Story S. Sobecky P. A. (2006). Prokaryotic diversity and metabolically active microbial populations in sediments from an active mud volcano in the Gulf of Mexico. Environ. Microbiol. 8, 1783–1796. doi: 10.1111/j.1462-2920.2006.01063.x, PMID: 16958759

[ref76] Mazzini A. Etiope G. (2017). Mud volcanism: an updated review. Earth Sci. Rev. 168, 81–112. doi: 10.1016/j.earscirev.2017.03.001

[ref77] Medialdea T. Somoza L. Pinheiro L. M. Fernández-Puga M. C. Vázquez J. T. León R.. (2009). Tectonics and mud volcano development in the Gulf of Cádiz. Mar. Geol. 261, 48–63. doi: 10.1016/j.margeo.2008.10.007

[ref78] Meng J. Xu J. Qin D. He Y. Xiao X. Wang F. (2014). Genetic and functional properties of uncultivated MCG archaea assessed by metagenome and gene expression analyses. ISME J. 8, 650–659. doi: 10.1038/ismej.2013.174, PMID: 24108328 PMC3930316

[ref79] Milkov A. V. (2000). Worldwide distribution of submarine mud volcanoes and associated gas hydrates. Mar. Geol. 167, 29–42. doi: 10.1016/S0025-3227(00)00022-0

[ref80] Niemann H. Duarte J. Hensen C. Omoregie E. Magalhães V. H. Elvert M.. (2006a). Microbial methane turnover at mud volcanoes of the Gulf of Cádiz. Geochim. Cosmochim. Acta 70, 5336–5355. doi: 10.1016/j.gca.2006.08.010

[ref81] Niemann H. Lösekann T. de Beer D. Elvert M. Nadalig T. Knittel K.. (2006b). Novel microbial communities of the Haakon Mosby mud volcano and their role as a methane sink. Nature 443, 854–858. doi: 10.1038/nature05227, PMID: 17051217

[ref82] Nobu M. K. Dodsworth J. A. Murugapiran S. K. Rinke C. Gies E. A. Webster G.. (2016). Phylogeny and physiology of candidate phylum ‘Atribacteria’ (OP9/JS1) inferred from cultivation-independent genomics. ISME J. 10, 273–286. doi: 10.1038/ismej.2015.97, PMID: 26090992 PMC4737943

[ref83] Nottingham P. M. Hungate R. E. (1969). Methanogenic fermentation of benzoate. J. Bacteriol. 98, 1170–1172. doi: 10.1128/jb.98.3.1170-1172.1969, PMID: 5788702 PMC315310

[ref84] Nuzzo M. Hornibrook E. R. C. Gill F. Hensen C. Pancost R. Haeckel M.. (2009). Origin of light volatile hydrocarbon gases in mud volcano fluids, Gulf of Cádiz - evidence for multiple sources and transport mechanisms in active sedimentary wedges. Chem. Geol. 266, 350–363. doi: 10.1016/j.chemgeo.2009.06.023

[ref85] Nuzzo M. Tomonaga Y. Schmidt M. Valadares V. Faber E. Piñero E.. (2019). Formation and migration of hydrocarbons in deeply buried sediments of the Gulf of Cádiz convergent plate boundary - insights from the hydrocarbon and helium isotope geochemistry of mud volcano fluids. Mar. Geol. 410, 56–69. doi: 10.1016/j.margeo.2019.01.005

[ref86] Omoregie E. O. Mastalerz V. de Lange G. Straub K. L. Kappler A. Røy H.. (2008). Biogeochemistry and community composition of iron- and sulfur-precipitating microbial mats at the Chefren mud volcano (Nile Deep Sea Fan, Eastern Mediterranean). Appl. Environ. Microbiol. 74, 3198–3215. doi: 10.1128/AEM.01751-07, PMID: 18378658 PMC2394935

[ref87] Orcutt B. N. Joye S. B. Kleindienst S. Knittel K. Ramette A. Rietz A.. (2010). Impact of natural oil and higher hydrocarbons on microbial diversity, distribution, and activity in Gulf of Mexico cold-seep sediments. Deep-Sea Res. II Top. Stud. Oceanogr. 57, 2008–2021. doi: 10.1016/j.dsr2.2010.05.014

[ref88] Oremland R. S. Marsh L. M. Polcin S. (1982). Methane production and simultaneous sulphate reduction in anoxic, salt marsh sediments. Nature 296, 143–145. doi: 10.1038/296143a0

[ref89] O’Sullivan L. A. Webster G. Fry J. C. Parkes J. C. Weightman A. J. (2008). Modified linker-PCR primers facilitate complete sequencing of DGGE DNA fragments. J. Microbiol. Methods 75, 579–581. doi: 10.1016/j.mimet.2008.08.006, PMID: 18789360

[ref90] Pachiadaki M. G. Kallionaki A. Dählmann A. de Lange G. J. Kormas K. A. (2011). Diversity and spatial distribution of prokaryotic communities along a sediment vertical profile of a deep-sea mud volcano. Microb. Ecol. 62, 655–668. doi: 10.1007/s00248-011-9855-2, PMID: 21538105

[ref91] Pachiadaki M. G. Kormas K. A. (2013). Interconnectivity vs. isolation of prokaryotic communities in European deep-sea mud volcanoes. Biogeosciences 10, 2821–2831. doi: 10.5194/bg-10-2821-2013

[ref92] Pachiadaki M. G. Lykousis V. Stefanou E. G. Kormas K. A. (2010). Prokaryotic community structure and diversity in the sediments of an active submarine mudvolcano (Kazan mud volcano, East Mediterranean Sea). FEMS Microbiol. Ecol. 72, 429–444. doi: 10.1111/j.1574-6941.2010.00857.x, PMID: 20370830

[ref93] Palomino D. López-González N. Vázquez J. T. Fernández-Salas L. M. Rueda J. L. Sánchez-Leal R.. (2016). Multidisciplinary study of mud volcanoes and diapirs and their relationship to seepages and bottom currents in the Gulf of Cádiz continental slope (northeastern sector). Mar. Geol. 378, 196–212. doi: 10.1016/j.margeo.2015.10.001

[ref94] Pancost R. D. Sinninghe Damste J. S. de Lint S. van der Maarel M. J. E. C. Gottschal J. C. the Medinaut Shipboard Scientific Party (2000). Biomarker evidence for widespread anaerobic methane oxidation in Mediterranean sediments by a consortium of methanogenic Archaea and Bacteria. Appl. Environ. Microbiol. 66, 1126–1132. doi: 10.1128/AEM.66.3.1126-1132.2000, PMID: 10698781 PMC91952

[ref95] Parkes R. J. Cragg B. A. Banning N. Brock F. Webster G. Fry J. C.. (2007). Biogeochemistry and biodiversity of methane cyclingin subsurface marine sediments (Skagerrak, Denmark). Environ. Microbiol. 9, 1146–1161. doi: 10.1111/j.1462-2920.2006.01237.x, PMID: 17472631

[ref96] Parkes R. J. Cragg B. Roussel E. Webster G. Weightman A. Sass H. (2014). A review of prokaryotic populations and processes in sub-seafloor sediments, including biosphere:geosphere interactions. Mar. Geol. 352, 409–425. doi: 10.1016/j.margeo.2014.02.009

[ref97] Parkes R. J. Cragg B. A. Wellsbury P. (2000). Recent studies on bacterial populations and processes in subseafloor sediments: a review. Hydrogeol. J. 8, 11–28. doi: 10.1007/PL00010971

[ref98] Parkes R. J. Sass H. Webster G. Watkins A. J. Weightman A. J. O’Sullivan L. A.. (2010). “Methods for studying methanogens and methanogenesis in marine sediments” in Handbook of hydrocarbon and lipid microbiology. ed. Timmis K. N. (Berlin: Springer-Verlag), 3799–3826.

[ref99] Perez-Garcia C. Berndt C. Klaeschen D. Mienert J. Haffert L. Depreiter D.. (2011). Linked halokinesis and mud volcanism at the Mercator mud volcano, gulf of Cádiz. J. Geophys. Res. 116:B05101. doi: 10.1029/2010JB008061

[ref100] Pimenov N. Savvichev A. Rusanov I. Lein A. Egorov A. Gebruk A.. (1999). Microbial processes of carbon cycle as the base of food chain of Håkon Mosby mud volcano benthic community. Geo-Mar. Lett. 19, 89–96. doi: 10.1007/s003670050097

[ref101] Pinheiro L. M. Ivanov M. K. Sautkin A. Akhmanov G. Magalhães V. H. Volkonskaya A.. (2003). Mud volcanism in the Gulf of Cádiz: results from the TTR-10 cruise. Mar. Geol. 195, 131–151. doi: 10.1016/S0025-3227(02)00685-0

[ref102] Purdy K. J. Munson M. A. Cresswell-Maynard T. Nedwell D. B. Embley T. M. (2003). Use of 16S rRNA-targeted oligonucleotide probes to investigate function and phylogeny of sulphate-reducing bacteria and methanogenic archaea in a UK estuary. FEMS Microbiol. Ecol. 44, 361–371. doi: 10.1016/S0168-6496(03)00078-3 19719617

[ref103] Rinke C. Rubino F. Messer L. F. Youssef N. Parks D. H. Chuvochina M.. (2019). A phylogenomic and ecological analysis of the globally abundant marine group II archaea (Ca. Poseidoniales ord. nov.). ISME J. 13, 663–675. doi: 10.1038/s41396-018-0282-y, PMID: 30323263 PMC6461757

[ref104] Rodrigues C. F. Hilário A. Cunha M. R. Weightman A. J. Webster G. (2011). Microbial diversity in *Frenulata* (Siboglinidae, Polychaeta) species from mud volcanoes in the Gulf of Cádiz (NE Atlantic). Antonie Van Leeuwenhoek 100, 83–98. doi: 10.1007/s10482-011-9567-0, PMID: 21359663

[ref105] Rodrigues C. F. Webster G. Cunha M. R. Duperron S. Weightman A. J. (2010). Chemosynthetic bacteria found in bivalve species from mud volcanoes of the Gulf of Cádiz. FEMS Microbiol. Ecol. 73, 486–499. doi: 10.1111/j.1574-6941.2010.00913.x, PMID: 20550577

[ref106] Roussel E. G. Sauvadet A. L. Allard J. Chaduteau C. Richard P. Bonavita M. A. C.. (2009). Archaeal methane cycling communities associated with gassy subsurface sediments of Marennes-Oléron Bay (France). Geomicrobiol J. 26, 31–43. doi: 10.1080/01490450802599284

[ref107] Ruff S. E. Felden J. Gruber-Vodicka H. R. Marcon Y. Knittel K. Ramette A.. (2019). In situ development of a methanotrophic microbiome in deep-sea sediments. ISME J. 13, 197–213. doi: 10.1038/s41396-018-0263-1, PMID: 30154496 PMC6298960

[ref108] Sauter E. J. Muyakshin S. I. Charlou J. L. Schlüter M. Boetius A. Jerosch K.. (2006). Methane discharge from a deep-sea submarine mud volcano into the upper water column by gas hydrate-coated methane bubbles. Earth Planet. Sci. Lett. 243, 354–365. doi: 10.1016/j.epsl.2006.01.041

[ref109] Sogin M. L. Morrison H. G. Huber J. A. Welch D. M. Huse S. M. Neal P. R.. (2006). Microbial diversity in the deep sea and the underexplored “rare biosphere”. Proc. Natl. Acad. Sci. U.S.A. 103, 12115–12120. doi: 10.1073/pnas.0605127103, PMID: 16880384 PMC1524930

[ref110] Somoza L. Díaz-del-Rio V. León R. Ivanov M. Fernández-Puga M. C. Gardner J. M.. (2003). Seabed morphology and hydrocarbon seepage in the Gulf of Cádiz mud volcano area: acoustic imagery, multibeam and ultra-high resolution seismic data. Mar. Geol. 195, 153–176. doi: 10.1016/S0025-3227(02)00686-2

[ref111] Stams A. J. M. Hansen T. A. (1984). Fermentation of glutamate and other compounds by *Acidaminobacter hydrogenoformans* gen.Nov., sp.nov., an obligate anaerobe isolated from black mud. Studies with pure cultures and mixed cultures with sulfate-reducing and methanogenic bacteria. Arch. Microbiol. 137, 329–337. doi: 10.1007/BF00410730

[ref112] Stams A. J. M. Plugge C. M. (2009). Electron transfer in syntrophic communities of anaerobic bacteria and archaea. Nat. Rev. Microbiol. 7, 568–577. doi: 10.1038/nrmicro2166 19609258

[ref113] Stewart F. J. Newton I. L. G. Cavanaugh C. M. (2005). Chemosynthetic endosymbioses adaptations to oxic-anoxic interfaces. Trends Microbiol. 13, 439–448. doi: 10.1016/j.tim.2005.07.007, PMID: 16054816

[ref114] Takai K. Moser D. P. DeFlaun M. Onstott T. C. Fredrickson J. K. (2001). Archaeal diversity in waters from deep South African gold mines. Appl. Environ. Microbiol. 67, 5750–5760. doi: 10.1128/AEM.67.21.5750-5760.2001, PMID: 11722932 PMC93369

[ref115] Teske A. Sørensen K. B. (2008). Uncultured archaea in deep marine subsurface sediments: have we caught them all? ISME J. 2, 3–18. doi: 10.1038/ismej.2007.90, PMID: 18180743

[ref116] Teske A. Wegener G. Chanton J. P. White D. Macgregor B. Hoer D.. (2021). Microbial communities under distinct thermal and geochemical regimes in axial and off-axis sediments of Guaymas Basin. Front. Microbiol. 12:633649. doi: 10.3389/fmicb.2021.633649, PMID: 33643265 PMC7906980

[ref117] Valentine D. L. (2011). Emerging topics in marine methane biogeochemistry. Annu. Rev. Mar. Sci. 3, 147–171. doi: 10.1146/annurev-marine-120709-142734 21329202

[ref118] Vanwonterghem I. Evans P. N. Parks D. H. Jensen P. D. Woodcroft B. J. Hugenholtz P.. (2016). Methylotrophic methanogenesis discovered in the archaeal phylum Verstraetearchaeota. Nat. Microbiol. 1:16170. doi: 10.1038/nmicrobiol.2016.170, PMID: 27694807

[ref119] Vigneron A. L'Haridon S. Godfroy A. Roussel E. G. Cragg B. A. Parkes R. J.. (2015). Evidence of active methanogen communities in shallow sediments of the Sonora Margin cold seeps. Appl. Environ. Microbiol. 81, 3451–3459. doi: 10.1128/AEM.00147-15, PMID: 25769831 PMC4407212

[ref120] Wallmann K. Drews M. Aloisi G. Bohrmann G. (2006). Methane discharge into the Black Sea and the global ocean via fluid flow through submarine mud volcanoes. Earth Planet. Sci. Lett. 248, 545–560. doi: 10.1016/j.epsl.2006.06.026

[ref121] Watkins A. J. Roussel E. G. Parkes R. J. Sass H. (2014). Glycine betaine as a direct substrate for methanogens (*Methanococcoides* spp.). Appl. Environ. Microbiol. 80, 289–293. doi: 10.1128/AEM.03076-13, PMID: 24162571 PMC3911008

[ref122] Watkins A. J. Roussel E. G. Webster G. Parkes R. J. Sass H. (2012). Choline and *NN*-dimethylethanolamine as direct substrates for methanogens. Appl. Environ. Microbiol. 78, 8298–8303. doi: 10.1128/AEM.01941-12, PMID: 23001649 PMC3497383

[ref123] Webster G. Blazejak A. Cragg B. A. Schippers A. Sass H. Rinna J.. (2009). Subsurface microbiology and biogeochemistry of a deep, cold-water carbonate mound from the porcupine Seabight (IODP expedition 307). Environ. Microbiol. 11, 239–257. doi: 10.1111/j.1462-2920.2008.01759.x, PMID: 18826439 PMC3638347

[ref124] Webster G. Mullins A. J. Watkins A. J. Cunningham-Oakes E. Weightman A. J. Mahenthiralingam E.. (2019). Genome sequences of two choline-utilizing methanogenic archaea, *Methanococcoides* spp., isolated from marine sediments. Microbiol. Resour. Announc. 8, e00342–e00319. doi: 10.1128/MRA.00342-19 31048384 PMC6498239

[ref125] Webster G. Newberry C. J. Fry J. C. Weightman A. J. (2003). Assessment of bacterial community structure in the deep sub-seafloor biosphere by 16S rDNA-based techniques: a cautionary tale. J. Microbiol. Methods 55, 155–164. doi: 10.1016/S0167-7012(03)00140-4, PMID: 14500007

[ref126] Webster G. O'Sullivan L. A. Meng Y. Williams A. S. Sass A. M. Watkins A. J.. (2015). Archaeal community diversity and abundance changes along a natural salinity gradient in estuarine sediments. FEMS Microbiol. Ecol. 91, 1–18. doi: 10.1093/femsec/fiu025 PMC4399439 25764553

[ref127] Webster G. Parkes R. J. Cragg B. A. Newberry C. J. Weightman A. J. Fry J. C. (2006). Prokaryotic community composition and biogeochemical processes in deep subseafloor sediments from the Peru Margin. FEMS Microbiol. Ecol. 58, 65–85. doi: 10.1111/j.1574-6941.2006.00147.x, PMID: 16958909

[ref128] Webster G. Parkes R. J. Fry J. C. Weightman A. J. (2004). Widespread occurrence of a novel division of bacteria identified by 16 S rRNA gene sequences originally found in deep marine sediments. Appl. Environ. Microbiol. 70, 5708–5713. doi: 10.1128/AEM.70.9.5708-5713.2004, PMID: 15345467 PMC520855

[ref129] Webster G. Sass H. Cragg B. A. Gorra R. Knab N. J. Green C. J.. (2011). Enrichment and cultivation of prokaryotes associated with the sulphate-methane transition zone of diffusion-controlled sediments of Aarhus Bay, Denmark, under heterotrophic conditions. FEMS Microbiol. Ecol. 77, 248–263. doi: 10.1111/j.1574-6941.2011.01109.x 21477007

[ref130] Wellsbury P. Mather I. Parkes R. J. (2002). Geomicrobiology of deep, low organic carbon sediments in the Woodlark Basin, Pacific Ocean. FEMS Microbiol. Ecol. 42, 59–70. doi: 10.1111/j.1574-6941.2002.tb00995.x 19709266

[ref131] Whiticar M. J. Faber E. Schoell M. (1986). Biogenic methane formation in marine and freshwater environments: CO_2_ reduction vs. acetate fermentation-isotope evidence. Geochim. Cosmochim. Acta 50, 693–709. doi: 10.1016/0016-7037(86)90346-7

[ref132] Xiao K. Q. Moore O. W. Babakhani P. Curti L. Peacock C. L. (2022). Mineralogical control on methylotrophic methanogenesis and implications for cryptic methane cycling in marine surface sediment. Nat. Commun. 13:272. doi: 10.1038/s41467-022-30422-4 35581283 PMC9114137

[ref133] Xu L. Zhuang G. C. Montgomery A. Liang Q. Joye S. B. Wang F. (2021). Methyl-compounds driven benthic carbon cycling in the sulfate-reducing sediments of South China Sea. Environ. Microbiol. 23, 641–651. doi: 10.1111/1462-2920.15110, PMID: 32506654

[ref134] Yanagawa K. Sunamura M. Lever M. A. Morono Y. Hiruta A. Ishizaki O.. (2011). Niche separation of methanotrophic Archaea (ANME-1 and -2) in methane-seep sediments of the eastern Japan Sea offshore Joetsu. Geomicrobiol J. 28, 118–129. doi: 10.1080/01490451003709334

[ref135] Yanagawa K. Tani A. Yamamoto N. Hachikubo A. Kano A. Matsumoto R.. (2016). Biogeochemical cycle of methanol in anoxic deep-sea sediments. Microbes Environ. 31, 190–193. doi: 10.1264/jsme2.ME15204, PMID: 27301420 PMC4912158

[ref136] Yoshioka H. Maruyama A. Nakamura T. Higashi Y. Fuse H. Sakata S.. (2010). Activities and distribution of methanogenic and methane-oxidizing microbes in marine sediments from the Cascadia margin. Geobiology 8, 223–233. doi: 10.1111/j.1472-4669.2009.00231.x, PMID: 20059557

[ref137] Zengler K. Richnow H. H. Rosselló-Mora R. Michaelis W. Widdel F. (1999). Methane formation from long-chain alkanes by anaerobic microorganisms. Nature 401, 266–269. doi: 10.1038/45777, PMID: 10499582

[ref138] Zhuang G. C. Heuer V. B. Lazar C. S. Goldhammer T. Wendt J. Samarkin V. A.. (2018). Relative importance of methylotrophic methanogenesis in sediments of the Western Mediterranean Sea. Geochim. Cosmochim. Acta 224, 171–186. doi: 10.1016/j.gca.2017.12.024

