# Supplementary material for: Methanogen activity and microbial diversity in Gulf of Cádiz mud volcano sediments
Source: Front Microbiol. 2023 May 24;14:1157337. doi: 10.3389/fmicb.2023.1157337 (PMC10244519; doi:10.3389/fmicb.2023.1157337)
Supplement: Supplementary file 1 [file Presentation_1.PPTX]

## Slide 1
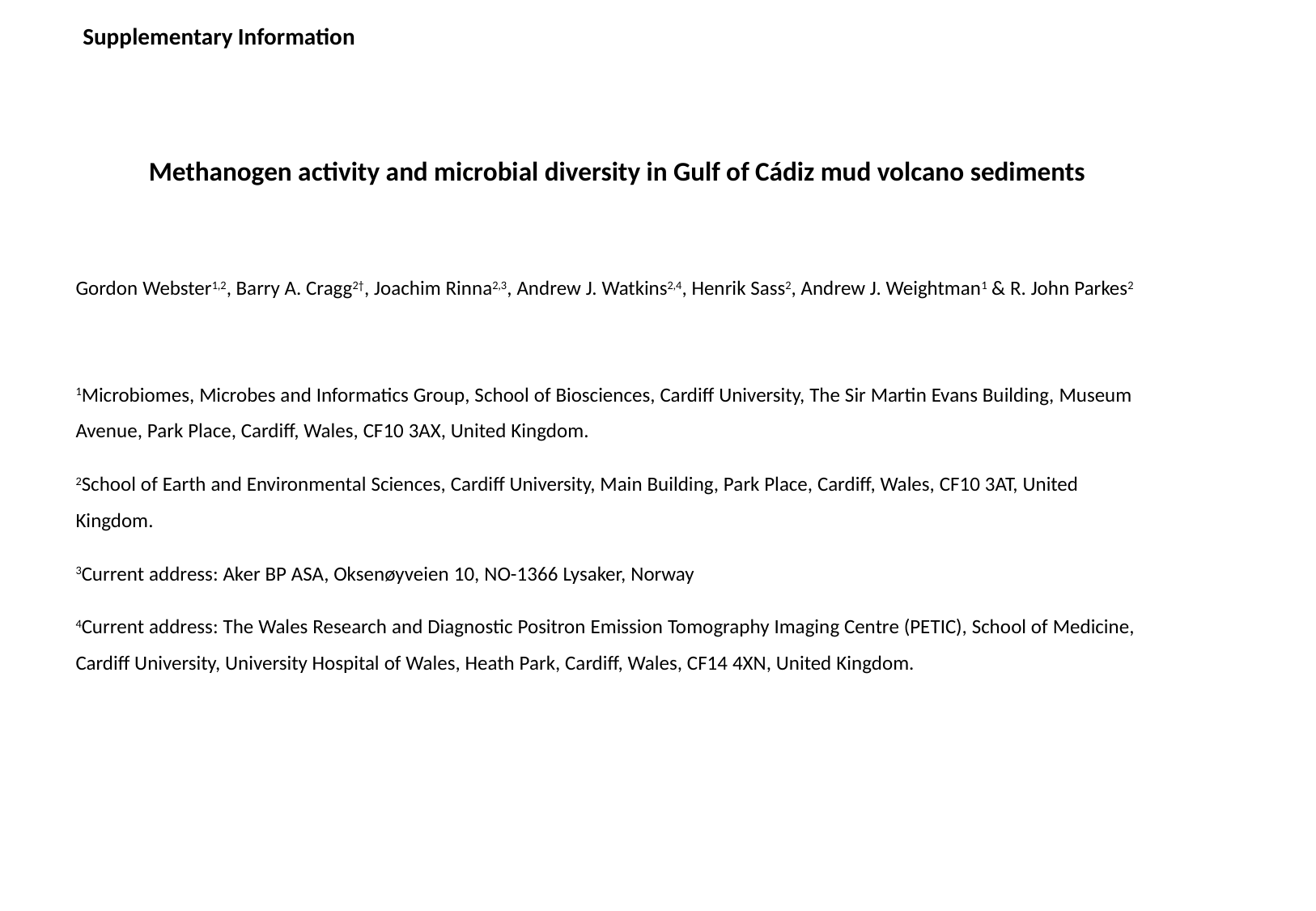

Supplementary Information
Methanogen activity and microbial diversity in Gulf of Cádiz mud volcano sediments
Gordon Webster1,2, Barry A. Cragg2†, Joachim Rinna2,3, Andrew J. Watkins2,4, Henrik Sass2, Andrew J. Weightman1 & R. John Parkes2
1Microbiomes, Microbes and Informatics Group, School of Biosciences, Cardiff University, The Sir Martin Evans Building, Museum Avenue, Park Place, Cardiff, Wales, CF10 3AX, United Kingdom.
2School of Earth and Environmental Sciences, Cardiff University, Main Building, Park Place, Cardiff, Wales, CF10 3AT, United Kingdom.
3Current address: Aker BP ASA, Oksenøyveien 10, NO-1366 Lysaker, Norway
4Current address: The Wales Research and Diagnostic Positron Emission Tomography Imaging Centre (PETIC), School of Medicine, Cardiff University, University Hospital of Wales, Heath Park, Cardiff, Wales, CF14 4XN, United Kingdom.

## Slide 2
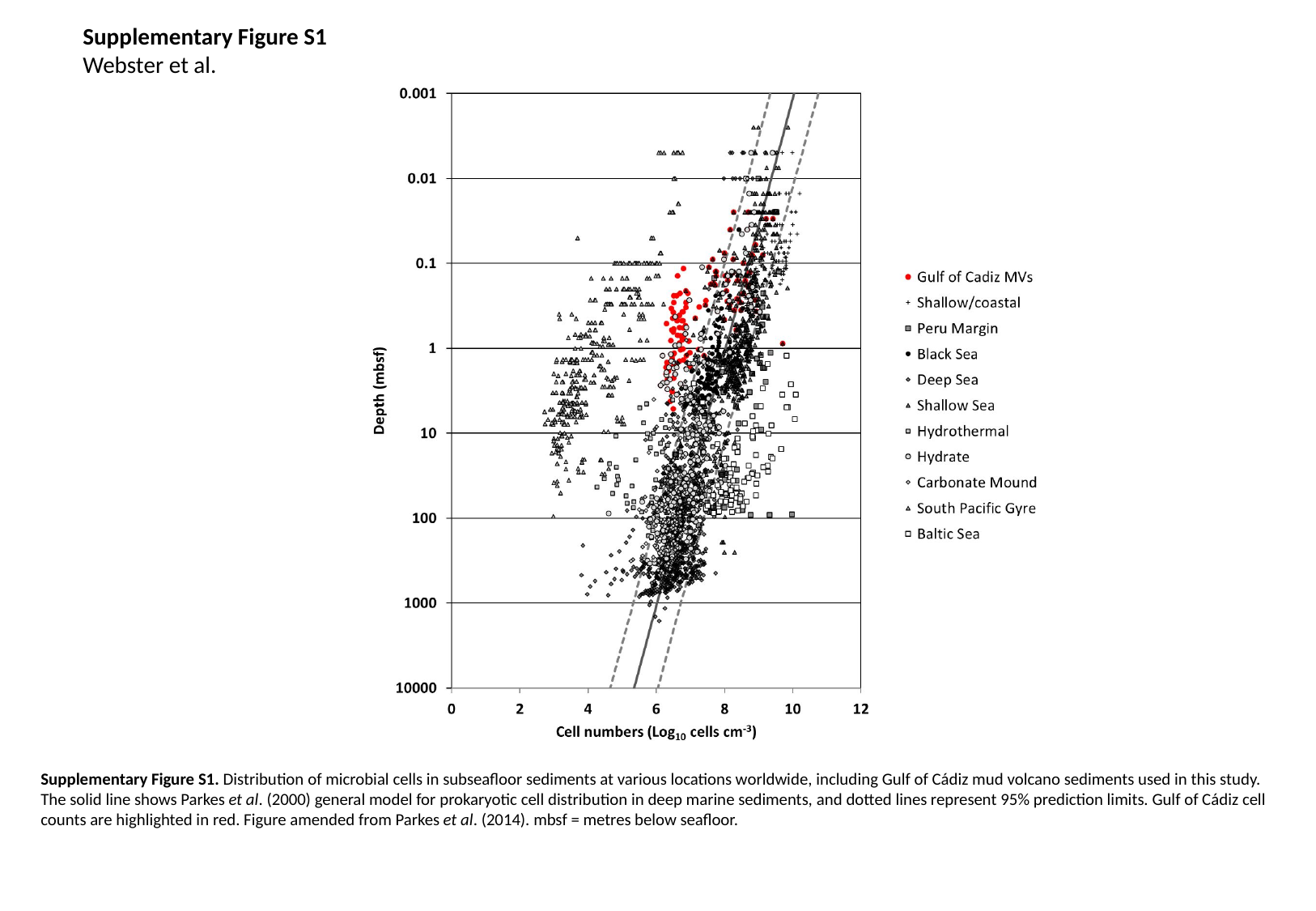

Supplementary Figure S1		Webster et al.
Supplementary Figure S1. Distribution of microbial cells in subseafloor sediments at various locations worldwide, including Gulf of Cádiz mud volcano sediments used in this study. The solid line shows Parkes et al. (2000) general model for prokaryotic cell distribution in deep marine sediments, and dotted lines represent 95% prediction limits. Gulf of Cádiz cell counts are highlighted in red. Figure amended from Parkes et al. (2014). mbsf = metres below seafloor.

## Slide 3
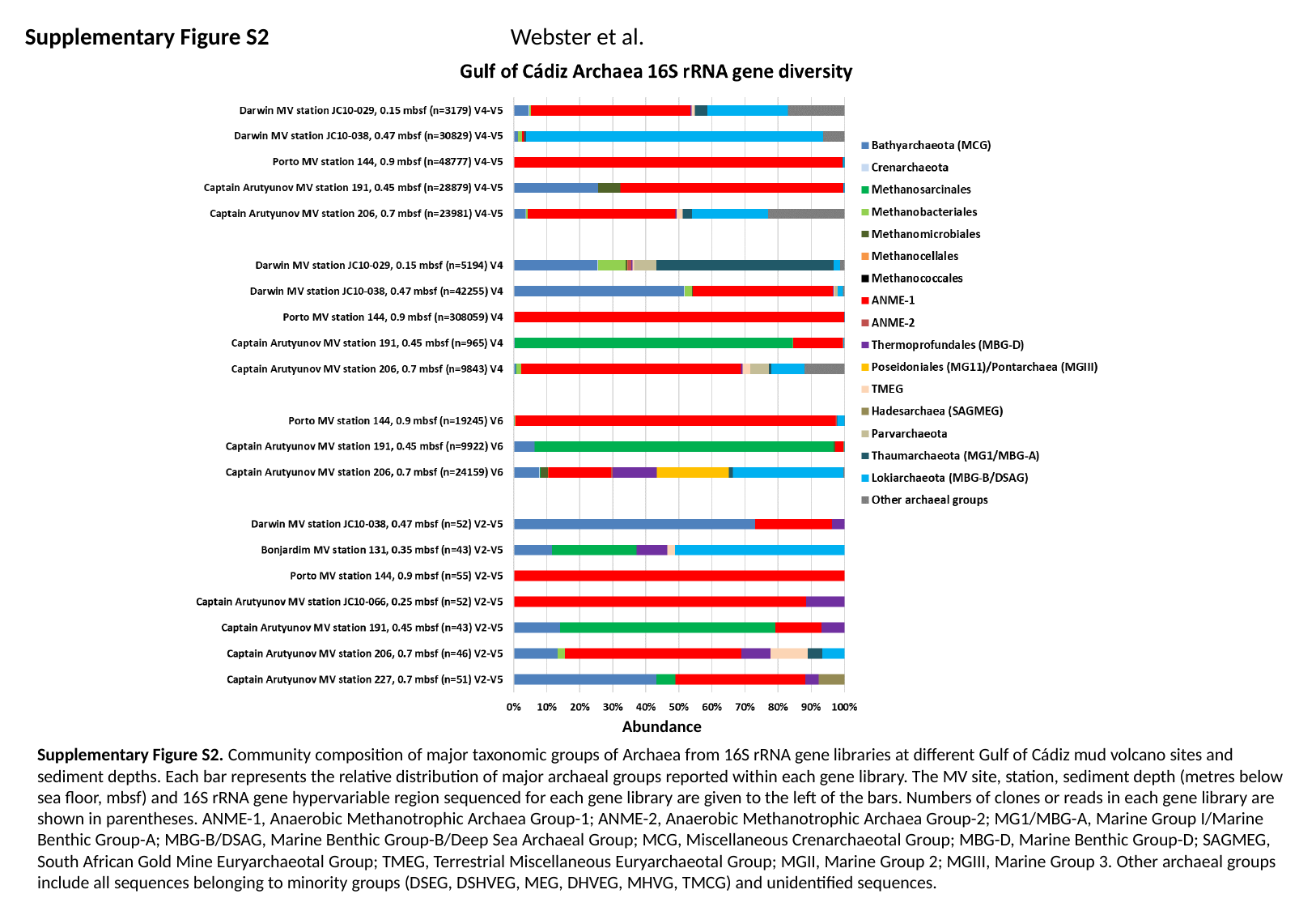

Supplementary Figure S2		Webster et al.
Abundance
Supplementary Figure S2. Community composition of major taxonomic groups of Archaea from 16S rRNA gene libraries at different Gulf of Cádiz mud volcano sites and sediment depths. Each bar represents the relative distribution of major archaeal groups reported within each gene library. The MV site, station, sediment depth (metres below sea floor, mbsf) and 16S rRNA gene hypervariable region sequenced for each gene library are given to the left of the bars. Numbers of clones or reads in each gene library are shown in parentheses. ANME-1, Anaerobic Methanotrophic Archaea Group-1; ANME-2, Anaerobic Methanotrophic Archaea Group-2; MG1/MBG-A, Marine Group I/Marine Benthic Group-A; MBG-B/DSAG, Marine Benthic Group-B/Deep Sea Archaeal Group; MCG, Miscellaneous Crenarchaeotal Group; MBG-D, Marine Benthic Group-D; SAGMEG, South African Gold Mine Euryarchaeotal Group; TMEG, Terrestrial Miscellaneous Euryarchaeotal Group; MGII, Marine Group 2; MGIII, Marine Group 3. Other archaeal groups include all sequences belonging to minority groups (DSEG, DSHVEG, MEG, DHVEG, MHVG, TMCG) and unidentified sequences.

## Slide 4
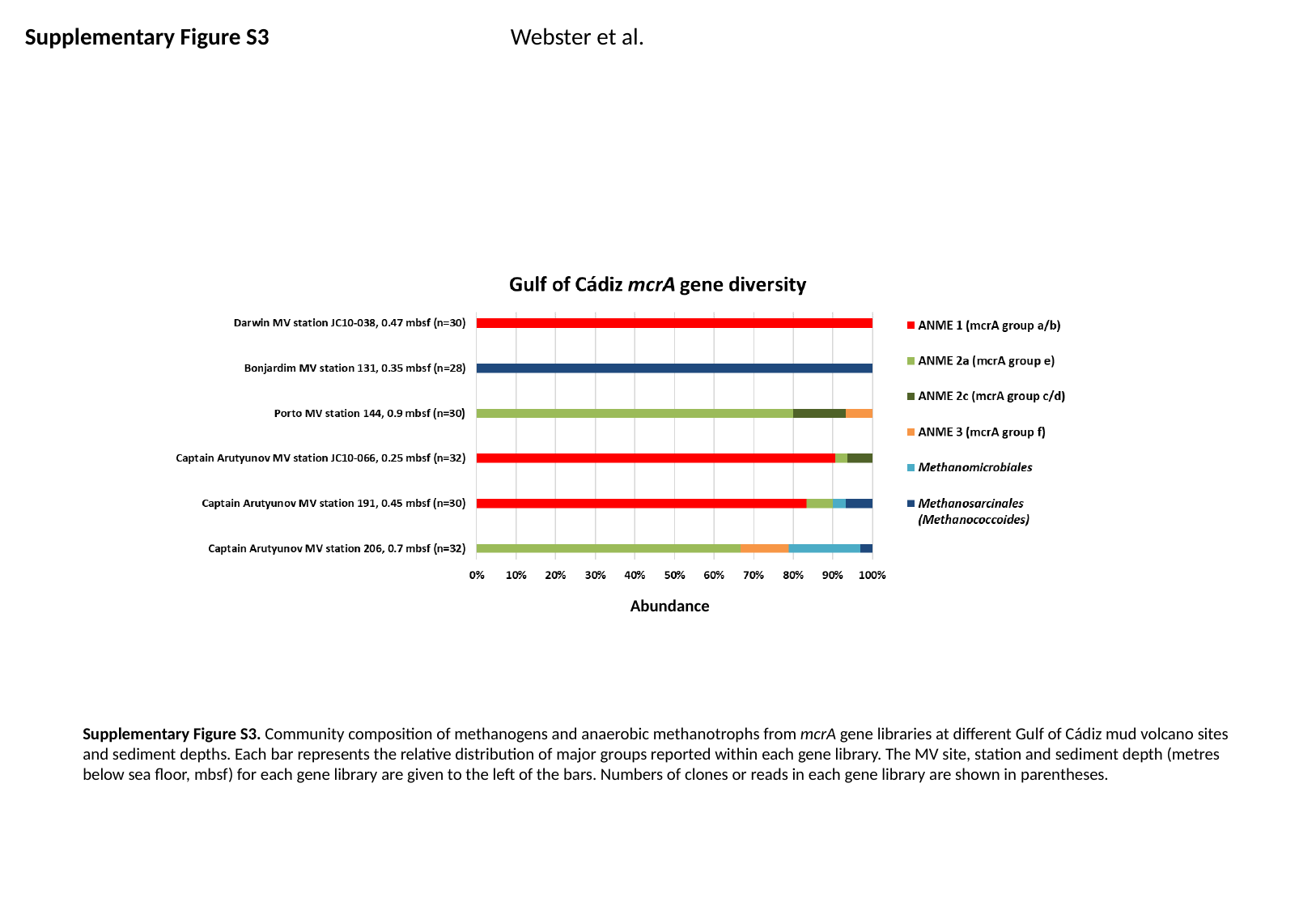

Supplementary Figure S3		Webster et al.
Abundance
Supplementary Figure S3. Community composition of methanogens and anaerobic methanotrophs from mcrA gene libraries at different Gulf of Cádiz mud volcano sites and sediment depths. Each bar represents the relative distribution of major groups reported within each gene library. The MV site, station and sediment depth (metres below sea floor, mbsf) for each gene library are given to the left of the bars. Numbers of clones or reads in each gene library are shown in parentheses.

## Slide 5
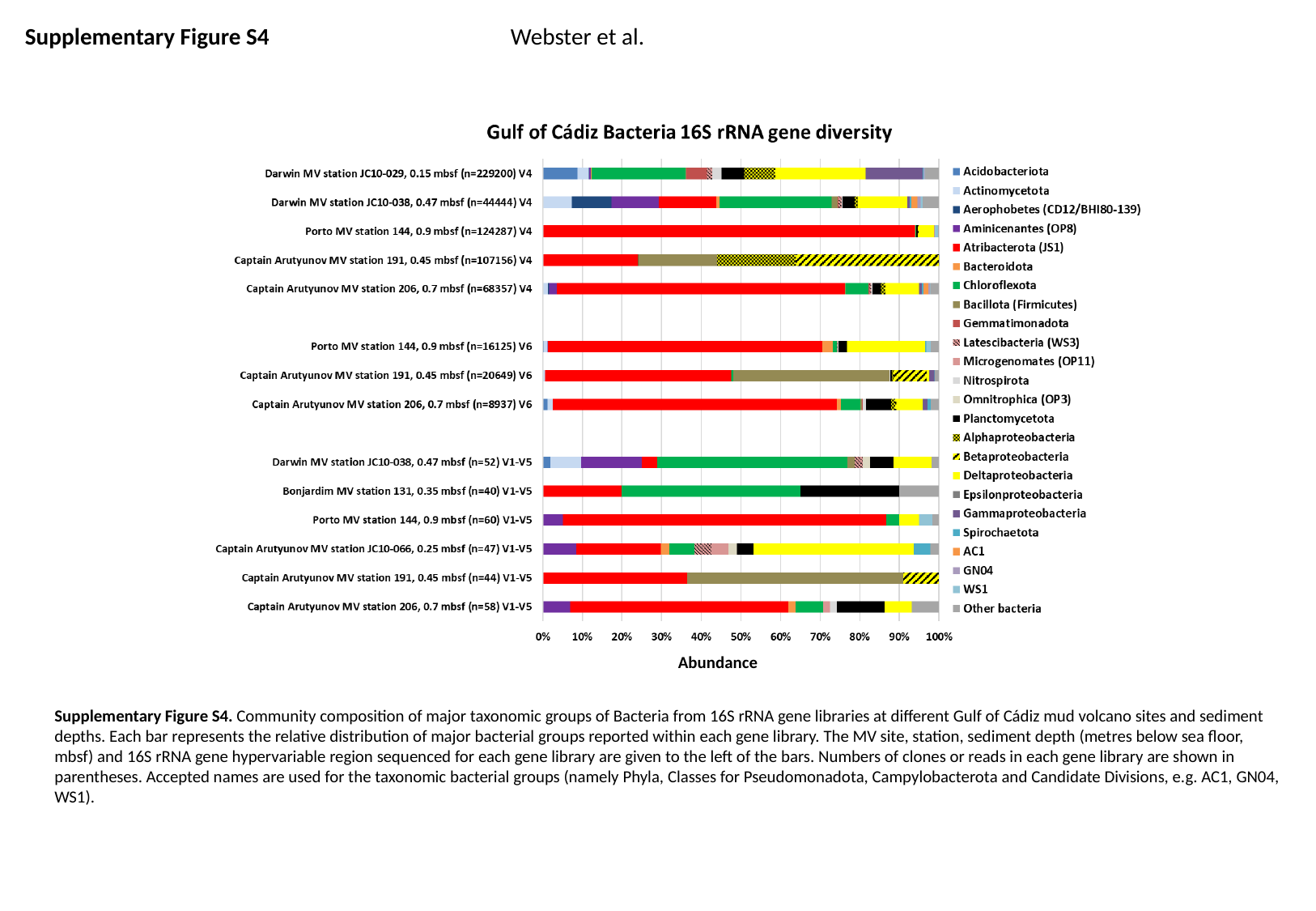

Supplementary Figure S4		Webster et al.
Abundance
Supplementary Figure S4. Community composition of major taxonomic groups of Bacteria from 16S rRNA gene libraries at different Gulf of Cádiz mud volcano sites and sediment depths. Each bar represents the relative distribution of major bacterial groups reported within each gene library. The MV site, station, sediment depth (metres below sea floor, mbsf) and 16S rRNA gene hypervariable region sequenced for each gene library are given to the left of the bars. Numbers of clones or reads in each gene library are shown in parentheses. Accepted names are used for the taxonomic bacterial groups (namely Phyla, Classes for Pseudomonadota, Campylobacterota and Candidate Divisions, e.g. AC1, GN04, WS1).

## Slide 6
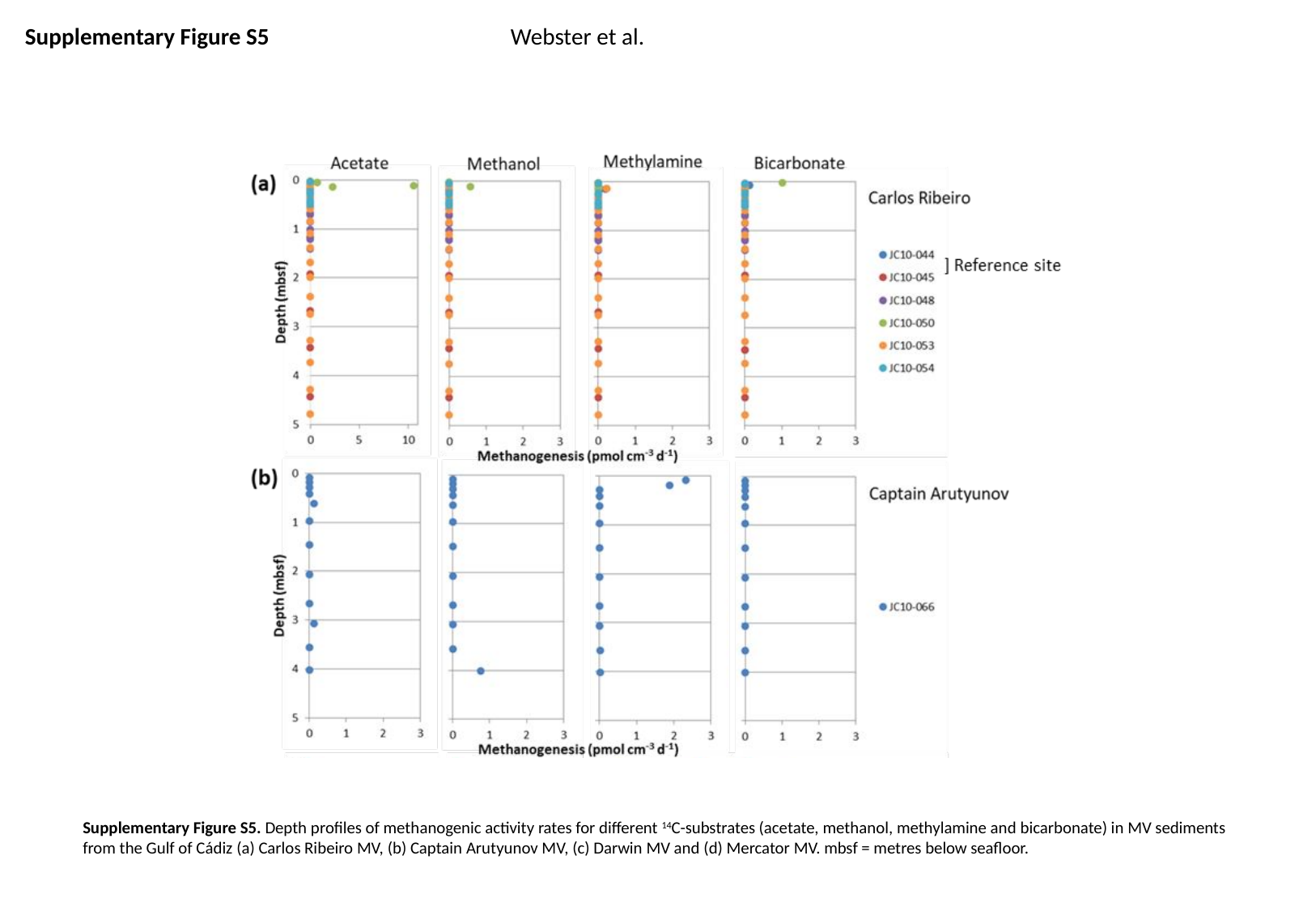

Supplementary Figure S5		Webster et al.
Supplementary Figure S5. Depth profiles of methanogenic activity rates for different 14C-substrates (acetate, methanol, methylamine and bicarbonate) in MV sediments from the Gulf of Cádiz (a) Carlos Ribeiro MV, (b) Captain Arutyunov MV, (c) Darwin MV and (d) Mercator MV. mbsf = metres below seafloor.

## Slide 7
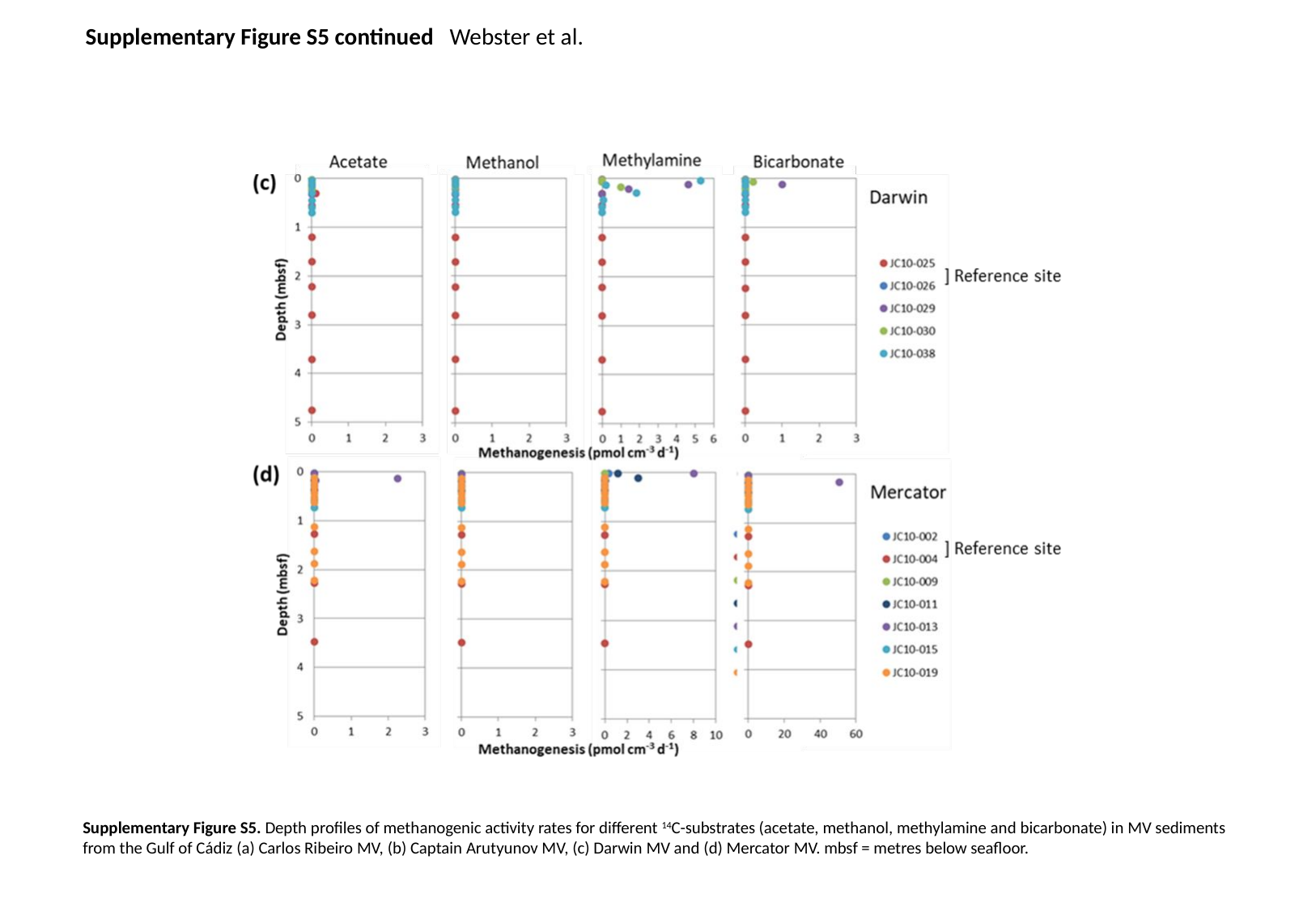

Supplementary Figure S5 continued	Webster et al.
Supplementary Figure S5. Depth profiles of methanogenic activity rates for different 14C-substrates (acetate, methanol, methylamine and bicarbonate) in MV sediments from the Gulf of Cádiz (a) Carlos Ribeiro MV, (b) Captain Arutyunov MV, (c) Darwin MV and (d) Mercator MV. mbsf = metres below seafloor.

## Slide 8
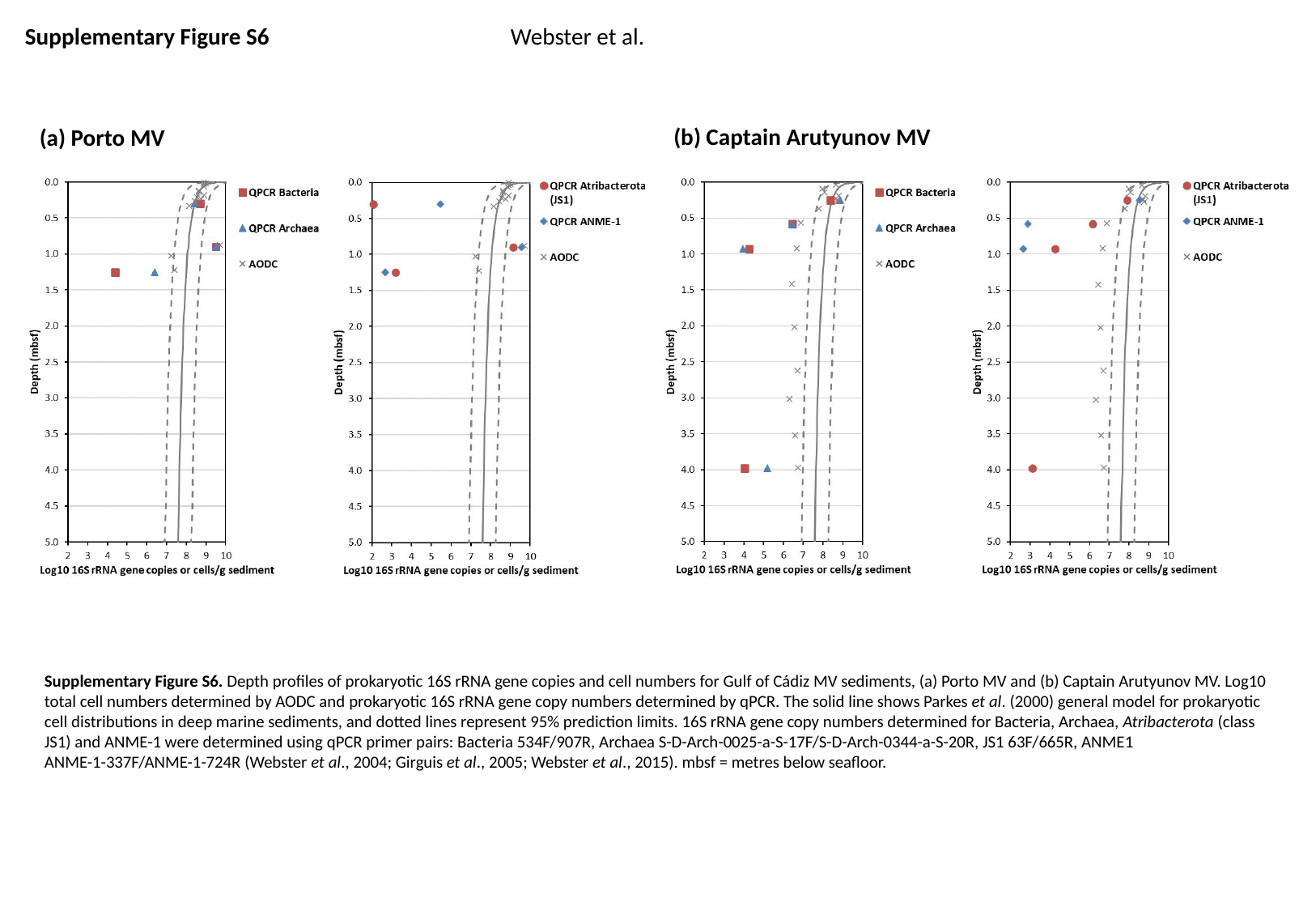

Supplementary Figure S6		Webster et al.
(b) Captain Arutyunov MV
(a) Porto MV
Supplementary Figure S6. Depth profiles of prokaryotic 16S rRNA gene copies and cell numbers for Gulf of Cádiz MV sediments, (a) Porto MV and (b) Captain Arutyunov MV. Log10 total cell numbers determined by AODC and prokaryotic 16S rRNA gene copy numbers determined by qPCR. The solid line shows Parkes et al. (2000) general model for prokaryotic cell distributions in deep marine sediments, and dotted lines represent 95% prediction limits. 16S rRNA gene copy numbers determined for Bacteria, Archaea, Atribacterota (class JS1) and ANME-1 were determined using qPCR primer pairs: Bacteria 534F/907R, Archaea S-D-Arch-0025-a-S-17F/S-D-Arch-0344-a-S-20R, JS1 63F/665R, ANME1 ANME-1-337F/ANME-1-724R (Webster et al., 2004; Girguis et al., 2005; Webster et al., 2015). mbsf = metres below seafloor.

## Slide 9
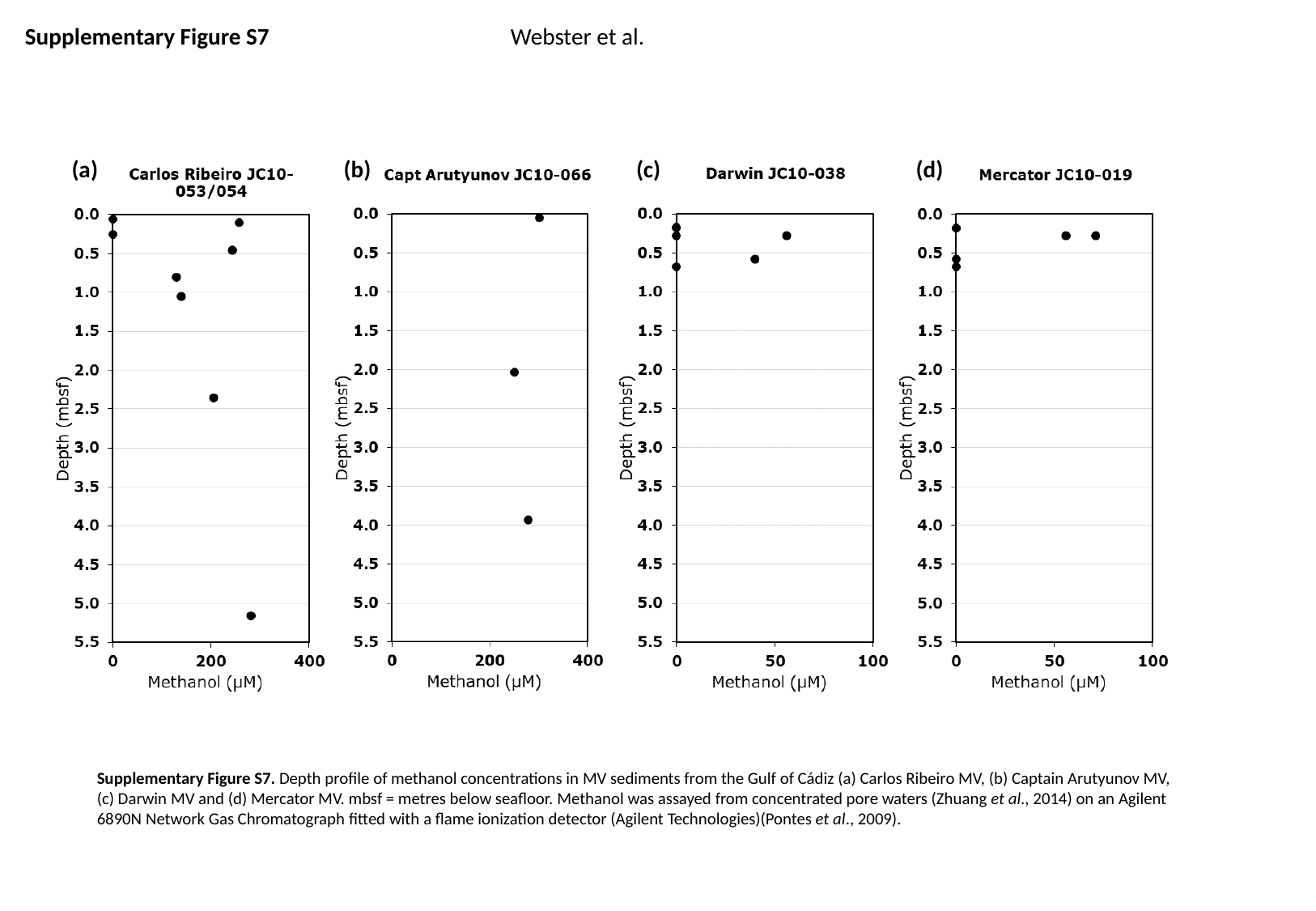

Supplementary Figure S7		Webster et al.
(a)
(c)
(d)
(b)
Supplementary Figure S7. Depth profile of methanol concentrations in MV sediments from the Gulf of Cádiz (a) Carlos Ribeiro MV, (b) Captain Arutyunov MV, (c) Darwin MV and (d) Mercator MV. mbsf = metres below seafloor. Methanol was assayed from concentrated pore waters (Zhuang et al., 2014) on an Agilent 6890N Network Gas Chromatograph fitted with a flame ionization detector (Agilent Technologies)(Pontes et al., 2009).

## Slide 10
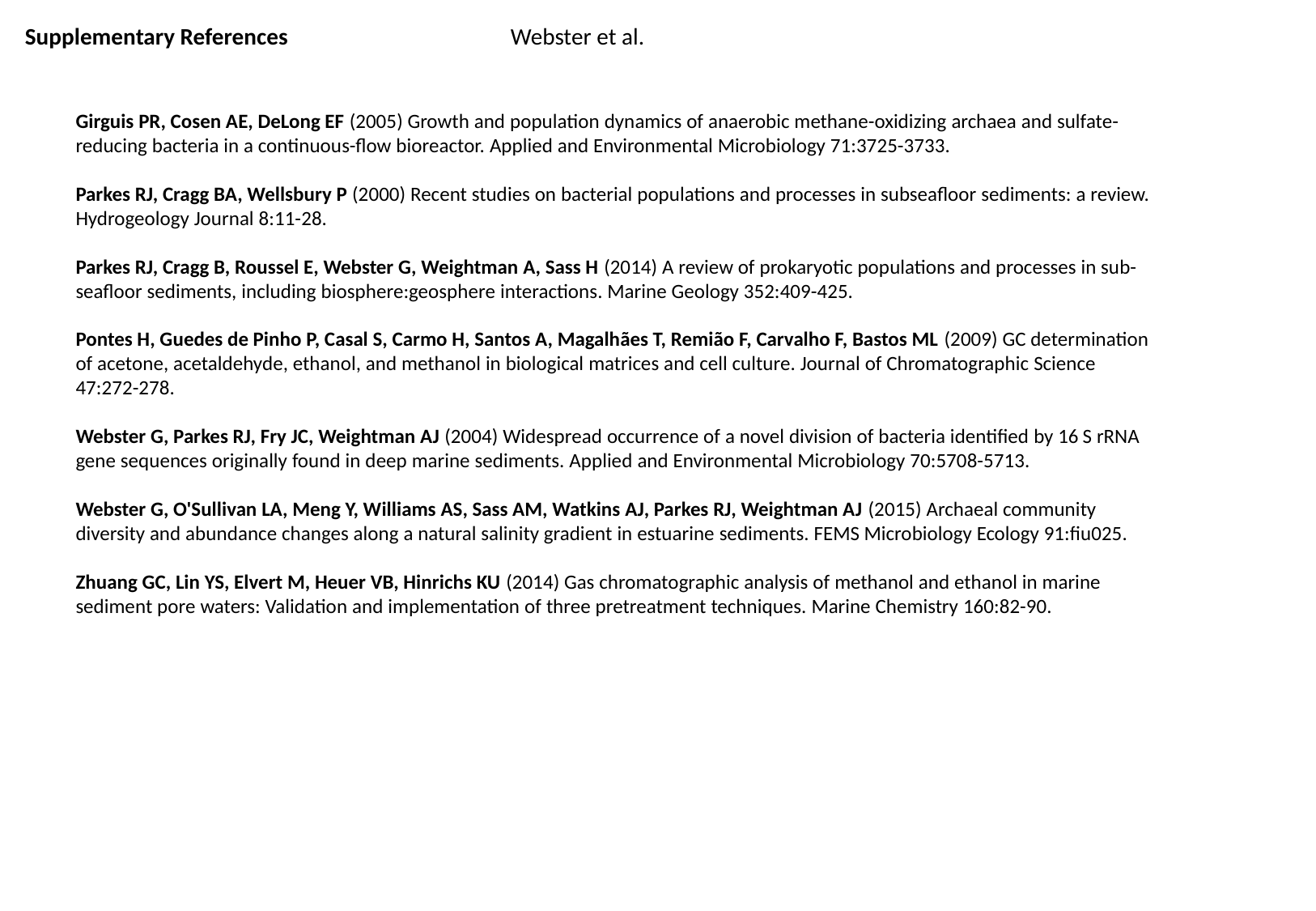

Supplementary References		Webster et al.
Girguis PR, Cosen AE, DeLong EF (2005) Growth and population dynamics of anaerobic methane-oxidizing archaea and sulfate-reducing bacteria in a continuous-flow bioreactor. Applied and Environmental Microbiology 71:3725-3733.
Parkes RJ, Cragg BA, Wellsbury P (2000) Recent studies on bacterial populations and processes in subseafloor sediments: a review. Hydrogeology Journal 8:11-28.
Parkes RJ, Cragg B, Roussel E, Webster G, Weightman A, Sass H (2014) A review of prokaryotic populations and processes in sub-seafloor sediments, including biosphere:geosphere interactions. Marine Geology 352:409-425.
Pontes H, Guedes de Pinho P, Casal S, Carmo H, Santos A, Magalhães T, Remião F, Carvalho F, Bastos ML (2009) GC determination of acetone, acetaldehyde, ethanol, and methanol in biological matrices and cell culture. Journal of Chromatographic Science 47:272-278.
Webster G, Parkes RJ, Fry JC, Weightman AJ (2004) Widespread occurrence of a novel division of bacteria identified by 16 S rRNA gene sequences originally found in deep marine sediments. Applied and Environmental Microbiology 70:5708-5713.
Webster G, O'Sullivan LA, Meng Y, Williams AS, Sass AM, Watkins AJ, Parkes RJ, Weightman AJ (2015) Archaeal community diversity and abundance changes along a natural salinity gradient in estuarine sediments. FEMS Microbiology Ecology 91:fiu025.
Zhuang GC, Lin YS, Elvert M, Heuer VB, Hinrichs KU (2014) Gas chromatographic analysis of methanol and ethanol in marine sediment pore waters: Validation and implementation of three pretreatment techniques. Marine Chemistry 160:82-90.
