# Supplementary material for: Methanogen activity and microbial diversity in Gulf of Cádiz mud volcano sediments
Source: Front Microbiol. 2023 May 24;14:1157337. doi: 10.3389/fmicb.2023.1157337 (PMC10244519; doi:10.3389/fmicb.2023.1157337)
Supplement: Supplementary file 2 [file Table_1.DOCX]

**Supplementary Table S1.** PCR amplification and total cell numbers of different sediment samples from the Gulf of Cádiz mud volcanoes.

| **Mud volcano** | **Station** | **Sediment depth (mbsf)** | **Direct PCR amplification^a^** | **Cell number/cm^3^ sediment (AODC)^b^** |
| --- | --- | --- | --- | --- |
| Porto | 144 | 0.90 | + | 5.11 x10^9^ |
| Bonjardim | 131 | 0.35 | + | 1.19 x10^8^ |
|  |  | 1.25 | - | 4.49 x10^6^ |
|  |  | 1.60 | - | 4.36 x10^6^ |
|  |  | 2.45 | - | 1.98 x10^6^ |
|  |  | 3.10 | - | 1.62 x10^6^ |
| Carlos Ribeiro | 053 | 0.10 | - | 5.58 x10^7^ |
|  |  | 0.30 | - | 7.26 x10^6^ |
|  |  | 1.35 | - | 3.64 x10^6^ |
|  |  | 5.15 | - | 3.10 x10^6^ |
| Captain Arutyunov | 191 | 0.45 | + | 3.57 x10^7^ |
|  |  | 1.70 | - | 2.37 x10^6^ |
|  |  | 2.60 | - | 1.57 x10^6^ |
|  | 206 | 0.70 | + | 6.12 x10^7^ |
|  |  | 1.05 | - | 3.28 x10^6^ |
|  |  | 2.75 | - | 1.35 x10^6^ |
|  | 227 | 0.70 | + | 7.10 x10^6^ |
|  |  | 1.15 | - | 2.71 x10^6^ |
|  | 066 | 0.25 | + | 5.14 x10^8^ |
|  |  | 0.58 | - | 7.58 x10^6^ |
|  |  | 0.93 | - | 5.46 x10^6^ |
|  |  | 3.98 | - | 4.69 x10^6^ |
| Darwin | 029 | 0.15 | + | 4.08 x10^8^ |
|  | 038 | 0.47 | + | 9.71 x10^7^ |
| Meknes | 306 | 1.20 | - | 3.16 x10^6^ |
| Mercator | 238 | 0.85 | - | 2.31 x10^6^ |
|  |  | 1.65 | - | 1.91 x10^6^ |
|  |  | 2.05 | - | 1.53 x10^6^ |
|  | 019 | 0.20 | - | 5.54 x10^7^ |
|  |  | 0.40 | - | 1.36 x10^7^ |

^a^Direct PCR with bacterial or archaeal 16S rRNA gene primers.

^b^AODC = acridine orange direct cell count.
