# Supplementary material for: Methanogen activity and microbial diversity in Gulf of Cádiz mud volcano sediments
Source: Front Microbiol. 2023 May 24;14:1157337. doi: 10.3389/fmicb.2023.1157337 (PMC10244519; doi:10.3389/fmicb.2023.1157337)
Supplement: Supplementary file 3 [file Table_2.DOCX]

**Supplementary Table S2.** Rates of methanogenesis (pmol cm^-3^ d^-1^) for Gulf of Cádiz mud volcanoes incubated with four different ^14^C-methanogenic substrates.

| **Mud volcano** | **Stations^a^** | **Average rate of methanogenesis^b^ (% total methanogenesis)** **pmol cm^-3^ d^-1^** | | | | |
| --- | --- | --- | --- | --- | --- | --- |
|  |  | **Acetate** | **Bicarbonate** | **Methanol** | **Methylamine** | **Total** |
| Carlos Ribeiro | Ref 044/045 | 0 | 0.012 (92.3%) | 0 | 0.001 (7.7%) | 0.013 (100%) |
|  | 048/050/053/054 | 0.368 (86.6%) | 0.028 (6.6%) | 0.016 (3.8%) | 0.013 (3.0%) | 0.425 (100%) |
| Captain Arutyunov | 066 | 0.021 (4.7%) | 0 | 0.064 (14.5%) | 0.357 (80.8%) | 0.442 (100%) |
| Darwin | Ref 025/026 | 0.010 (100%) | 0 | 0 | 0 | 0.010 (100%) |
|  | 029/030/038 | 0 | 0.087 (7.2%) | 0 | 1.121 (92.8%) | 1.208 (100%) |
| Mercator | Ref 002/004 | 0 | 0 | 0 | 0.070 (100%) | 0.070 (100%) |
|  | 009/011/013/015/019 | 0.072 (3.5%) | 1.579 (77.6%) | 0 | 0.385 (18.9%) | 2.036 (100%) |
| **Combined average methanogenesis** | **Reference station** | **33.3%** | **30.8%** | **0%** | **35.9%** | **100%** |
|  | **MV** | **23.7%** | **22.8%** | **4.6%** | **48.9%** | **100%** |

^a^Ref = reference stations for each respective mud volcano (MV) site.

^b^Average rate of methanogenesis (pmol cm^-3^ d^-1^) calculated from data collected at several sampling stations and sediment depths for each MV site.

See supplementary Figure 1 for complete data set.
