# Supplementary material for: Methanogen activity and microbial diversity in Gulf of Cádiz mud volcano sediments
Source: Front Microbiol. 2023 May 24;14:1157337. doi: 10.3389/fmicb.2023.1157337 (PMC10244519; doi:10.3389/fmicb.2023.1157337)
Supplement: Supplementary file 4 [file Table_3.DOCX]

**Supplementary Table S3.** Archaeal *mcrA* and 16S rRNA gene identification based on nucleotide BLAST search of sequences retrieved by PCR-DGGE from Gulf of Cádiz mud volcano sediment methanogen enrichments.

| **MV enrichment (station, sediment depth)** | **Substrate added** | ***mcrA* gene** | | | **16S rRNA gene** | | |
| --- | --- | --- | --- | --- | --- | --- | --- |
|  |  | **Nearest match (accession #)**  **% Similarity** | **Nearest cultured relative (accession #) % Similarity** | **Phylogenetic group** | **Nearest match (accession #)**  **% Similarity** | **Nearest cultured relative (accession #) % Similarity** | **Phylogenetic group** |
| **Porto**  **(144, 0.77 m)** | Benzoate | Napoli MV sediment clone G04b (HQ454464)  100% (448/448) | - | ANME-2a  (*mcrA* group e) | Amsterdam MV sediment clone AMSMV-25-A21 (HQ588680)  98% (143/146) | - | ANME-1 |
|  |  | Eel River Basin sediment clone so4M71 (FJ264880)  96% (431/446) | - | ANME-2c  (*mcrA* group c/d) | Aarhus Bay sediment clone ArchSMTZ_1B (FR695328) 98% (143/146) | - | ANME-1 |
| **Porto**  **(144, 0.77 m)** | Methylamine | Eel River Basin sediment clone so4M71 (FJ264880)  96% (441/460) | - | ANME-2c  (*mcrA* group c/d) | Aarhus Bay sediment clone ArchSMTZ_1B (FR695328) 98% (143/146) | - | ANME-1 |
| **Bonjardim**  (131, 0.42 m) | Methanol | Guaymas Basin sediment clone B09 (AY837774)  98% (467/476) | *Methanococcoides alaskense*  DSM17273 (AB353221)  96% (455/475) | *Methanosarcinales* | Sagami Bay sediment clone: SBC12G405A195 (AB561824)  99% (149/150) | *Methanococcoides alaskense*  DSM17273 (NR_029122)  99% (149/150) | *Methanosarcinales* |
| **Bonjardim**  (131, 0.42m) | Methylamine | Guaymas Basin sediment clone B09 (AY837774)  99% (472/478) | *Methanococcoides alaskense*  DSM17273 (AB353221)  96% (461/478) | *Methanosarcinales* | Sagami Bay sediment clone: SBC12G405A195 (AB561824)  99% (149/150) | *Methanococcoides alaskense*  DSM17273 (NR_029122)  99% (149/150) | *Methanosarcinales* |
| **Bonjardim**  (131, 1.67 m) | Methanol | - | *-* | n.a. | Sagami Bay sediment clone: SBC12G405A195 (AB561824)  99% (149/150) | *Methanococcoides alaskense*  DSM17273 (NR_029122)  99% (149/150) | *Methanosarcinales* |
| **Captain Arutyunov**  (191, 0.52 m) | Methanol | Marennes-Oleron Bay sediment clone MOBOcr43012 (AM942080)  97% (468/482) | *Methanococcoides methylutens* (U22235)  97% (466/482) | *Methanosarcinales* | Amsterdam MV sediment clone AMSMV-5-A48  (HQ588654)  99% (150/151) | *Methanococcoides methylutens* DSM2657 (FR733669)  99% (150/151) | *Methanosarcinales* |
| **Captain Arutyunov**  (191, 0.52 m) | Methylamine | Marennes-Oleron Bay sediment clone MOBOcr43012 (AM942080)  97% (465/479) | *Methanococcoides methylutens* (U22235)  97% (456/469) | *Methanosarcinales* | Amsterdam MV sediment clone AMSMV-5-A48  (HQ588654)  99% (150/151) | *Methanococcoides methylutens* DSM2657 (FR733669)  99% (150/151) | *Methanosarcinales* |
| **Captain Arutyunov**  (191, 0.52 m) | H_2_/CO_2_ | - | *-* | n.a. | - | *-* | n.a. |
| **Captain Arutyunov**  (191, 1.77 m) | Methylamine | Marennes-Oleron Bay sediment clone MOBOcr43012 (AM942080)  97% (465/479) | *Methanococcoides methylutens* (U22235)  97% (456/469) | *Methanosarcinales* | Amsterdam MV sediment clone AMSMV-5-A48  (HQ588654)  99% (150/151) | *Methanococcoides methylutens* DSM2657 (FR733669)  99% (150/151) | *Methanosarcinales* |
| **Captain Arutyunov**  (206, 0.77 m) | Methylamine | Cascadia Margin sediment clone 1327C21-7 (AB525709)  98% (471/481) | *Methanococcoides alaskense* DSM17273 (AB353221)  96% (464/481) | *Methanosarcinales* | Amsterdam MV sediment clone AMSMV-5-A48  (HQ588654)  99% (150/151) | *Methanococcoides methylutens* DSM2657 (FR733669)  99% (150/151) | *Methanosarcinales* |
| **Captain Arutyunov**  (206, 2.22 m) | Methylamine | Napoli MV sediment clone NapMat-2_4-mcrD02 (HM004837)  95% (451/476) | *-* | ANME-2a  (*mcrA* group e) | - | - | n.a. |
| **Captain Arutyunov**  (227, 0.77 m) | Methanol | - | *-* | n.a. | Sagami Bay sediment clone: SBC12G405A195 (AB561824)  99% (149/150) | *Methanococcoides alaskense*  DSM17273 (NR_029122)  99% (149/150) | *Methanosarcinales* |
| **Captain Arutyunov**  (227, 0.77 m) | Methylamine | Cascadia Margin sediment clone 1327C22-4 (AB525712)  98% (470/481) | *Methanococcoides methylutens* (U22235)  97% (454/467) | *Methanosarcinales* | Sagami Bay sediment clone: SBC12G405A195 (AB561824)  99% (149/150) | *Methanococcoides alaskense*  DSM17273 (NR_029122)  99% (149/150) | *Methanosarcinales* |
| **Captain Arutyunov**  (227, 1.77 m) | Methanol | - | *-* | n.a. | Sagami Bay sediment clone: SBC12G405A195 (AB561824)  99% (149/150) | *Methanococcoides alaskense*  DSM17273 (NR_029122)  99% (149/150) | *Methanosarcinales* |
| **Captain Arutyunov**  (227, 1.77 m) | Methylamine | Guaymas Basin sediment clone 4486_MCRird_d245  98% (454/465) | *Methanococcoides methylutens* (U22235)  98% (454/465) | *Methanosarcinales* | Amsterdam MV sediment clone AMSMV-5-A48  (HQ588654)  99% (150/151) | *Methanococcoides methylutens* DSM2657 (FR733669)  99% (150/151) | *Methanosarcinales* |
| **Darwin**  (036, 0.11 m) | Methanol | Marennes-Oleron Bay sediment clone MOBOcr43012 AM942080 99% (443/448) | *Methanococcoides methylutens* (U22235)  100% (435/435) | *Methanosarcinales* | Loch Creran sediment clone TAE10 (HQ849068)  92% (139/151) | *Methanococcoides* sp. MO-MCD (AB598271)  91% (138/151) | *Methanosarcinales* |
| **Darwin**  (036, 0.11 m) | Methylamine | Marennes-Oleron Bay sediment clone MOBOcr43012 (AM942080)  99% (443/448) | *Methanococcoides methylutens* (U22235)  100% (435/435) | *Methanosarcinales* | Capt Arutyunov MV sediment clone CpA ArcA33 (FN397890)  97% (146/150) | *Methanococcoides alaskense* AK-9 (AY941802)  97% (146/150) | *Methanosarcinales* |
| **Meknes**  (306, 0.77 m) | Acetate | - | *-* | n.a. | - | *-* | n.a. |
| **Meknes**  (306, 0.77 m) | Benzoate | - | *-* | n.a. | - | *-* | n.a. |
| **Meknes**  (306, 0.77 m) | Hexadecane | - | *-* | n.a. | - | *-* | n.a. |
| **Meknes**  (306, 0.77 m) | Methanol | Marennes-Oleron Bay sediment clone MOBOcr43977 (AM942096)  97% (468/481) | *Methanococcoides burtonii* DSM6242 (CP000300)  97% (466/482) | *Methanosarcinales* | Amsterdam MV sediment clone AMSMV-5-A48  (HQ588654)  99% (150/151) | *Methanococcoides methylutens* DSM2657 (FR733669)  99% (150/151) | *Methanosarcinales* |
| **Meknes**  (306, 0.77 m) | Methylamine | Marennes-Oleron Bay sediment clone MOBOcr43012 (AM942080)  97% (465/479) | *Methanococcoides burtonii* DSM6242 (CP000300)  97% (466/480) | *Methanosarcinales* | Amsterdam MV sediment clone AMSMV-5-A48  (HQ588654)  99% (150/151) | *Methanococcoides methylutens* DSM2657 (FR733669)  99% (150/151) | *Methanosarcinales* |
| **Meknes**  (306, 1.82 m) | Acetate | - | *-* | n.a. | - | *-* | n.a. |
| **Mekne**s  (306, 1.82 m) | Methylamine | - | *-* | n.a. | Amsterdam MV sediment clone AMSMV-5-A48  (HQ588654)  99% (150/151) | *Methanococcoides methylutens* DSM2657 (FR733669)  99% (150/151) | *Methanosarcinales* |
| **Mercator**  (238, 0.72 m) | Methylamine | Marennes-Oleron Bay sediment clone MOBOcr43977 (AM942096)  97% (465/479) | *Methanococcoides burtonii* DSM6242 (CP000300)  97% (465/480) | *Methanosarcinales* | Amsterdam MV sediment clone AMSMV-5-A48  (HQ588654)  99% (150/151) | *Methanococcoides methylutens* DSM2657 (FR733669)  99% (150/151) | n.a. |
| **Mercator**  (009, 0.13 m) | Hexadecane | - | *-* | n.a. | - | *-* | n.a. |
| **Mercator**  (013, 0.15 m) | Acetate | Napoli MV sediment clone F01b (HQ454458)  98% (462/470) | *-* | ANME-2a  (*mcrA* group e) | - | - | n.a. |
| **Mercator**  (013, 0.15 m) | Benzoate | Napoli MV sediment clone F01b (HQ454458)  98% (462/470) | *-* | ANME-2a  (*mcrA* group e) | - | - | n.a. |
| **Mercator**  (013, 0.15 m) | Hexadecane | Napoli MV sediment clone G04b (HQ454464)  97% (451/466) | - | ANME-2a  (*mcrA* group e) | Amsterdam MV sediment clone AMSMV-25-A21 (HQ588680)  98% (143/146) | - | ANME-1 |
| **Mercator**  (015, 0.30 m) | Methanol | - | *-* | n.a. | Amsterdam MV sediment clone AMSMV-5-A48 (HQ588654) 100% (151/151) | *Methanococcoides methylutens* DSM2657 (FR733669)  100% (151/151) | *Methanosarcinales* |
| **Mercator**  (019, 0.18 m) | Acetate | - | *-* | n.a. | - | *-* | n.a. |
| **Mercator**  (019, 1.90 m) | Benzoate | - | *-* | n.a. | - | *-* | n.a. |
| **Mercator**  (019, 1.90 m) | Hexadecane | Peru Margin sediment clone ME86-A11 (AJ867762)  99% (405/409) | *Methanobrevibacter arboriphilus* (AB300777)  100% (402/402) | *Methanobacteriales* | - | *-* | n.a. |
| **Mercator**  (019, 2.23 m) | Hexadecane | Peru Margin sediment clone ME86-A11 (AJ867762)  99% (408/409) | *Methanobrevibacter arboriphilus* (AB300777)  100% (402/402) | *Methanobacteriales* | - | *-* | n.a. |

n.a. = no PCR amplification for this sediment slurry

See Figure 4 and Table 2 for further information.
