# Supplementary material for: Methanogen activity and microbial diversity in Gulf of Cádiz mud volcano sediments
Source: Front Microbiol. 2023 May 24;14:1157337. doi: 10.3389/fmicb.2023.1157337 (PMC10244519; doi:10.3389/fmicb.2023.1157337)
Supplement: Supplementary file 5 [file Table_4.DOCX]

**Supplementary Table S4.** Bacterial 16S rRNA gene identification based on nucleotide BLAST search of sequences retrieved by PCR-DGGE from Gulf of Cádiz mud volcano sediment methanogen enrichments.

| **MV enrichment (station, sediment depth)** | **Substrate added** | **DGGE bands identified/total number of DGGE bands** | **Band number** | **16S rRNA gene** | | |
| --- | --- | --- | --- | --- | --- | --- |
|  |  |  |  | **Nearest match (accession #)**  **% Similarity** | **Nearest cultured relative (accession #)**  **% Similarity (>90%)** | **Phylogenetic group** |
| **Porto**  (144, 0.77 m) | Benzoate | 5/6 | 78a | Porto MV sediment DGGE band 4.9-1 (FN547399)  97% (190/196) | - | JS1 |
|  |  |  | 55 | Porto MV sediment DGGE band 4.9-1 (FN547399)  99% (195/196) | - | JS1 |
|  |  |  | 71b | Wadden Sea bacterium N5IX (AJ786047)  97% (165/170) | *Acidaminobacter* sp. CJ5 (GU570195)  92% (157/171) | *Firmicutes* |
|  |  |  | 56 | Japan Sea sediment clone UT06_10_52B_36 (AB525452) 99% (193/195) | *Desulfobacterium indolicum* strain In04 (NR_028897)  94% (184/195) | *Deltaproteobacteria* |
|  |  |  | 57 | Japan Sea sediment clone UT06_10_52B_53 (AB525463) 98% (166/169) | - | Novel group |
| **Porto**  (144, 0.77 m) | Methylamine | 6/7 | 78 | Porto MV sediment DGGE band 4.9-1 (FN547399)  97% (190/196) | - | JS1 |
|  |  |  | 79 | Sea of Okhotsk sediment clone OHKB6.89 (AB094880)  99% (194/196) | - | JS1 |
|  |  |  | 71a | Wadden Sea bacterium N5IX (AJ786047)  97% (165/170) | *Acidaminobacter* sp. CJ5 (GU570195)  92% (157/171) | *Firmicutes* |
|  |  |  | 80 | Japan Sea sediment clone UT06_10_52B_36 (AB525452) 98% (192/195) | *Desulfobacterium indolicum* strain In04 (NR_028897)  94% (183/195) | *Deltaproteobacteria* |
|  |  |  | 76a | Lignite mine sediment clone MEBA65 (JF973656)  100% (194/194) | *Burkholderia phenazinium* strain 1S1 (AJ574888)  100% (194/194) | *Betaproteobacteria* |
|  |  |  | 81 | Japan Sea sediment clone UT06_10_52B_53 (AB525463) 98% (166/169) | - | Novel group |
| **Bonjardim**  (131, 0.42 m) | Methanol | 6/8 | 39 | *Pseudomonas* sp. B103T-5 (HE664166) 100% (194/194) | *Pseudomonas reinekei* MT1 (AM2935651)  100% (194/194) | *Gammaproteobacteria* |
|  |  |  | 40a | Angola Basin sediment clone Ulr1500 (AM997465)  99% (168/170) | *Dehalococcoides ethenogenes* 195 (CP000027)  90% (153/170) | *Chloroflexi* |
|  |  |  | 41 | Sponge-associated clone XC2A09 (JN596681)  98% (166/170) | *Dehalogenimonas lykanthroporepellens* BL-DC-9 (CP002084)  91% (154/170) | *Chloroflexi* |
|  |  |  | 42a | Napoli MV sediment clone Napoli-3B-22 (AY592698)  99% (169/170) | *Dehalogenimonas lykanthroporepellens* BL-DC-9 (CP002084)  95% (161/170) | *Chloroflexi* |
|  |  |  | 44a | Tetrachloroethene enrichment clone 635_E16_PCE (FM178817) 100% (177/177) | *Corynebacterium kroppenstedtii* DSM 44385 (CP001620)  100% (177/177) | *Actinobacteria* |
|  |  |  | 45 | Activated sludge clone T1-70 (JF502914)  95% (185/194) | *Marinimicrobium koreense strain M9 (AY839869)*  *91% (176/194)* | *Gammaproteobacteria* |
| **Bonjardim**  (131, 0.42 m) | Methylamine | 7/12 | 64a | Amsterdam MV sediment clone AMSMV-30-B5 (HQ588613) 97% (190/195) | - | JS1 |
|  |  |  | 47a | Microbial fuel cell clone anode_Ac18 (JQ654818)  98% (190/194) | *Alcaligenes aquatilis* LMG22996 (AJ937889)  98% (190/194) | *Betaproteobacteria* |
|  |  |  | 48a | Kazan MV sediment clone KZNMV-30-B24 (FJ712605) 100% (195/195) | - | JS1 |
|  |  |  | 50 | Napoli MV sediment clone Napoli-3B-22 (AY592698)  99% (169/170) | *Dehalogenimonas lykanthroporepellens* BL-DC-9 (CP002084)  95% (161/170) | *Chloroflexi* |
|  |  |  | 51 | Pacific Ocean sediment clone ALVIN24a_4C_FF  97% (164/167) | *-* | *Planctomycetes* |
|  |  |  | 52 | Bioreactor clone b3-2 (EF520746) 96% (102/112) | *Paracoccus sphaerophysae Zy-3 (GU129567)*  *95% (107/113)* | *Alphaproteobacteria* |
|  |  |  | 53a | Amsterdam MV sediment clone AMSMV-25-B29 (HQ588598) 100% (185/185) | - | OP8 |
| **Bonjardim**  (131, 1.67 m) | Methanol | 4/6 | 1 | *Pseudomonas* sp. CR74 (JQ791192)  98% (191/194) | *Pseudomonas* sp. CR74 (JQ791192)  98% (191/194) | *Gammaproteobacteria* |
|  |  |  | 4 | *Petrasma* sp. gill tissue associated DGGE band Mek MV_3_11 (FM213451)  100% (194/194) | *Delftia acidovorans* strain IAC/BECa-020 (JX155410)  99% (193/194) | *Betaproteobacteria* |
|  |  |  | 9 | *Paramuricea clavata* associated clone Pc1_ScaE08 (JX874766)  99% (173/174) | *Tomitella biformata* strain AHU1820 (AB491284)  99% (172/174) | *Actinobacteria* |
|  |  |  | 10 | Porcupine Seabight sediment clone 3H3C_66 (JN230155)  100% (169/169) | *Sphingobium yanoikuyae* strain A12 (JX122496)  100% (169/169) | *Alphaproteobacteria* |
| **Captain Arutyunov**  (191, 0.52 m) | Methanol | n.a. | - | - | - | - |
| **Captain Arutyunov**  (191, 0.52 m) | Methylamine | 4/4 | 58 | *Desulfocapsa sulfexigens* DSM10523 (NR_026423)  96% (187/195) | *Desulfocapsa sulfexigens* DSM10523 (NR_026423)  96% (187/195) | *Deltaproteobacteria* |
|  |  |  | 59 | Amsterdam MV sediment clone AMSMV-0-B113 (HQ588398) 98% (192/195) | *Desulfotalea psychrophila* strain LSv54 (CR522870)  95% (185/195) | *Deltaproteobacteria* |
|  |  |  | 62a | Gulf of Mexico sediment clone GoM140_Bac16 (AM746086) 97% (190/195) | - | JS1 |
|  |  |  | 60 | Lignite mine sediment clone MEBA36a (JF973647)  99% (193/194) | *Burkholderia silvatlantica* strain LMG23149 (HQ849102)  98% (190/194) | *Betaproteobacteria* |
| **Captain Arutyunov**  (191, 0.52 m) | Hydrogen | n.a. | - | - | - | - |
| **Captain Arutyunov**  (191, 1.77 m) | Methylamine | 2 | 59d | Amsterdam MV sediment clone AMSMV-0-B113 (HQ588398) 98% (192/195) | *Desulfotalea psychrophila* strain LSv54 (CR522870)  95% (185/195) | *Deltaproteobacteria* |
|  |  |  | 62d | Gulf of Mexico sediment clone GoM140_Bac16 (AM746086) 97% (190/195) | - | JS1 |
| **Captain Arutyunov**  (206, 0.77 m) | Methylamine | 6/7 | 64 | Shimokita Peninsula sediment clone C9001C_B03_2_C045 (AB645120)  97% (190/195) | - | JS1 |
|  |  |  | 65 | Gulf of Mexico sediment clone GoM140_Bac16 (AM746086)  96% (188/195) | - | JS1 |
|  |  |  | 13a | Amsterdam MV sediment clone AMSMV-30-B5 (HQ588613) 100% (195/195) | - | JS1 |
|  |  |  | 66 | Lignite mine sediment clone MEBA36a (JF973647)  99% (193/194) | *Burkholderia silvatlantica* strain LMG23149 (HQ849102)  98% (190/194) | *Betaproteobacteria* |
|  |  |  | 67 | *Burkholderia phenazinium* strain 1S1 (AJ574888)  99% (193/194) | *Burkholderia phenazinium* strain 1S1 (AJ574888)  99% (193/194) | *Betaproteobacteria* |
|  |  |  | 87 | Amsterdam MV sediment clone AMSMV-25-B29 (HQ588598) 99% (184/185) | - | OP8 |
| **Captain Arutyunov**  (206, 2.22 m) | Methylamine | 3/3 | 29 | *Pseudomonas* sp. Wr1A04 (JF487994)  99% (194/195) | *Pseudomonas* sp. Wr1A04 (JF487994)  99% (194/195) | *Gammaproteobacteria* |
|  |  |  | 30 | Eel River Basin sediment clone so4B17 (FJ264774)  97% (165/170) | *Clostridium caminithermale* strain DVird3 (NR_041887)  91% (154/170) | *Firmicutes* |
|  |  |  | 31 | Porcupine Seabight sediment clone 3H3C_66 (JN230155)  100% (169/169) | *Sphingobium yanoikuyae* strain A12 (JX122496)  100% (169/169) | *Alphaproteobacteria* |
| **Captain Arutyunov**  (227, 0.77 m) | Methanol | n.a. | - | - | - | - |
| **Captain Arutyunov**  (227, 0.77 m) | Methylamine | n.a. | - | - | - | - |
| **Captain Arutyunov**  (227, 1.77 m) | Methanol | n.a. | - | - | - | - |
| **Captain Arutyunov**  (227, 1.77 m) | Methylamine | n.a. | - | - | - | - |
| **Darwin**  (036, 0.11 m) | Methanol | 5/9 | B16b | Rainbow hydrothermal vent sediment clone pIR3BG06 (AY354183)  95% (185/195) | *Desulfopila sp. PR5_C11* (HE600872)  92% (182/197) | *Deltaproteobacteria* |
|  |  |  | B40 | Mid-Atlantic Ridge hydrothermal vent clone TS-58 (FR839314)  99% (168/169) | *Sulfurimonas autotrophica* strain DSM16294 (CP002205)  98% (166/169) | *Epsilonproteobacteria* |
|  |  |  | B12b | Napoli MV sediment clone Napoli-4B-65 (AY592780)  98% (166/170) | *Dehalogenimonas lykanthroporepellens* BL-DC-9 (CP002084)  93% (158/170) | *Chloroflexi* |
|  |  |  | B41 | Eel River Basin sediment clone Fe_B_136 (GQ356953)  95% (185/195) | *Desulfuromonas svalbardensis* strain 103 (AY835391)  95% (185/195) | *Deltaproteobacteria* |
|  |  |  | B42 | Amsterdam MV clone AMSMV-20-B81 (HQ588589)  100% (171/171) | *Geosporobacter subterraneus* strain VNs68 (DQ643978)  99 % (169/171) | *Firmicutes* |
| **Darwin**  (036, 0.11 m) | Methylamine | 5/9 | B16a | Rainbow hydrothermal vent sediment clone pIR3BG06 (AY354183)  95% (185/195) | *Desulfopila sp. PR5_C11* (HE600872)  92% (182/197) | *Deltaproteobacteria* |
|  |  |  | B26 | Mid-Atlantic Ridge hydrothermal vent clone TS-58 (FR839314)  100% (169/169) | *Sulfurimonas autotrophica* strain DSM16294 (CP002205)  99% (167/169) | *Epsilonproteobacteria* |
|  |  |  | B12a | Napoli MV sediment clone Napoli-4B-65 (AY592780)  98% (166/170) | *Dehalogenimonas lykanthroporepellens* BL-DC-9 (CP002084)  93% (158/170) | *Chloroflexi* |
|  |  |  | B27 | Eel River Basin sediment clone Fe_B_136 (GQ356953)  94% (183/195) | *Desulfuromonas svalbardensis* strain 103 (AY835391)  94% (183/195) | *Deltaproteobacteria* |
|  |  |  | B28 | Amsterdam MV clone AMSMV-20-B81 (HQ588589)  99% (170/171) | *Geosporobacter subterraneus* strain VNs68 (DQ643978)  98 % (168/171) | *Firmicutes* |
| **Meknes**  (306, 0.77 m) | Acetate | n.a. | - | - | - | - |
| **Meknes**  (306, 0.77 m) | Benzoate | n.a. | - | - | - | - |
| **Meknes**  (306, 0.77 m) | Hexadecane | n.a. | - | - | - | - |
| **Meknes**  (306, 0.77 m) | Methanol | 5/8 | 70 | Aarhus Bay sediment slurry clone BactSlurry_JA6 (FR695384)  99% (193/194) | *Desulfovibrio oceani* subsp. *galateae* strain I9 (FJ655908)  99% (193/194) | *Deltaproteobacteria* |
|  |  |  | 71 | Wadden Sea bacterium N5IX (AJ786047)  98% (166/170) | *Acidaminobacter* sp. CJ5 (GU570195)  92% (157/170) | *Firmicutes* |
|  |  |  | 72 | Lignite mine sediment clone MEBA36a (JF973647)  98% (191/194) | *Burkholderia silvatlantica* strain LMG23149 (HQ849102)  97% (188/194) | *Betaproteobacteria* |
|  |  |  | 73 | Lignite mine sediment clone MEBA65 (JF973656)  100% (194/194) | *Burkholderia phenazinium* strain 1S1 (AJ574888)  100% (194/194) | *Betaproteobacteria* |
|  |  |  | 74 | Amsterdam MV clone AMSMV-20-B81 (HQ588589)  99% (170/171) | *Geosporobacter subterraneus* strain VNs68 (DQ643978)  98 % (168/171) | *Firmicutes* |
| **Meknes**  (306, 0.77 m) | Methylamine | 5/7 | 75 | Aarhus Bay sediment slurry clone BactSlurry_JA6 (FR695384)  99% (193/194) | *Desulfovibrio oceani* subsp. *galateae* strain I9 (FJ655908)  99% (193/194) | *Deltaproteobacteria* |
|  |  |  | 71d | Wadden Sea bacterium N5IX (AJ786047)  98% (167/170) | *Acidaminobacter* sp. CJ5 (GU570195)  92% (156/170) | *Firmicutes* |
|  |  |  | 72a | Lignite mine sediment clone MEBA36a (JF973647)  99% (193/194) | *Burkholderia silvatlantica* strain LMG23149 (HQ849102)  97% (188/194) | *Betaproteobacteria* |
|  |  |  | 76 | Lignite mine sediment clone MEBA65 (JF973656)  100% (194/194) | *Burkholderia phenazinium* strain 1S1 (AJ574888)  100% (194/194) | *Betaproteobacteria* |
|  |  |  | 77 | Amsterdam MV clone AMSMV-20-B81 (HQ588589)  99% (170/171) | *Geosporobacter subterraneus* strain VNs68 (DQ643978)  98 % (168/171) | *Firmicutes* |
| **Meknes**  (306, 1.82 m) | Acetate | n.a. | - | - | *-* | - |
| **Mekne**s  (306, 1.82 m) | Methylamine | n.a. | - | - | *-* | - |
| **Mercator**  (238, 0.72 m) | Methylamine | n.a. | - | - | *-* | - |
| **Mercator**  (009, 0.13 m) | Hexadecane | n.a. | - | - | *-* | - |
| **Mercator**  (013, 0.15 m) | Acetate | 2/2 | B4 | Aarhus Bay sediment clone BactSlurry_B1 (FR695360)  89% (172/194) | - | *Gammaproteobacteria* |
|  |  |  | B8 | Gulf of Cadiz MV sediment clone GoC_Bac_122_D1_C0_M0 (FN820323)  95% (187/196) | *Desulfopila* sp. PR2_A10 (HE600883)  95% (187/195) | *Deltaproteobacteria* |
| **Mercator**  (013, 0.15 m) | Benzoate | n.a. | - | - | *-* | - |
| **Mercator**  (013, 0.15 m) | Hexadecane | 1 | B8 | Gulf of Cadiz MV sediment clone GoC_Bac_122_D1_C0_M0 (FN820323)  95% (187/196) | *Desulfopila* sp. PR2_A10 (HE600883)  95% (187/195) | *Deltaproteobacteria* |
| **Mercator**  (015, 0.30 m) | Methanol | 6/7 | B7 | Oil-polluted sediment clone FII-OX093 (JQ579742)  95% (180/189) | *Marinifilum* sp. KYW 585 (JQ687110)  95% (179/189) | *Bacteroidetes* |
|  |  |  | B8 | Gulf of Cadiz MV sediment clone GoC_Bac_122_D1_C0_M0 (FN820323)  95% (187/196) | *Desulfopila* sp. PR2_A10 (HE600883)  95% (187/195) | *Deltaproteobacteria* |
|  |  |  | B9 | Colne Estuary sediment clone CBII115 (DQ831556)  95% (185/195) | *Desulfopila* sp. PR2_A10 (HE600883)  95% (185/195) | *Deltaproteobacteria* |
|  |  |  | B10 | Eel River Basin sediment clone Fe_B_122 (GQ356939)  98% (167/170) | *Clostridium caminithermale* strain DVird3 (NR_041887)  92% (156/170) | *Firmicutes* |
|  |  |  | B11 | Amsterdam MV sediment clone Amsterdam-2B-43 (AY592401)  99% (193/195) | *Desulfobulbus japonicus* strain DSM 18378 (NR_040977)  93% (181/195) | *Deltaproteobacteria* |
|  |  |  | B12 | Napoli MV sediment clone Napoli-4B-65 (AY592780)  95% (162/170) | *Dehalogenimonas lykanthroporepellens* BL-DC-9 (CP002084)  92% (156/170) | *Chloroflexi* |
| **Mercator**  (019, 0.18 m) | Acetate | n.a. | - | - | *-* | - |
| **Mercator**  (019, 1.90 m) | Benzoate | n.a. | - | - | *-* | - |
| **Mercator**  (019, 1.90 m) | Hexadecane | n.a. | - | - | *-* | - |
| **Mercator**  (019, 2.23 m) | Hexadecane | 3/3 | B4 | Aarhus Bay sediment clone BactSlurry_B1 (FR695360)  89% (172/194) | - | *Gammaproteobacteria* |
|  |  |  | B5 | Nankai Forearc Basin sediment clone MB-B2-105 (AY093470)  98% (167/171) | *Calothrix* sp. LCRSM-1413 (JN705664)  96% (164/171) | *Cyanobacteria* |
|  |  |  | B6 | Soybean leaf clone M05_1_E09 (AB582247)  98% (192/195) | - | *Deltaproteobacteria* |

n.a. = no PCR amplification for this sediment slurry

See Figure 4 and Table 2 for further information.
