# Supplementary material for: Methanogen activity and microbial diversity in Gulf of Cádiz mud volcano sediments
Source: Front Microbiol. 2023 May 24;14:1157337. doi: 10.3389/fmicb.2023.1157337 (PMC10244519; doi:10.3389/fmicb.2023.1157337)
Supplement: Supplementary file 6 [file Table_5.DOCX]

**Supplementary Table S5.** Mean methanogenic activity rates (n=3) for different ^14^C-substrates (acetate, methanol, methylamine and bicarbonate) in MV sediments from the Gulf of Cádiz - Carlos Ribeiro MV, Captain Arutyunov MV, Darwin MV and Mercator MV. mbsf = metres below seafloor (See supplementary Figure S5).

| **Mud volcano** | **Station** | **Depth (mbsf)** | **Methanogenesis (pmol/cm^3^/d)** | | | |
| --- | --- | --- | --- | --- | --- | --- |
|  |  |  | **Acetate** | **Bicarbonate** | **Methanol** | **Methylamine** |
| **Mercator** | JC10-002 | 0.030 | 0.000 | 0.000 | 0.000 | 0.372 |
|  |  | 0.180 | 0.000 | 0.000 | 0.000 | 0.051 |
|  | JC10-004 | 0.425 | 0.000 | 0.000 | 0.000 | 0.000 |
|  |  | 1.275 | 0.000 | 0.000 | 0.000 | 0.000 |
|  |  | 2.275 | 0.000 | 0.000 | 0.000 | 0.000 |
|  |  | 3.475 | 0.000 | 0.000 | 0.000 | 0.000 |
|  | JC10-009 | 0.025 | 0.000 | 0.000 | 0.000 | 0.000 |
|  |  | 0.125 | 0.000 | 0.000 | 0.000 | 0.000 |
|  |  | 0.225 | 0.000 | 0.000 | 0.000 | 0.000 |
|  |  | 0.325 | 0.000 | 0.000 | 0.000 | 0.000 |
|  |  | 0.425 | 0.000 | 0.000 | 0.000 | 0.000 |
|  |  | 0.525 | 0.000 | 0.000 | 0.000 | 0.000 |
|  |  | 0.625 | 0.000 | 0.000 | 0.000 | 0.000 |
|  |  | 0.725 | 0.000 | 0.000 | 0.000 | 0.000 |
|  | JC10-011 | 0.030 | 0.000 | 0.000 | 0.000 | 1.204 |
|  |  | 0.130 | 0.000 | 0.000 | 0.000 | 3.023 |
|  |  | 0.170 | 0.000 | 0.000 | 0.000 | 0.000 |
|  |  | 0.330 | 0.000 | 0.000 | 0.000 | 0.000 |
|  |  | 0.370 | 0.000 | 0.000 | 0.000 | 0.000 |
|  | JC10-013 | 0.030 | 0.000 | 0.000 | 0.000 | 8.068 |
|  |  | 0.130 | 2.258 | 0.000 | 0.000 | 0.000 |
|  |  | 0.170 | 0.047 | 50.542 | 0.000 | 0.000 |
|  |  | 0.250 | 0.000 | 0.000 | 0.000 | 0.000 |
|  | JC10-015 | 0.125 | 0.000 | 0.000 | 0.000 | 0.036 |
|  |  | 0.275 | 0.000 | 0.000 | 0.000 | 0.000 |
|  |  | 0.425 | 0.000 | 0.000 | 0.000 | 0.000 |
|  |  | 0.575 | 0.000 | 0.000 | 0.000 | 0.000 |
|  |  | 0.725 | 0.000 | 0.000 | 0.000 | 0.000 |
|  | JC10-019 | 0.125 | 0.000 | 0.000 | 0.000 | 0.000 |
|  |  | 0.225 | 0.000 | 0.000 | 0.000 | 0.000 |
|  |  | 0.325 | 0.000 | 0.000 | 0.000 | 0.000 |
|  |  | 0.425 | 0.000 | 0.000 | 0.000 | 0.000 |
|  |  | 0.525 | 0.000 | 0.000 | 0.000 | 0.000 |
|  |  | 0.625 | 0.000 | 0.000 | 0.000 | 0.000 |
|  |  | 1.125 | 0.000 | 0.000 | 0.000 | 0.000 |
|  |  | 1.625 | 0.000 | 0.000 | 0.000 | 0.000 |
|  |  | 1.875 | 0.000 | 0.000 | 0.000 | 0.000 |
|  |  | 2.225 | 0.000 | 0.000 | 0.000 | 0.000 |
| **Darwin** | JC10-025 | 0.305 | 0.114 | 0.000 | 0.000 | 0.000 |
|  |  | 0.555 | 0.000 | 0.000 | 0.000 | 0.000 |
|  |  | 1.205 | 0.000 | 0.000 | 0.000 | 0.000 |
|  |  | 1.705 | 0.000 | 0.000 | 0.000 | 0.000 |
|  |  | 2.225 | 0.000 | 0.000 | 0.000 | 0.000 |
|  |  | 2.805 | 0.000 | 0.000 | 0.000 | 0.000 |
|  |  | 3.705 | 0.000 | 0.000 | 0.000 | 0.000 |
|  |  | 4.755 | 0.000 | 0.000 | 0.000 | 0.000 |
|  | JC10-026 | 0.030 | 0.000 | 0.000 | 0.000 | 0.000 |
|  |  | 0.070 | 0.000 | 0.000 | 0.000 | 0.000 |
|  |  | 0.320 | 0.000 | 0.000 | 0.000 | 0.000 |
|  | JC10-029 | 0.025 | 0.000 | 0.000 | 0.000 | 0.000 |
|  |  | 0.125 | 0.000 | 0.994 | 0.000 | 4.639 |
|  |  | 0.225 | 0.000 | 0.000 | 0.000 | 1.435 |
|  |  | 0.325 | 0.000 | 0.000 | 0.000 | 0.002 |
|  | JC10-030 | 0.030 | 0.000 | 0.000 | 0.000 | 0.000 |
|  |  | 0.070 | 0.000 | 0.221 | 0.000 | 0.000 |
|  |  | 0.180 | 0.000 | 0.000 | 0.000 | 1.031 |
|  |  | 0.220 | 0.000 | 0.000 | 0.000 | 0.000 |
|  | JC10-038 | 0.045 | 0.000 | 0.000 | 0.000 | 5.324 |
|  |  | 0.145 | 0.000 | 0.000 | 0.000 | 0.199 |
|  |  | 0.295 | 0.000 | 0.000 | 0.000 | 1.862 |
|  |  | 0.445 | 0.000 | 0.000 | 0.000 | 0.080 |
|  |  | 0.595 | 0.000 | 0.000 | 0.000 | 0.000 |
|  |  | 0.695 | 0.000 | 0.000 | 0.000 | 0.000 |
| **Carlos Ribeiro** | JC10-044 | 0.030 | 0.000 | 0.000 | 0.000 | 0.000 |
|  |  | 0.070 | 0.000 | 0.123 | 0.000 | 0.000 |
|  |  | 0.150 | 0.000 | 0.000 | 0.000 | 0.000 |
|  | JC10-045 | 0.175 | 0.000 | 0.000 | 0.000 | 0.010 |
|  |  | 0.675 | 0.000 | 0.000 | 0.000 | 0.000 |
|  |  | 1.175 | 0.000 | 0.000 | 0.000 | 0.000 |
|  |  | 1.925 | 0.000 | 0.000 | 0.000 | 0.000 |
|  |  | 2.675 | 0.000 | 0.000 | 0.000 | 0.000 |
|  |  | 3.425 | 0.000 | 0.000 | 0.000 | 0.000 |
|  |  | 4.425 | 0.000 | 0.000 | 0.000 | 0.000 |
|  | JC10-048 | 0.155 | 0.000 | 0.000 | 0.000 | 0.189 |
|  |  | 0.255 | 0.000 | 0.000 | 0.000 | 0.000 |
|  |  | 0.355 | 0.000 | 0.000 | 0.000 | 0.000 |
|  |  | 0.455 | 0.000 | 0.000 | 0.000 | 0.000 |
|  |  | 0.555 | 0.000 | 0.000 | 0.000 | 0.000 |
|  |  | 0.705 | 0.000 | 0.000 | 0.000 | 0.000 |
|  |  | 0.855 | 0.000 | 0.000 | 0.000 | 0.000 |
|  |  | 1.005 | 0.000 | 0.000 | 0.000 | 0.000 |
|  |  | 1.205 | 0.000 | 0.000 | 0.000 | 0.000 |
|  |  | 1.405 | 0.000 | 0.000 | 0.000 | 0.000 |
|  | JC10-050 | 0.020 | 0.000 | 1.007 | 0.000 | 0.000 |
|  |  | 0.050 | 0.710 | 0.000 | 0.000 | 0.000 |
|  |  | 0.080 | 0.000 | 0.000 | 0.000 | 0.000 |
|  |  | 0.110 | 10.600 | 0.000 | 0.566 | 0.022 |
|  |  | 0.140 | 2.302 | 0.000 | 0.000 | 0.000 |
|  | JC10-053 | 0.135 | 0.000 | 0.000 | 0.000 | 0.235 |
|  |  | 0.335 | 0.000 | 0.000 | 0.000 | 0.000 |
|  |  | 0.585 | 0.000 | 0.000 | 0.000 | 0.001 |
|  |  | 0.835 | 0.000 | 0.000 | 0.000 | 0.003 |
|  |  | 1.085 | 0.000 | 0.000 | 0.000 | 0.006 |
|  |  | 1.385 | 0.000 | 0.000 | 0.000 | 0.000 |
|  |  | 1.685 | 0.000 | 0.000 | 0.000 | 0.003 |
|  |  | 1.985 | 0.000 | 0.000 | 0.000 | 0.007 |
|  |  | 2.385 | 0.000 | 0.000 | 0.000 | 0.000 |
|  |  | 2.735 | 0.000 | 0.000 | 0.000 | 0.000 |
|  |  | 3.285 | 0.000 | 0.000 | 0.000 | 0.000 |
|  |  | 3.735 | 0.000 | 0.000 | 0.000 | 0.000 |
|  |  | 4.285 | 0.000 | 0.000 | 0.000 | 0.000 |
|  |  | 4.785 | 0.000 | 0.000 | 0.000 | 0.000 |
|  |  | 5.185 | 0.000 | 0.000 | 0.000 | 0.010 |
|  | JC10-054 | 0.040 | 0.000 | 0.000 | 0.000 | 0.000 |
|  |  | 0.230 | 0.000 | 0.000 | 0.000 | 0.000 |
|  |  | 0.270 | 0.000 | 0.000 | 0.000 | 0.000 |
|  |  | 0.290 | 0.000 | 0.000 | 0.000 | 0.000 |
|  |  | 0.420 | 0.000 | 0.000 | 0.000 | 0.000 |
|  |  | 0.460 | 0.000 | 0.000 | 0.000 | 0.000 |
|  |  | 0.490 | 0.000 | 0.000 | 0.000 | 0.000 |
| **Captain Arutyunov** | JC10-066 | 0.085 | 0.000 | 0.000 | 0.000 | 2.331 |
|  |  | 0.185 | 0.000 | 0.000 | 0.000 | 1.888 |
|  |  | 0.285 | 0.000 | 0.000 | 0.000 | 0.005 |
|  |  | 0.415 | 0.000 | 0.000 | 0.000 | 0.000 |
|  |  | 0.615 | 0.134 | 0.000 | 0.000 | 0.004 |
|  |  | 0.965 | 0.000 | 0.000 | 0.000 | 0.009 |
|  |  | 1.465 | 0.000 | 0.000 | 0.000 | 0.000 |
|  |  | 2.065 | 0.000 | 0.000 | 0.000 | 0.000 |
|  |  | 2.665 | 0.000 | 0.000 | 0.000 | 0.004 |
|  |  | 3.065 | 0.120 | 0.000 | 0.000 | 0.001 |
|  |  | 3.565 | 0.000 | 0.000 | 0.000 | 0.020 |
|  |  | 4.015 | 0.000 | 0.000 | 0.763 | 0.027 |
